# Supplementary figures and images for: Adding pieces to the puzzle: insights into diversity and distribution patterns of Cumacea (Crustacea: Peracarida) from the deep North Atlantic to the Arctic Ocean
Source: PeerJ. 2021 Nov 11;9:e12379. doi: 10.7717/peerj.12379 (PMC8590803; doi:10.7717/peerj.12379)

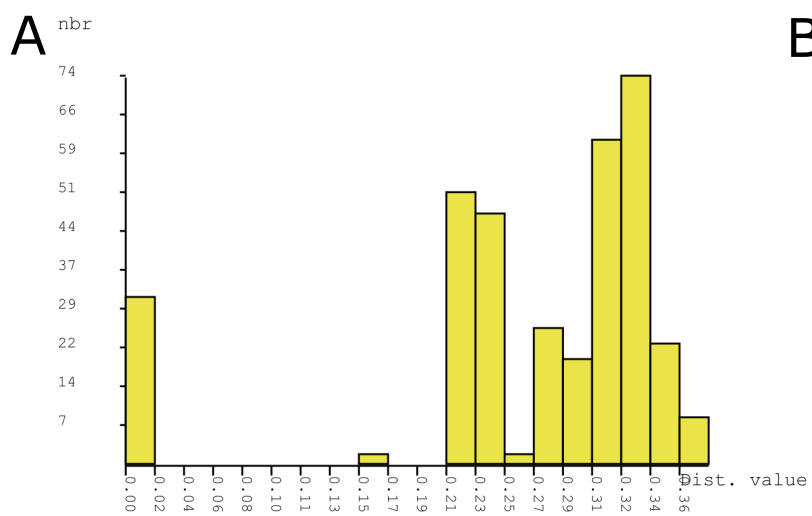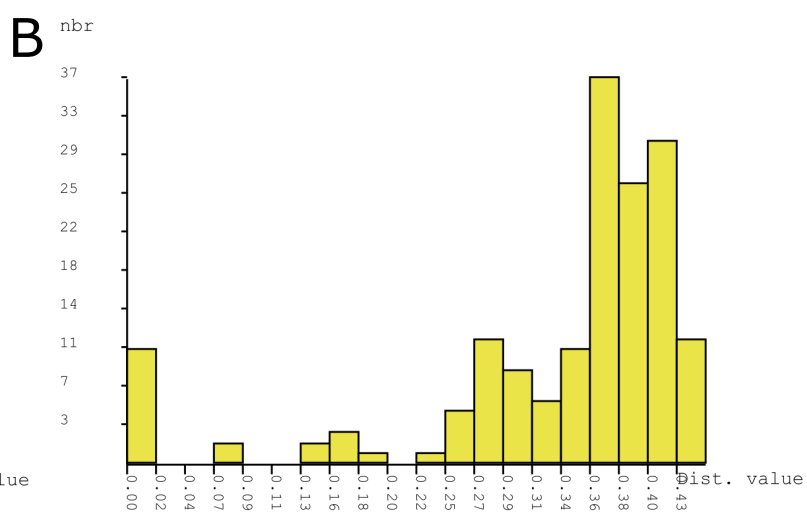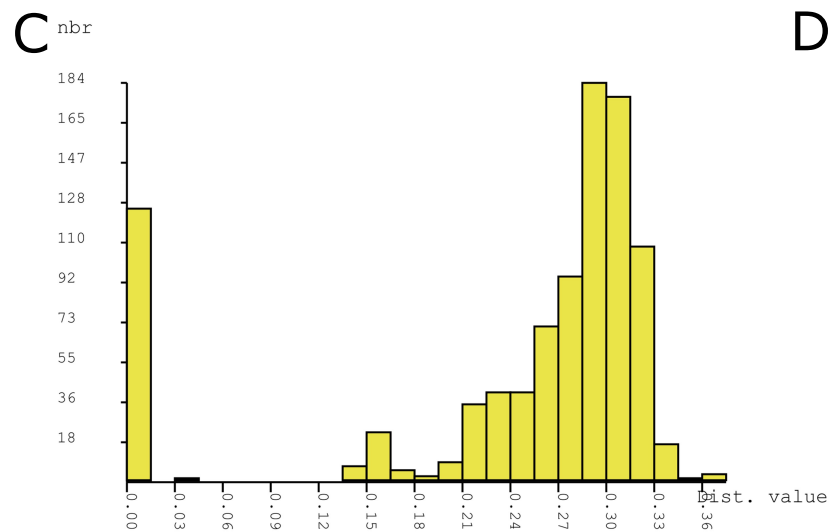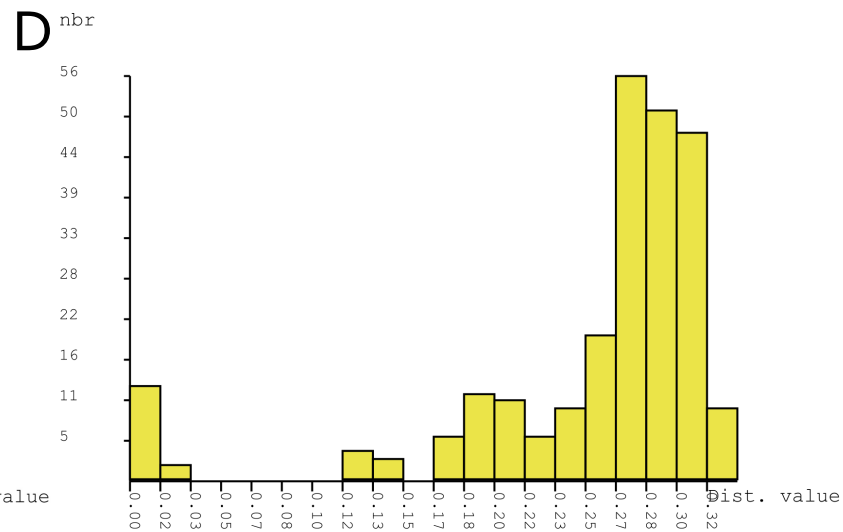

Supplement: Supplemental Information 1 — Bar plot shows a clear barcoding gap based on the applied threshold of P = 0.01–0.1 between intra- and interspecific variation for all families: (A) Leuconidae (intra: 0–0.01; inter: 0.17–0.38); (B) Bodotriidae and Nannastacidae (0–0.01; 0.08–0.45); (C) Diastylidae and Pseudocumatidae (0–0.04; 0.15–0.37); (D) Ceratocumatidae and Lampropidae (0-0.02; 0.13-0.34). [file peerj-09-12379-s001.pdf]

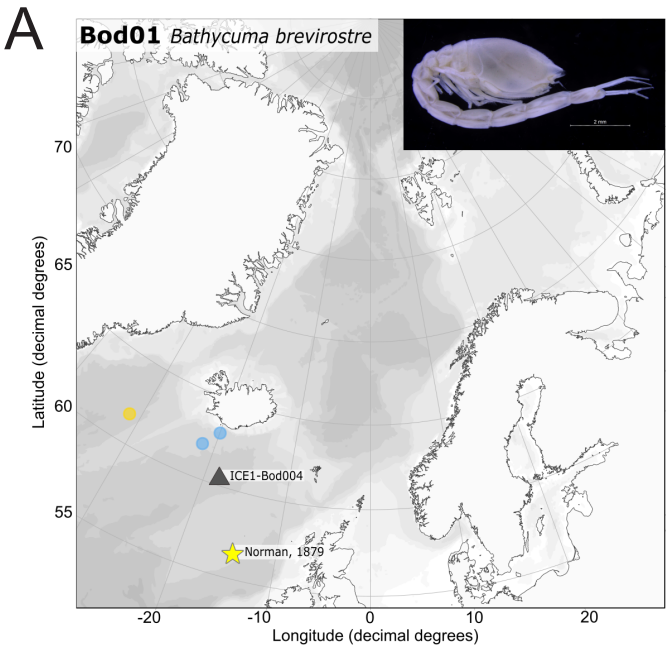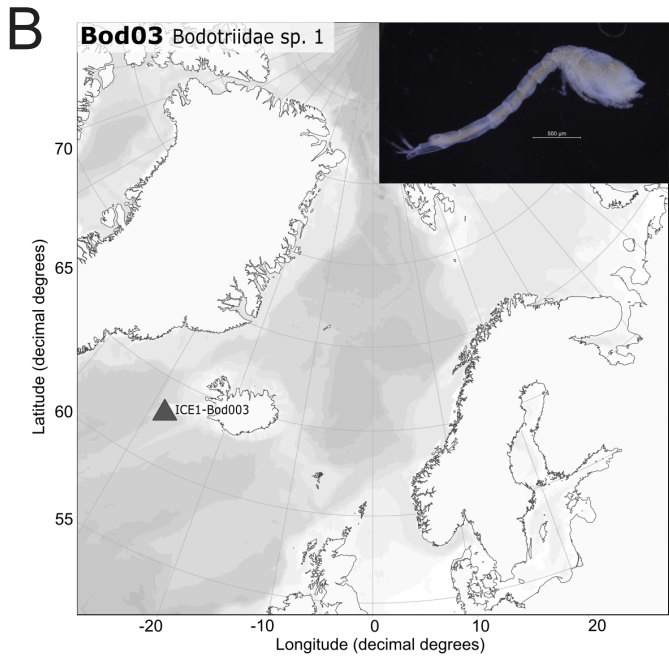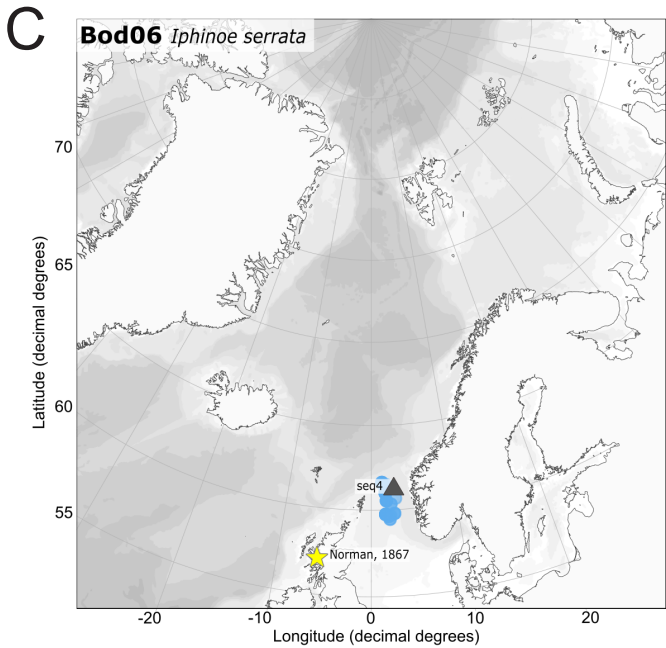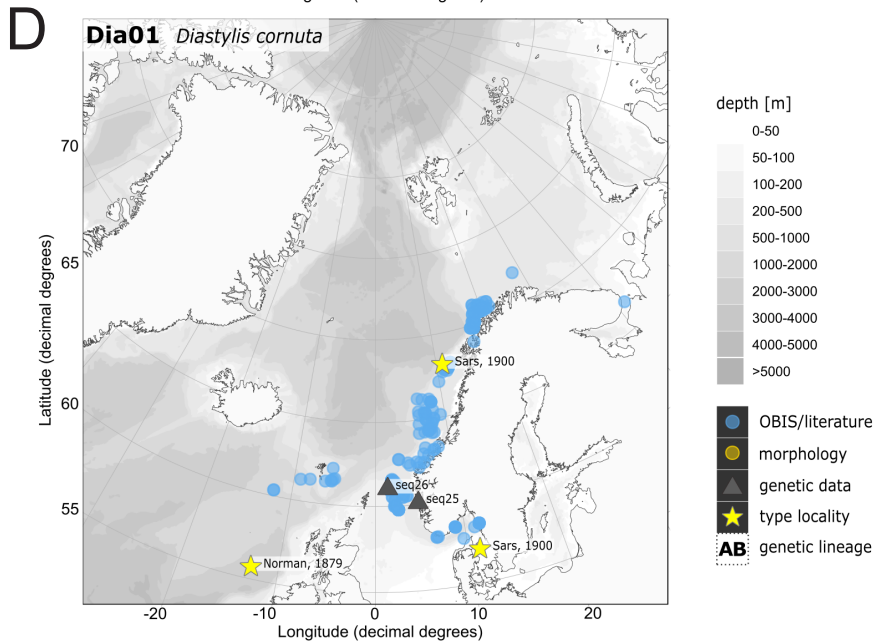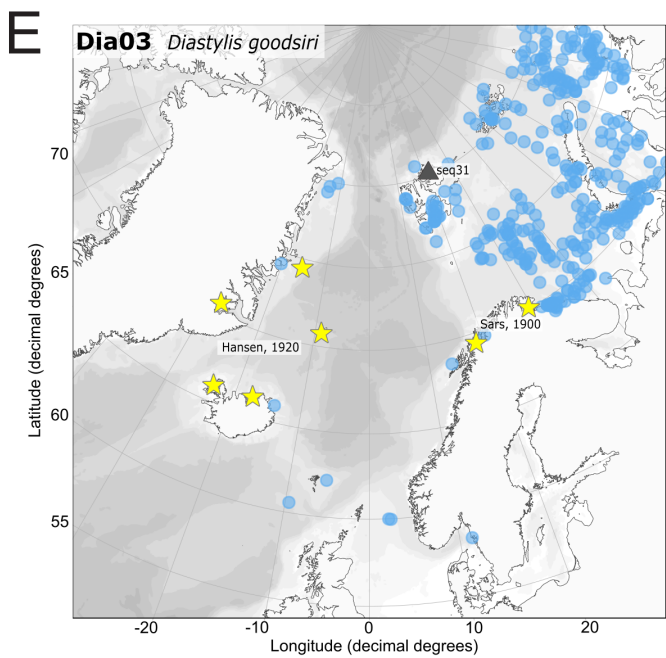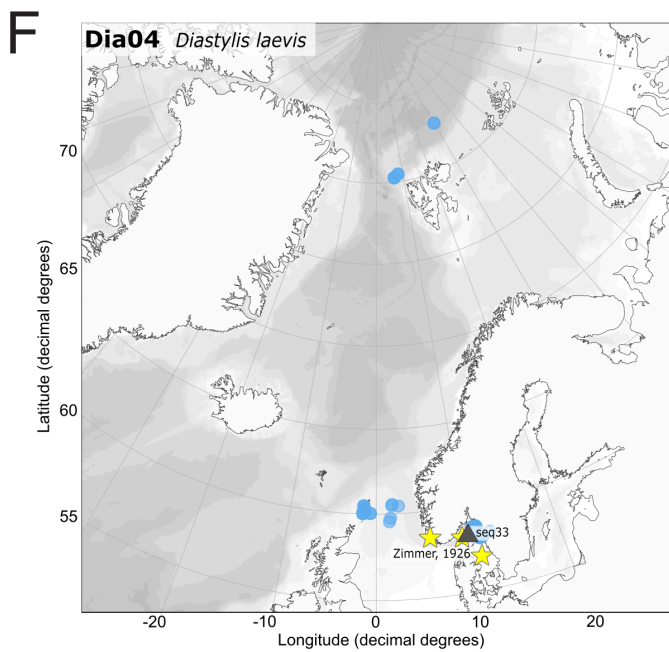

Supplement: Supplemental Information 2 — Occurrence records are shown from the MAREANO and OBIS platform as well as literature data (blue), specimens morphologically investigated in this study (orange) and subsequently genetically investigated (grey triangle with sequence ID) and type and/or syntype locality of putative species (yellow star with reference literature). [file peerj-09-12379-s002.pdf]

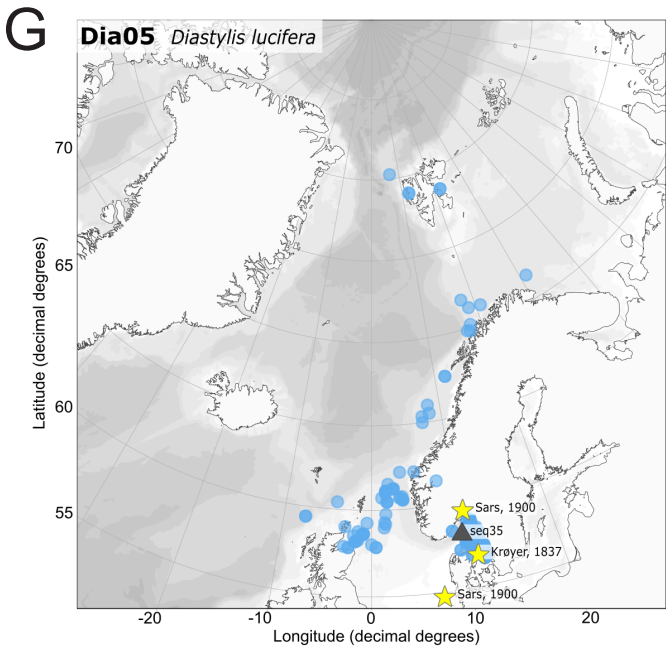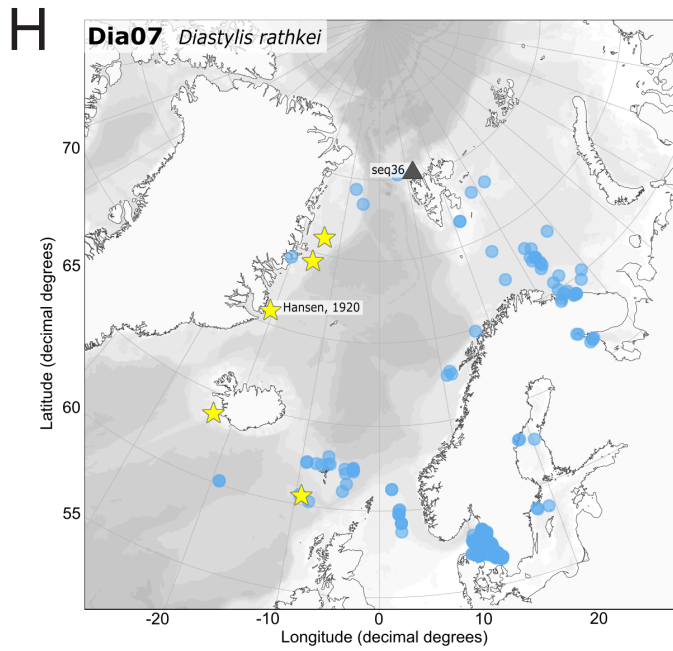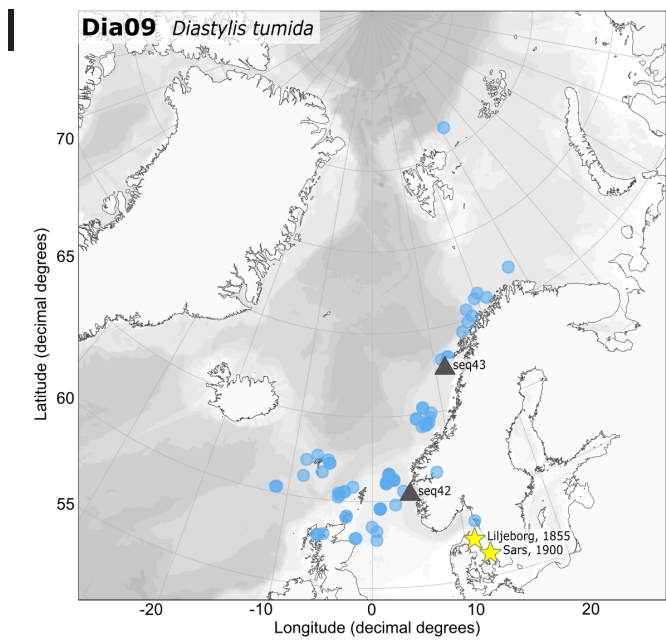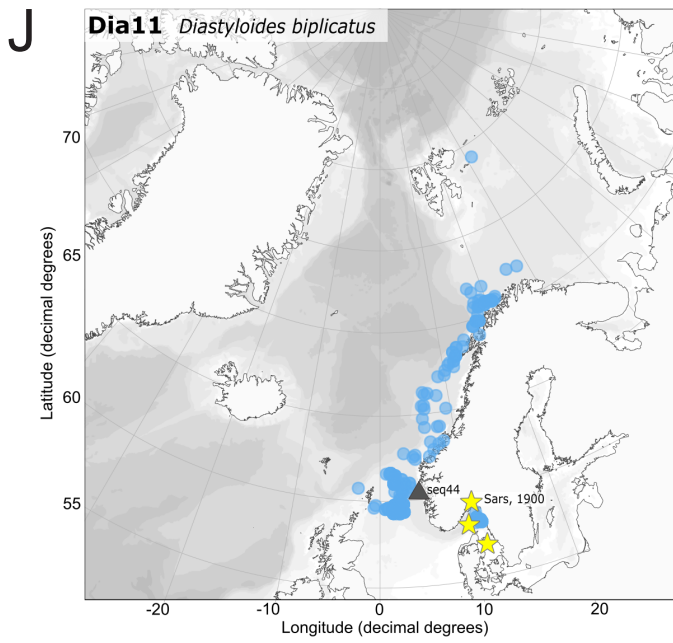

depth [m]

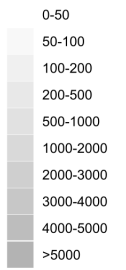

- OBIS/literature
- morphology
- genetic data
- type locality
- AB: genetic lineage

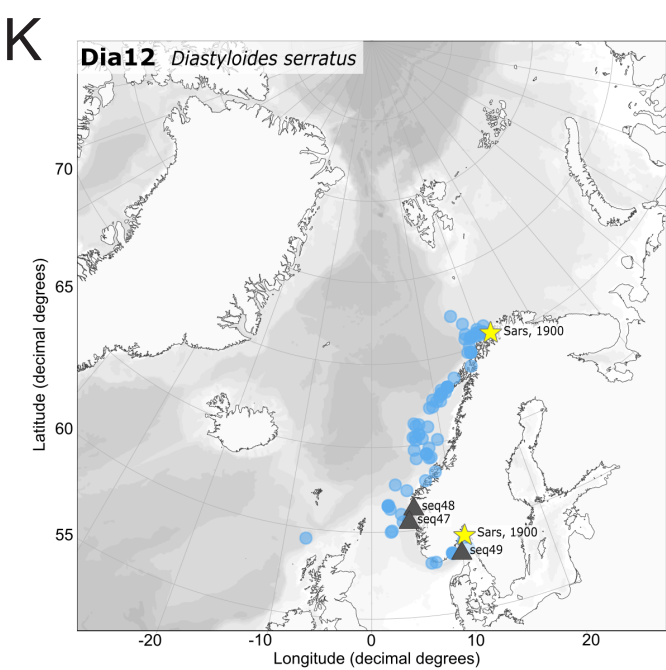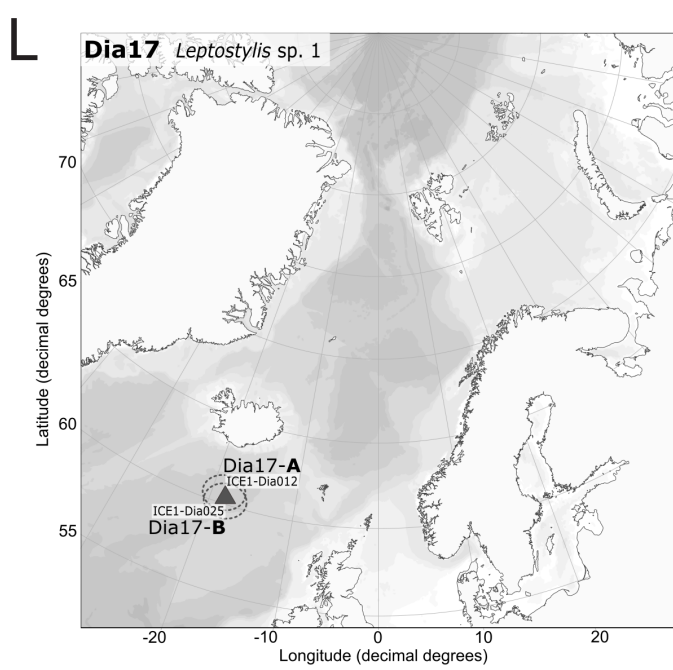

Supplement: Supplemental Information 3 — Occurrence records are shown from the MAREANO and OBIS platform as well as literature data (blue), specimens morphologically and subsequently genetically investigated (grey triangle with sequence ID) and type and/or syntype locality of putative species (yellow star with reference literature). Genetic lineages are highlighted with dotted circles and separated by assigned letters A–B. [file peerj-09-12379-s003.pdf]

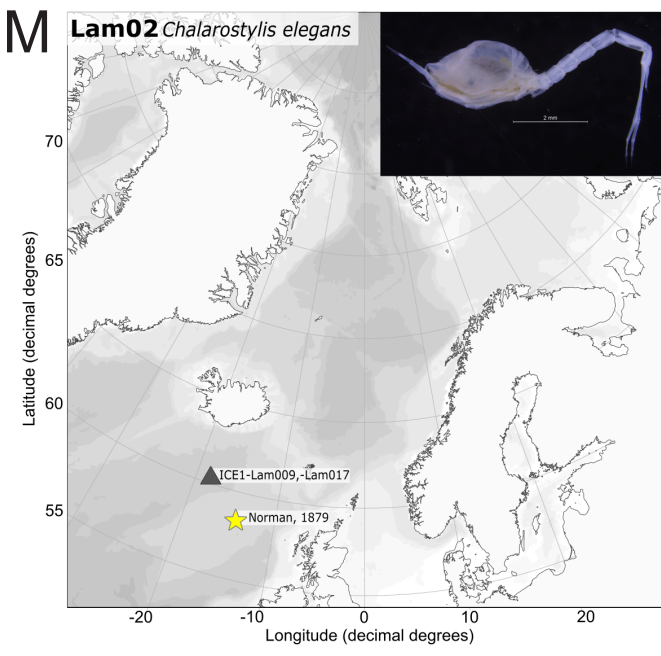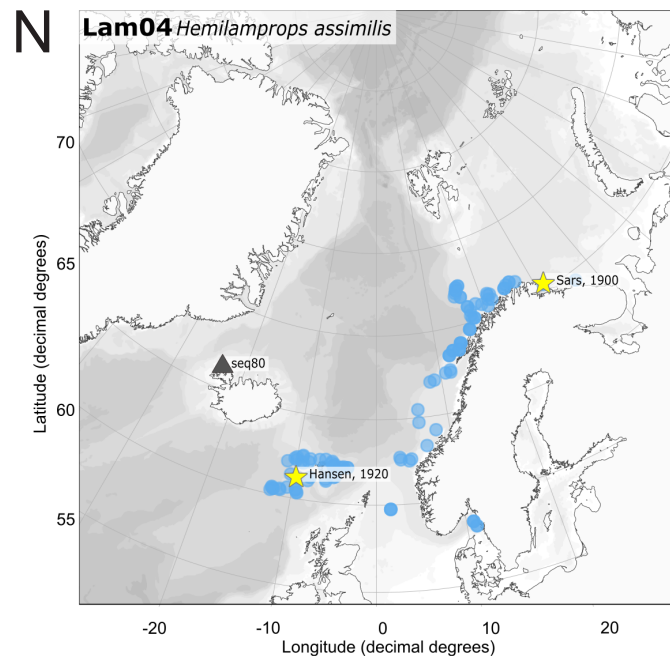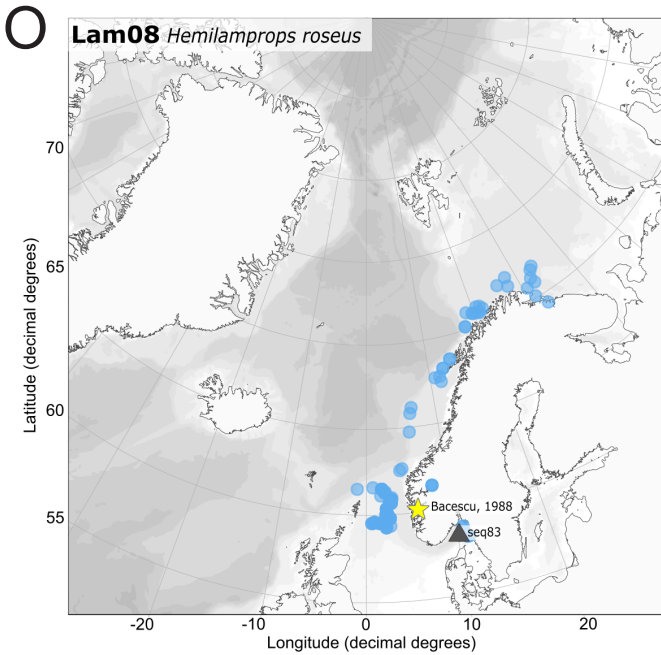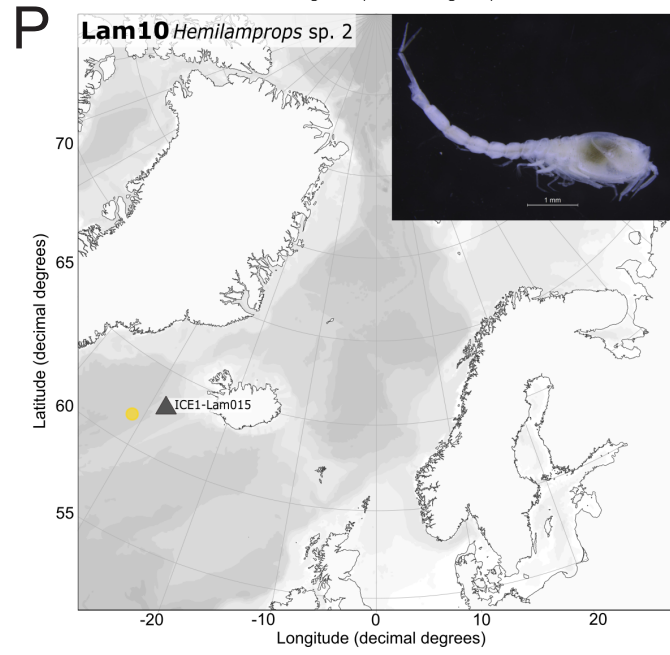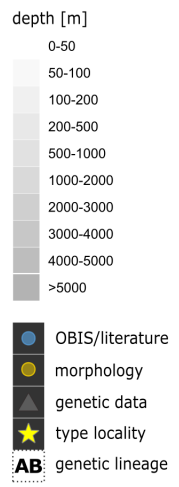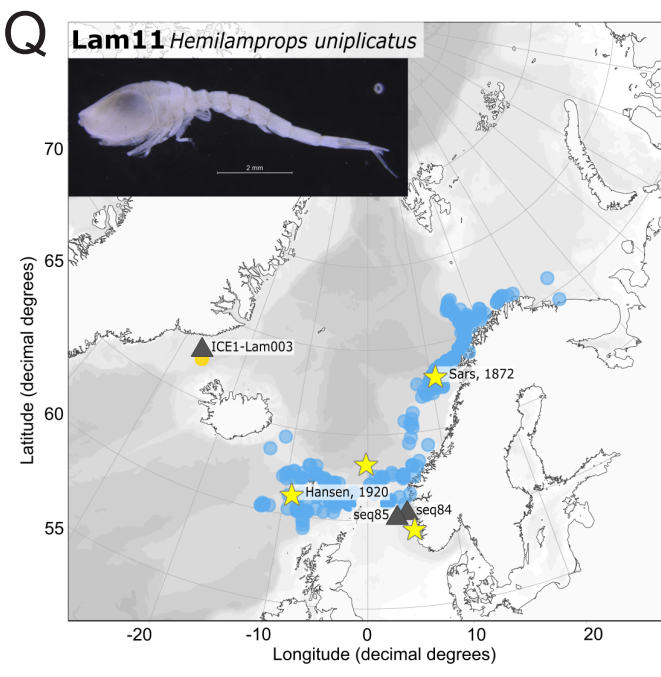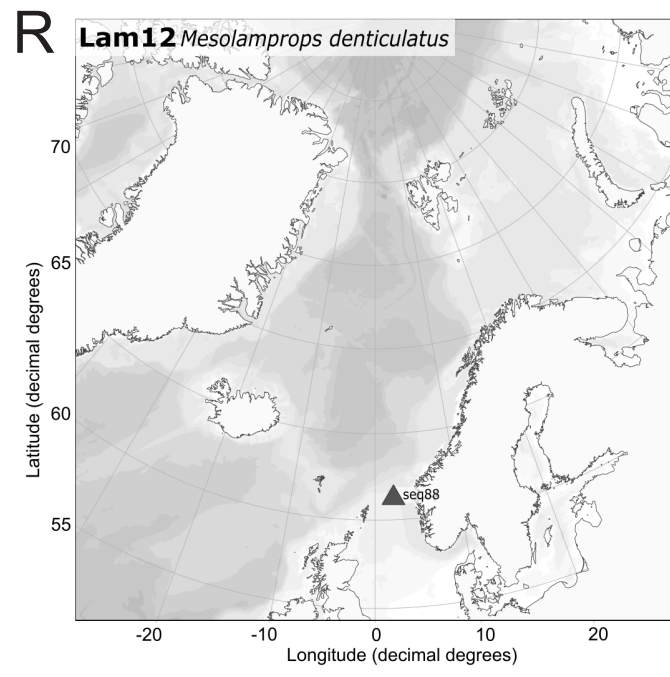

Supplement: Supplemental Information 4 — Occurrence records are shown from the MAREANO and OBIS platform as well as literature data (blue), specimens morphologically investigated in this study (orange) and subsequently genetically investigated (grey triangle with sequence ID) and type and/or syntype locality of putative species (yellow star with reference literature). [file peerj-09-12379-s004.pdf]

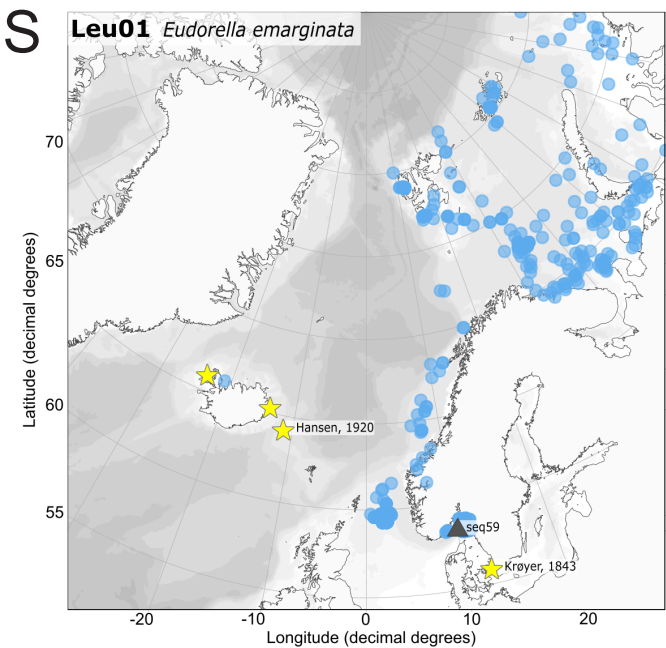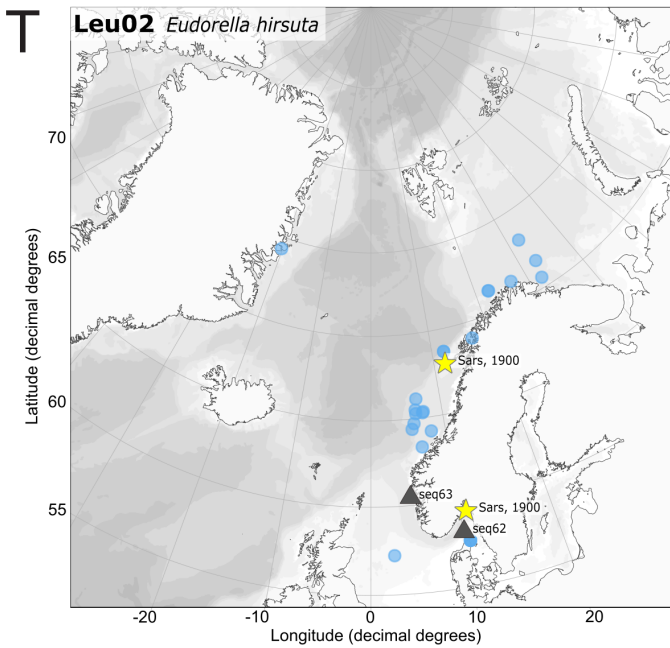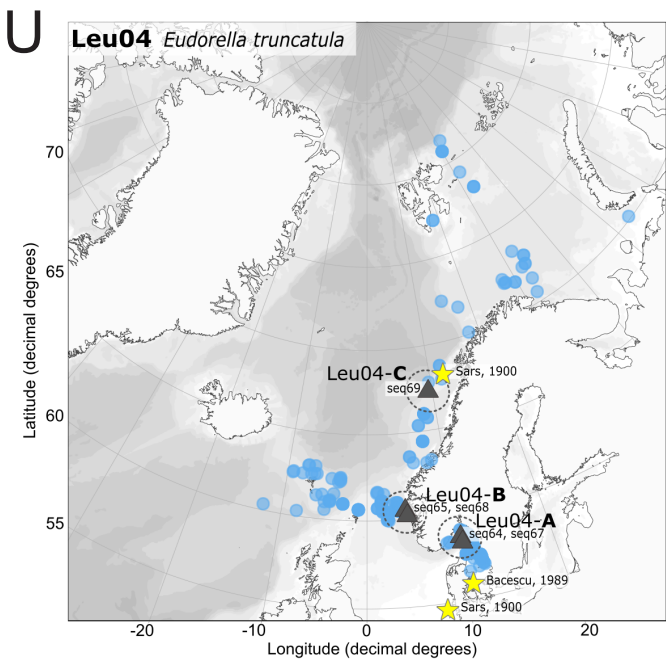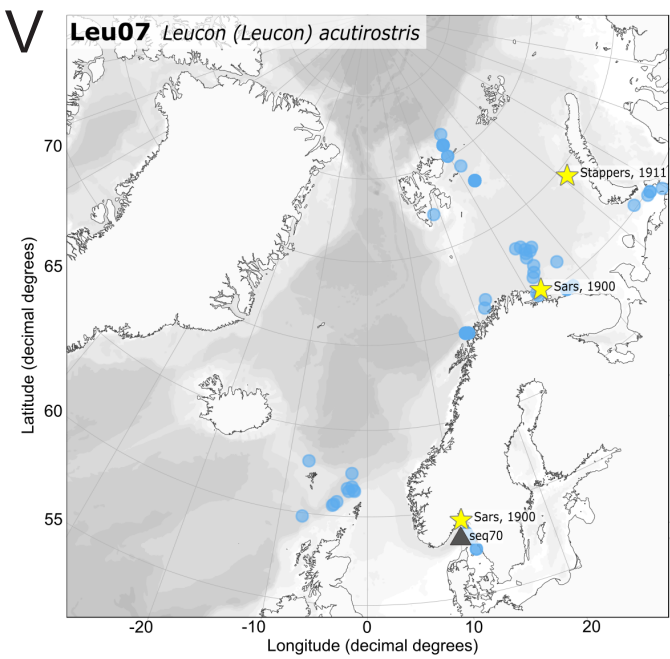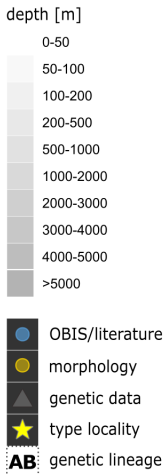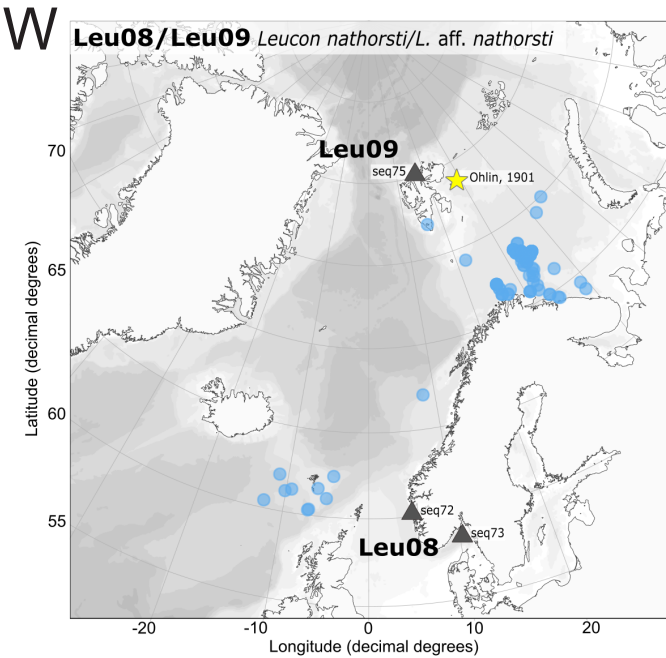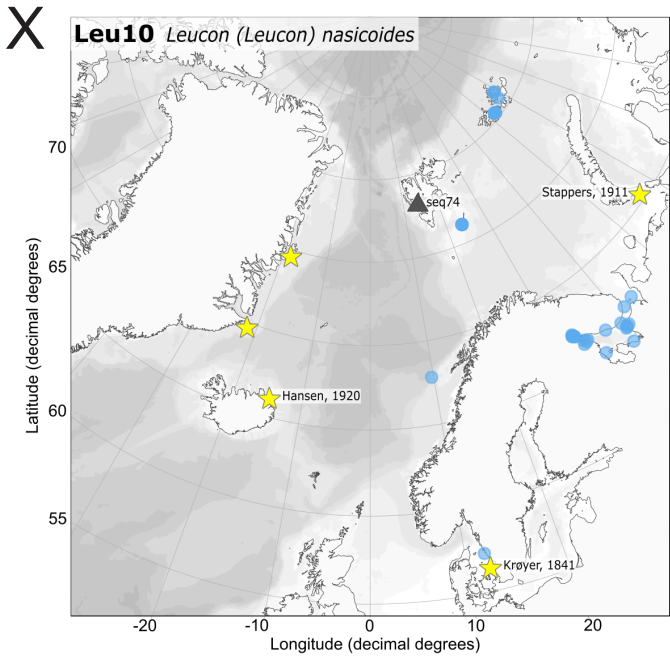

Supplement: Supplemental Information 5 — Occurrence records are shown from the MAREANO and OBIS platform as well as literature data (blue), specimens morphologically and subsequently genetically investigated (grey triangle with sequence ID) and type and/or syntype locality of putative species (yellow star with reference literature). Genetic lineages are highlighted with dotted circles and separated by assigned letters A-C. [file peerj-09-12379-s005.pdf]

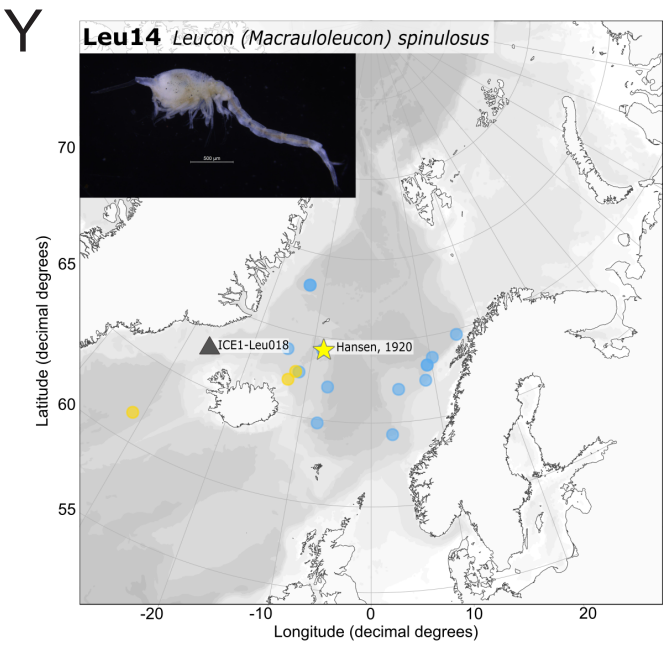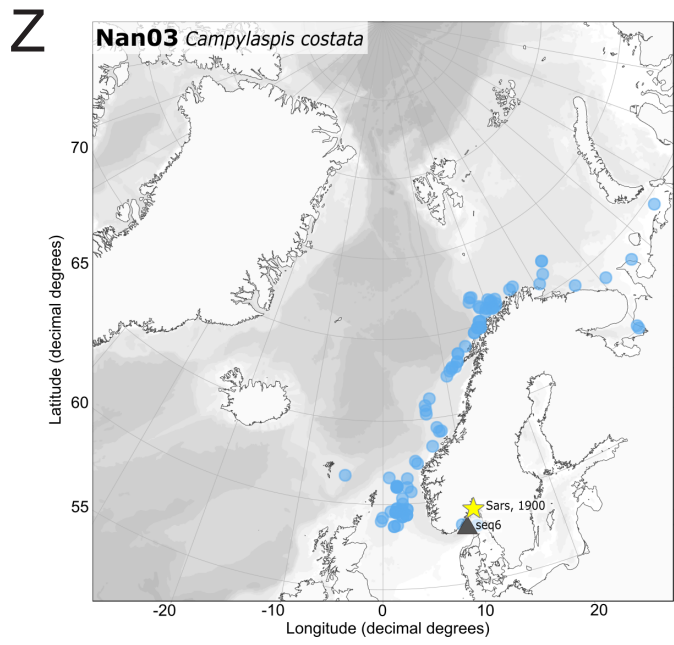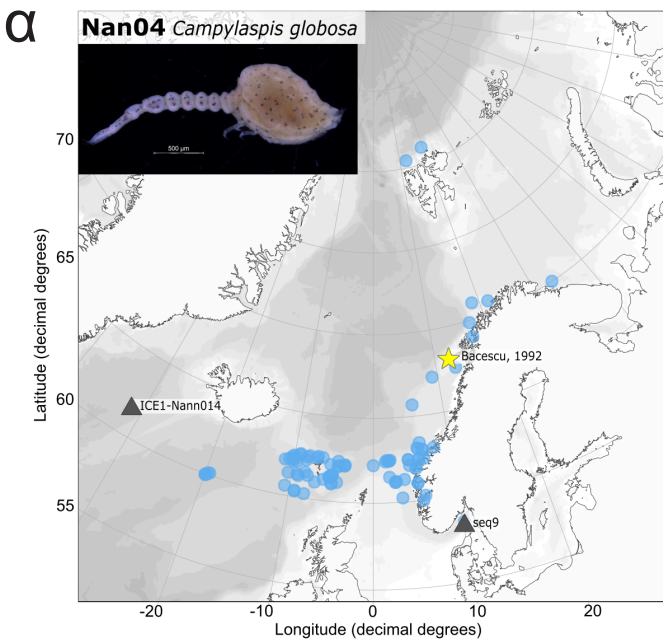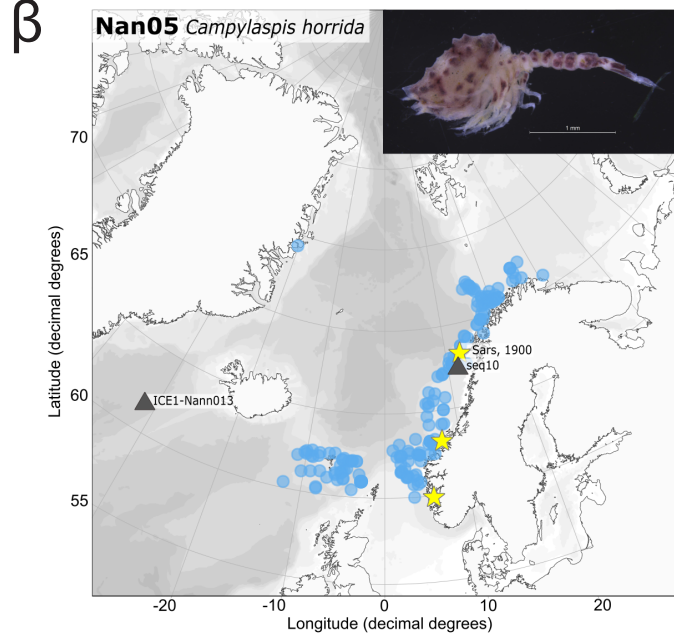

depth [m]

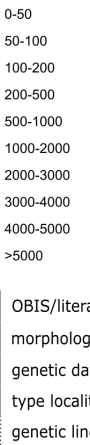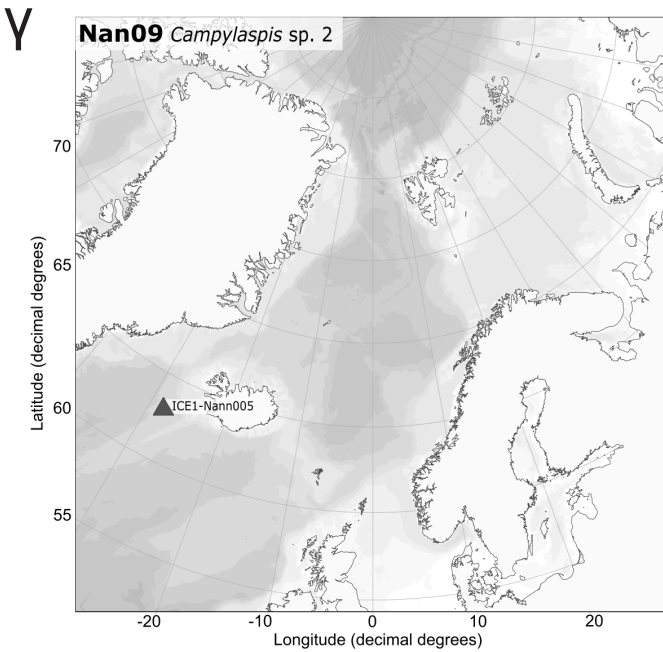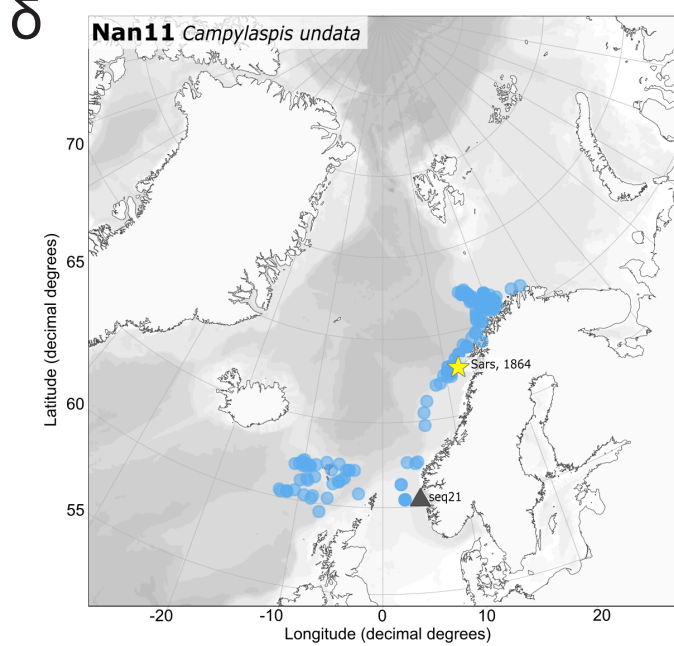

Supplement: Supplemental Information 6 — Occurrence records are shown from the MAREANO and OBIS platform as well as literature data (blue), specimens morphologically investigated in this study (orange) and subsequently genetically investigated (grey triangle with sequence ID) and type and/or syntype locality of putative species (yellow star with reference literature). [file peerj-09-12379-s006.pdf]

ε

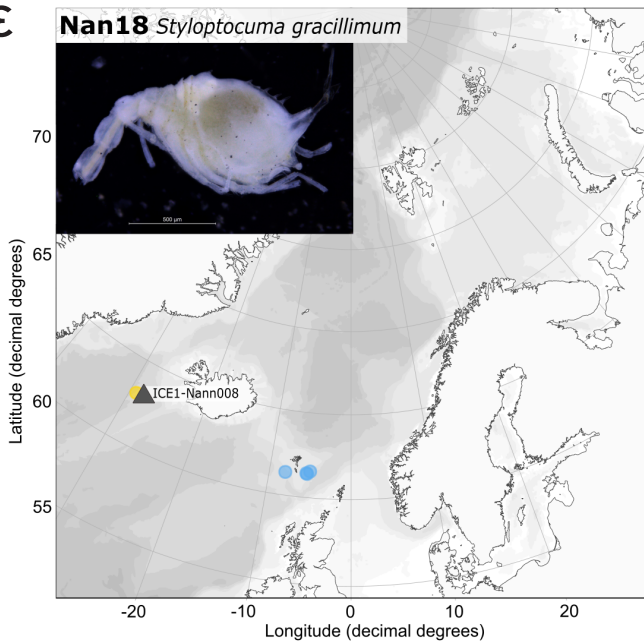

ζ

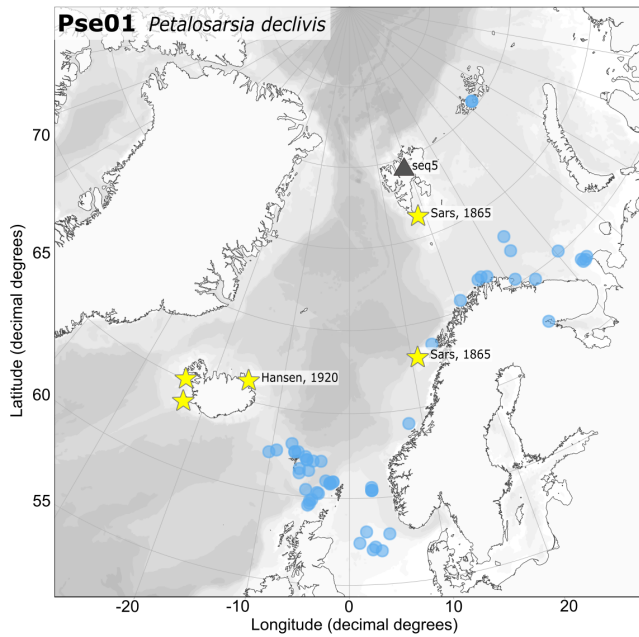

depth [m]

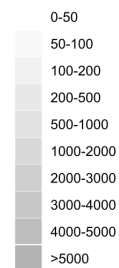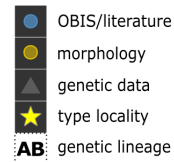

Supplement: Supplemental Information 7 — Occurrence records are shown from the MAREANO and OBIS platform as well as literature data (blue), specimens morphologically investigated in this study (orange) and subsequently genetically investigated (grey triangle with sequence ID) and type and/or syntype locality of putative species (yellow star with reference literature). [file peerj-09-12379-s007.pdf]
