# Supplementary material for: Adding pieces to the puzzle: insights into diversity and distribution patterns of Cumacea (Crustacea: Peracarida) from the deep North Atlantic to the Arctic Ocean
Source: PeerJ. 2021 Nov 11;9:e12379. doi: 10.7717/peerj.12379 (PMC8590803; doi:10.7717/peerj.12379)
Supplement: Supplemental Information 8 — Salinity and temperature ranges for water mass identifications according to (1) Schlichtholz & Houssais (2002) and (2) Hansen & Østerhus (2000) mentioned in this study and used as baseline definitions for a T-S-plot of PASCAL and IceAGE expedition CTD-data. [file peerj-09-12379-s008.pdf]

Supplemental Table S1 Definition of water masses. Salinity and temperature ranges for water mass identifications according to (1) Schlichtholz & Houssais (2002) and (2) Hansen & Østerhus (2000) mentioned in this study and used as baseline definitions for a T-S-plot of PASCAL and IceAGE expedition CTD-data.

Abbreviation	Water mass	Sal.min [ppt]	Sal.max [ppt]	Temp.min [°C]	Temp.max [°C]	Reference
NSDW	Norwegian Sea Deep Water	-Inf	34.4	-Inf	-0.5	2
AIW	Arctic Intermediate Water	34.7	34.9	-1.1	-0.5	1
NSDWc	cold Norwegian Sea Deep Water	34.9	34.92	-1.1	-0.8	1, 2
NSDWw	warm Norwegian Sea Deep Water	34.9	34.92	-0.8	-0.5	1, 2
UPDW	Upper Polar Deep Water	34.7	34.9	-0.5	0	1
NSAIW	Norwegian Sea Arctic Intermediate Water	34.85	34.9	-0.5	0.5	2
APW	Arctic Polar Water	34.3	34.9	0	2	2
ISOW	Iceland Sea Overflow Water	34.85	35	2	3	2
LSW	Labrador Sea Water	34.9	34.95	3	4	2
MNAW	Modified North Atlantic Waters	35.1	35.3	7	8.5	2
NAW	North Atlantic Water	34.91	Inf	2	Inf	1
