# Supplementary material for: Adding pieces to the puzzle: insights into diversity and distribution patterns of Cumacea (Crustacea: Peracarida) from the deep North Atlantic to the Arctic Ocean
Source: PeerJ. 2021 Nov 11;9:e12379. doi: 10.7717/peerj.12379 (PMC8590803; doi:10.7717/peerj.12379)
Supplement: Supplemental Information 9 [file peerj-09-12379-s009.pdf]

Classification	Putative species	Species ID
Family **BODOTRIIDAE** T. Scott, 1901		
	Bodotriidae sp. 1	Bod03
	Bodotriidae sp. 2	Bod04
Subfamily **Bodotriinae**		
	Atlantocuma sp.	-
	Cyclaspis longicaudata Sars, 1865	Bod05-A/-B
	Cyclaspis sp.	-
	Iphinoe serrata Norman, 1867	Bod06
Subfamily **Vaunthompsoniinae**		
	Bathycuma cf. *brevirostre* Norman, 1879	Bod01
	Bathycuma sp. 1	Bod02
	Cumopsis fagei Băcescu, 1956	-
Family **CERATOCUMATIDAE** Calman, 1905		
	Cimmerius reticulatus Jones, 1973	Cer01
Family **DIASTYLIDAE** Bate, 1856		
	Diastylis cornuta Boeck, 1864	Dia01
	Diastylis echinata Bate, 1865	Dia02
	Diastylis goodsiri Bell, 1855	Dia03
	Diastylis laevis Norman, 1869	Dia04
	Diastylis lucifera Krøyer, 1837	Dia05
	Diastylis polaris Sars, 1871	Dia06
	Diastylis rathkei Krøyer, 1841	Dia07
	Diastylis sculpta G.O. Sars, 1871	-
	Diastylis spinulosa Heller, 1875	Dia08
	Diastylis tumida Liljeborg, 1855	Dia09
	Diastyloides atlanticus Reyss, 1974	Dia10
	Diastyloides biplicatus G.O. Sars, 1865	Dia11
	Diastyloides serratus G.O. Sars, 1865	Dia12
	Diastyloides sp. 1	Dia13
	Diastylopsis sp.	-
	Leptostylis ampullacea Lilljeborg, 1855	Dia14
	Leptostylis borealis Stappers, 1908	Dia15
	Leptostylis longimana Sars, 1865	Dia16-A/-B
	Leptostylis sp. 1	Dia17
	Leptostylis sp. 2	Dia18
	Makrokylindrus (Makrokylindrus) cf. *spiniventris* Hansen, 1920	Dia19
Family **LAMPROPIDAE** Sars, 1878		
	Alamprops augustinensis Gerken, 2005	Lam01
	Chalarostylis elegans Norman, 1879	Lam02
	Chalarostylis sp. 1	Lam03
	Hemilamprops assimilis Sars, 1883	Lam04
	Hemilamprops cristatus G.O. Sars, 1870	Lam05-A/-B
	Hemilamprops cf. *diversus* Hale, 1946	Lam06
	Hemilamprops pterini Shalla and Bishop, 2007	Lam07
	Hemilamprops roseus Sars, 1883	Lam08
	Hemilamprops sp. 1 (juv.)	Lam09
	Hemilamprops sp. 2	Lam10
	Hemilamprops uniplicatus G.O. Sars, 1872	Lam11
	Mesolamprops denticulatus Norman, 1863	Lam12
	Platysympus typicus Sars, 1870	Lam13
	Platytyphlops semiornatus Fage, 1929	Lam14
Family **LEUCONIDAE** Sars, 1878		
	Eudorella emarginata Krøyer, 1846	Leu01
	Eudorella hirsuta Sars, 1869	Leu02
	Eudorella pusilla Sars, 1871	-
	Eudorella sp. 1	Leu03
	Eudorella truncatula Bate, 1856	Leu04-A/-B/-C
	Leucon (Alytoleucon) pallidus G.O. Sars, 1865	Leu05
	Leucon (Crymoleucon) antarcticus Zimmer, 1907	-
	Leucon (Crymoleucon) intermedius Mühlenhardt-Siegel, 1996	-
	Leucon (Crymoleucon) rossi Rehm & Heard, 2008	-
	Leucon (Crymoleucon) tener Hansen, 1920	Leu06
	Leucon (Leucon) acutirostris G.O. Sars, 1865	Leu07
	Leucon (Leucon) assimilis Sars, 1886	-
	Leucon (Leucon) nathorsti Ohlin, 1901	Leu08
	Leucon (Leucon) aff. *nathorsti* Ohlin, 1901	Leu09
	Leucon (Leucon) nasicoides Krøyer, 1841	Leu10
	Leucon (Leucon) profundus Hansen, 1920	Leu11
	Leucon (Leucon) cf. *robustus* Hansen, 1920	Leu12
	Leucon (Macrauloleucon) siphonatus Calman, 1905	Leu13
	Leucon (Macrauloleucon) spinulosus Hansen, 1920	Leu14
	Leucon sp.	-
	Leucon sp. 1	Leu15
Family **NANNASTACIDAE** Bate, 1866		
	Campylaspides sp. 1	Nan01
	Campylaspis alba Hansen, 1920	Nan02
	Campylaspis costata Sars, 1865	Nan03
	Campylaspis globosa Hansen, 1920	Nan04
	Campylaspis horrida Sars, 1870	Nan05
	Campylaspis intermedia Hansen, 1920	Nan06
	Campylaspis rubicunda Liljeborg, 1855	Nan07
	Campylaspis sp. 1	Nan08
	Campylaspis sp. 2	Nan09
	Campylaspis sulcata Sars, 1870	Nan10
	Campylaspis undata Sars, 1864	Nan11
	Cumella (Cumella) decipiens Jones, 1984	Nan12
	Cumellopsis cf. *puritani* Calman, 1906	Nan13
	Nannastacidae sp. 1	Nan14
	Procampylaspis ommidion Jones, 1984	Nan15
	Procampylaspis sp. 1	Nan16
	Styloptocuma erectum Jones, 1984	Nan17
	Styloptocuma gracillimum Calman, 1905	Nan18
	Styloptocuma sp. 1	Nan19
	Styloptocuma sp. 2	Nan20
Family **PSEUDOCUMATIDAE** Sars, 1878		
	Petalosarsia declivis Sars, 1865	Pse01
	Pseudocuma sp. 1	Pse02
