# Supplementary material for: Adding pieces to the puzzle: insights into diversity and distribution patterns of Cumacea (Crustacea: Peracarida) from the deep North Atlantic to the Arctic Ocean
Source: PeerJ. 2021 Nov 11;9:e12379. doi: 10.7717/peerj.12379 (PMC8590803; doi:10.7717/peerj.12379)
Supplement: Supplemental Information 10 [file peerj-09-12379-s010.pdf]

Supplemental Table xx Data source and station information on specimens incorporated in the distribution maps.

Data source	Station	Ecoregion	Taxon 1	Taxon 2	decLong	decLat	minDepth	Specimen count
BIOFAR	6	4	Pseudocumatidae	Petalosarsia declivis	-5.3517	62.1522	231	1
BIOFAR	6	4	Lampropidae	Hemilamprops assimilis	-5.3517	62.1522	231	1
BIOFAR	6	4	Lampropidae	Hemilamprops uniplicatus	-5.3517	62.1522	231	1
BIOFAR	6	4	Nannastacidae	Campylaspis globosa	-5.3517	62.1522	231	1
BIOFAR	6	4	Nannastacidae	Campylaspis horrida	-5.3517	62.1522	231	1
BIOFAR	6	4	Nannastacidae	Campylaspis rubicunda	-5.3517	62.1522	231	1
BIOFAR	6	4	Diastylidae	Diastylis cornuta	-5.3517	62.1522	231	1
BIOFAR	6	4	Diastylidae	Diastylis echinata	-5.3517	62.1522	231	1
BIOFAR	6	4	Diastylidae	Diastylis goodsiri	-5.3517	62.1522	231	1
BIOFAR	6	4	Diastylidae	Diastylis lucifera	-5.3517	62.1522	231	1
BIOFAR	6	4	Diastylidae	Diastylis tumida	-5.3517	62.1522	231	1
BIOFAR	7	4	Pseudocumatidae	Petalosarsia declivis	-5.3800	62.1600	218	1
BIOFAR	7	4	Lampropidae	Hemilamprops assimilis	-5.3800	62.1600	218	1
BIOFAR	7	4	Lampropidae	Hemilamprops uniplicatus	-5.3800	62.1600	218	1
BIOFAR	7	4	Nannastacidae	Campylaspis horrida	-5.3800	62.1600	218	1
BIOFAR	7	4	Nannastacidae	Campylaspis rubicunda	-5.3800	62.1600	218	1
BIOFAR	7	4	Diastylidae	Diastylis tumida	-5.3800	62.1600	218	1
BIOFAR	10	4	Lampropidae	Hemilamprops cristatus	-5.0230	62.3140	430	1
BIOFAR	10	4	Leuconidae	Leucon (Crymoleucon) noerrevangi	-5.0230	62.3140	430	1
BIOFAR	15	4	Lampropidae	Hemilamprops cristatus	-4.4037	62.3768	683	1
BIOFAR	15	4	Nannastacidae	Campylaspis affinis	-4.4037	62.3768	683	1
BIOFAR	15	4	Nannastacidae	Campylaspis globosa	-4.4037	62.3768	683	1
BIOFAR	15	4	Leuconidae	Eudorella truncatula	-4.4037	62.3768	683	1
BIOFAR	15	4	Leuconidae	Leucon (Alytoleucon) pallidus	-4.4037	62.3768	683	1
BIOFAR	15	4	Leuconidae	Leucon (Leucon) nathorsti	-4.4037	62.3768	683	1
BIOFAR	15	4	Leuconidae	Leucon (Leucon) serratus	-4.4037	62.3768	683	1
BIOFAR	15	4	Diastylidae	Diastylis echinata	-4.4037	62.3768	683	1
BIOFAR	15	4	Diastylidae	Diastylis glabra	-4.4037	62.3768	683	1
BIOFAR	15	4	Diastylidae	Diastylis polaris	-4.4037	62.3768	683	1
BIOFAR	15	4	Diastylidae	Diastylis scorpioides	-4.4037	62.3768	683	1
BIOFAR	15	4	Diastylidae	Leptostylis longimana	-4.4037	62.3768	683	1
BIOFAR	15	4	Diastylidae	Leptostylis macrura	-4.4037	62.3768	683	1
BIOFAR	15	4	Diastylidae	Leptostylis villosa	-4.4037	62.3768	683	1
BIOFAR	17	4	Lampropidae	Hemilamprops uniplicatus	-4.2451	62.1016	280	1
BIOFAR	19	4	Lampropidae	Hemilamprops assimilis	-4.2500	62.1200	276	1
BIOFAR	19	4	Lampropidae	Hemilamprops cristatus	-4.2500	62.1200	276	1
BIOFAR	19	4	Lampropidae	Hemilamprops uniplicatus	-4.2500	62.1200	276	1
BIOFAR	19	4	Lampropidae	Platysympus tricarinatus	-4.2500	62.1200	276	1
BIOFAR	19	4	Lampropidae	Platysympus typicus	-4.2500	62.1200	276	1
BIOFAR	19	4	Bodotriidae	Cyclaspis longicaudata	-4.2500	62.1200	276	1
BIOFAR	19	4	Nannastacidae	Campylaspis affinis	-4.2500	62.1200	276	1
BIOFAR	19	4	Nannastacidae	Campylaspis globosa	-4.2500	62.1200	276	1
BIOFAR	19	4	Nannastacidae	Campylaspis horrida	-4.2500	62.1200	276	1
BIOFAR	19	4	Nannastacidae	Campylaspis rubicunda	-4.2500	62.1200	276	1
BIOFAR	19	4	Nannastacidae	Campylaspis sulcata	-4.2500	62.1200	276	1
BIOFAR	19	4	Nannastacidae	Campylaspis undata	-4.2500	62.1200	276	1
BIOFAR	19	4	Nannastacidae	Cumellopsis helgae	-4.2500	62.1200	276	1
BIOFAR	19	4	Diastylidae	Diastylis echinata	-4.2500	62.1200	276	1
BIOFAR	19	4	Diastylidae	Diastylis glabra	-4.2500	62.1200	276	1
BIOFAR	27	4	Pseudocumatidae	Petalosarsia declivis	-5.0380	61.5410	225	1
BIOFAR	27	4	Lampropidae	Hemilamprops assimilis	-5.0380	61.5410	225	1
BIOFAR	27	4	Lampropidae	Hemilamprops cristatus	-5.0380	61.5410	225	1
BIOFAR	27	4	Lampropidae	Hemilamprops uniplicatus	-5.0380	61.5410	225	1
BIOFAR	27	4	Lampropidae	Platysympus tricarinatus	-5.0380	61.5410	225	1
BIOFAR	27	4	Bodotriidae	Cyclaspis longicaudata	-5.0380	61.5410	225	1
BIOFAR	27	4	Nannastacidae	Campylaspis affinis	-5.0380	61.5410	225	1
BIOFAR	27	4	Nannastacidae	Campylaspis globosa	-5.0380	61.5410	225	1
BIOFAR	27	4	Nannastacidae	Campylaspis horrida	-5.0380	61.5410	225	1
BIOFAR	27	4	Nannastacidae	Campylaspis intermedia	-5.0380	61.5410	225	1
BIOFAR	27	4	Nannastacidae	Campylaspis laticarpa	-5.0380	61.5410	225	1
BIOFAR	27	4	Nannastacidae	Campylaspis rubicunda	-5.0380	61.5410	225	1
BIOFAR	27	4	Nannastacidae	Campylaspis serratipes	-5.0380	61.5410	225	1
BIOFAR	27	4	Nannastacidae	Campylaspis undata	-5.0380	61.5410	225	1
BIOFAR	27	4	Nannastacidae	Cumella decipiens	-5.0380	61.5410	225	1
BIOFAR	27	4	Bodotriidae	Styloptocuma gracillimum	-5.0380	61.5410	225	1
BIOFAR	27	4	Nannastacidae	Cumellopsis helgae	-5.0380	61.5410	225	1
BIOFAR	27	4	Diastylidae	Diastylis cornuta	-5.0380	61.5410	225	1
BIOFAR	27	4	Diastylidae	Diastylis echinata	-5.0380	61.5410	225	1
BIOFAR	27	4	Diastylidae	Diastyloides scaber	-5.0380	61.5410	225	1
BIOFAR	27	4	Diastylidae	Leptostylis longimana	-5.0380	61.5410	225	1
BIOFAR	27	4	Diastylidae	Leptostylis villosa	-5.0380	61.5410	225	1
BIOFAR	28	4	Bodotriidae	Cyclaspis longicaudata	-5.0735	61.5369	218	1
BIOFAR	28	4	Nannastacidae	Campylaspis globosa	-5.0735	61.5369	218	1
BIOFAR	28	4	Nannastacidae	Campylaspis rubicunda	-5.0735	61.5369	218	1
BIOFAR	28	4	Nannastacidae	Cumellopsis helgae	-5.0735	61.5369	218	1
BIOFAR	29	4	Lampropidae	Hemilamprops assimilis	-5.2500	61.4960	170	1
BIOFAR	29	4	Lampropidae	Hemilamprops uniplicatus	-5.2500	61.4960	170	1
BIOFAR	29	4	Bodotriidae	Cyclaspis longicaudata	-5.2500	61.4960	170	1
BIOFAR	29	4	Nannastacidae	Campylaspis globosa	-5.2500	61.4960	170	1

Supplemental Table xx Data source and station information on specimens incorporated in the distribution maps.

Data source	Station	Ecoregion	Taxon 1	Taxon 2	decLong	decLat	minDepth	Specimen count
BIOFAR	29	4	Nannastacidae	Campylaspis horrida	-5.2500	61.4960	170	1
BIOFAR	29	4	Diastylidae	Diastylis cornuta	-5.2500	61.4960	170	1
BIOFAR	29	4	Diastylidae	Diastylis echinata	-5.2500	61.4960	170	1
BIOFAR	29	4	Diastylidae	Diastyloides scaber	-5.2500	61.4960	170	1
BIOFAR	32	4	Bodotriidae	Cyclaspis longicaudata	-5.4771	61.4175	354	1
BIOFAR	32	4	Nannastacidae	Campylaspis globosa	-5.4771	61.4175	354	1
BIOFAR	32	4	Nannastacidae	Campylaspis horrida	-5.4771	61.4175	354	1
BIOFAR	32	4	Nannastacidae	Campylaspis undata	-5.4771	61.4175	354	1
BIOFAR	32	4	Nannastacidae	Cumella decipiens	-5.4771	61.4175	354	1
BIOFAR	32	4	Bodotriidae	Styloptocuma gracillimum	-5.4771	61.4175	354	1
BIOFAR	32	4	Nannastacidae	Cumellopsis helgae	-5.4771	61.4175	354	1
BIOFAR	32	4	Leuconidae	Eudorella truncatula	-5.4771	61.4175	354	1
BIOFAR	32	4	Diastylidae	Diastylis cornuta	-5.4771	61.4175	354	1
BIOFAR	32	4	Diastylidae	Diastyloides scaber	-5.4771	61.4175	354	1
BIOFAR	33	4	Bodotriidae	Cyclaspis longicaudata	-5.5067	61.4291	351	1
BIOFAR	41	4	Lampropidae	Hemilamprops cristatus	-4.4650	61.1330	780	1
BIOFAR	41	4	Nannastacidae	Campylaspis globosa	-4.4650	61.1330	780	1
BIOFAR	41	4	Leuconidae	Eudorella truncatula	-4.4650	61.1330	780	1
BIOFAR	41	4	Leuconidae	Leucon (Alytoleucon) pallidus	-4.4650	61.1330	780	1
BIOFAR	41	4	Leuconidae	Leucon (Leucon) serratus	-4.4650	61.1330	780	1
BIOFAR	41	4	Diastylidae	Diastylis echinata	-4.4650	61.1330	780	1
BIOFAR	41	4	Diastylidae	Diastylis scorpioides	-4.4650	61.1330	780	1
BIOFAR	41	4	Diastylidae	Leptostylis villosa	-4.4650	61.1330	780	1
BIOFAR	51	4	Lampropidae	Hemilamprops assimilis	-6.1090	61.2430	235	1
BIOFAR	51	4	Lampropidae	Hemilamprops uniplicatus	-6.1090	61.2430	235	1
BIOFAR	51	4	Lampropidae	Platysympus tricarinatus	-6.1090	61.2430	235	1
BIOFAR	51	4	Bodotriidae	Cyclaspis longicaudata	-6.1090	61.2430	235	1
BIOFAR	51	4	Nannastacidae	Campylaspis globosa	-6.1090	61.2430	235	1
BIOFAR	51	4	Nannastacidae	Campylaspis horrida	-6.1090	61.2430	235	1
BIOFAR	51	4	Nannastacidae	Campylaspis intermedia	-6.1090	61.2430	235	1
BIOFAR	51	4	Nannastacidae	Campylaspis rubicunda	-6.1090	61.2430	235	1
BIOFAR	51	4	Nannastacidae	Campylaspis undata	-6.1090	61.2430	235	1
BIOFAR	51	4	Nannastacidae	Cumellopsis helgae	-6.1090	61.2430	235	1
BIOFAR	51	4	Diastylidae	Diastylis echinata	-6.1090	61.2430	235	1
BIOFAR	51	4	Diastylidae	Diastyloides scaber	-6.1090	61.2430	235	1
BIOFAR	56	4	Diastylidae	Diastylis jonesi	-6.2884	61.5478	77	1
BIOFAR	60	4	Nannastacidae	Campylaspis undata	-6.4500	62.0400	68	1
BIOFAR	65	4	Lampropidae	Hemilamprops assimilis	-8.0540	61.3500	322	1
BIOFAR	65	4	Lampropidae	Hemilamprops cristatus	-8.0540	61.3500	322	1
BIOFAR	65	4	Lampropidae	Hemilamprops uniplicatus	-8.0540	61.3500	322	1
BIOFAR	65	4	Lampropidae	Platysympus tricarinatus	-8.0540	61.3500	322	1
BIOFAR	65	4	Bodotriidae	Cyclaspis longicaudata	-8.0540	61.3500	322	1
BIOFAR	65	4	Nannastacidae	Campylaspis globosa	-8.0540	61.3500	322	1
BIOFAR	65	4	Nannastacidae	Campylaspis horrida	-8.0540	61.3500	322	1
BIOFAR	65	4	Nannastacidae	Campylaspis intermedia	-8.0540	61.3500	322	1
BIOFAR	65	4	Nannastacidae	Campylaspis laticarpa	-8.0540	61.3500	322	1
BIOFAR	65	4	Nannastacidae	Campylaspis rubicunda	-8.0540	61.3500	322	1
BIOFAR	65	4	Bodotriidae	Styloptocuma gracillimum	-8.0540	61.3500	322	1
BIOFAR	65	4	Nannastacidae	Cumellopsis helgae	-8.0540	61.3500	322	1
BIOFAR	65	4	Diastylidae	Diastylis cornuta	-8.0540	61.3500	322	1
BIOFAR	65	4	Diastylidae	Diastylis echinata	-8.0540	61.3500	322	1
BIOFAR	65	4	Diastylidae	Diastyloides scaber	-8.0540	61.3500	322	1
BIOFAR	65	4	Diastylidae	Leptostylis macrura	-8.0540	61.3500	322	1
BIOFAR	65	4	Diastylidae	Leptostylis villosa	-8.0540	61.3500	322	1
BIOFAR	68	4	Lampropidae	Hemilamprops cristatus	-9.2040	61.2630	600	1
BIOFAR	68	4	Lampropidae	Paralamprops orbicularis	-9.2040	61.2630	600	1
BIOFAR	68	4	Bodotriidae	Cyclaspis longicaudata	-9.2040	61.2630	600	1
BIOFAR	68	4	Nannastacidae	Campylaspis globosa	-9.2040	61.2630	600	1
BIOFAR	68	4	Nannastacidae	Campylaspis horrida	-9.2040	61.2630	600	1
BIOFAR	68	4	Nannastacidae	Campylaspis sulcata	-9.2040	61.2630	600	1
BIOFAR	68	4	Nannastacidae	Campylaspis undata	-9.2040	61.2630	600	1
BIOFAR	68	4	Nannastacidae	Cumellopsis helgae	-9.2040	61.2630	600	1
BIOFAR	68	4	Diastylidae	Diastylis cornuta	-9.2040	61.2630	600	1
BIOFAR	68	4	Diastylidae	Diastylis echinata	-9.2040	61.2630	600	1
BIOFAR	68	4	Diastylidae	Leptostylis macrura	-9.2040	61.2630	600	1
BIOFAR	70	4	Nannastacidae	Campylaspis sulcata	-8.4397	61.2469	352	1
BIOFAR	70	4	Diastylidae	Diastylis tumida	-8.4397	61.2469	352	1
BIOFAR	73	4	Lampropidae	Hemilamprops assimilis	-8.2950	61.1430	185	1
BIOFAR	73	4	Lampropidae	Hemilamprops uniplicatus	-8.2950	61.1430	185	1
BIOFAR	73	4	Nannastacidae	Campylaspis rubicunda	-8.2950	61.1430	185	1
BIOFAR	75	4	Lampropidae	Hemilamprops uniplicatus	-8.2547	61.1327	156	1
BIOFAR	78	4	Lampropidae	Hemilamprops cristatus	-8.2916	60.5351	150	1
BIOFAR	78	4	Lampropidae	Hemilamprops uniplicatus	-8.2916	60.5351	150	1
BIOFAR	78	4	Nannastacidae	Cumellopsis helgae	-8.2916	60.5351	150	1
BIOFAR	80	4	Nannastacidae	Campylaspis horrida	-8.2793	60.3889	678	1
BIOFAR	82	4	Lampropidae	Hemilamprops cristatus	-8.2507	60.3134	732	1
BIOFAR	82	4	Lampropidae	Hemilamprops uniplicatus	-8.2507	60.3134	732	1
BIOFAR	82	4	Lampropidae	Platysympus tricarinatus	-8.2507	60.3134	732	1
BIOFAR	82	4	Nannastacidae	Campylaspis affinis	-8.2507	60.3134	732	1

Supplemental Table xx Data source and station information on specimens incorporated in the distribution maps.

Data source	Station	Ecoregion	Taxon 1	Taxon 2	decLong	decLat	minDepth	Specimen count
BIOFAR	82	4	Nannastacidae	Campylaspis horrida	-8.2507	60.3134	732	1
BIOFAR	82	4	Nannastacidae	Campylaspis serratipes	-8.2507	60.3134	732	1
BIOFAR	82	4	Leuconidae	Eudorella truncatula	-8.2507	60.3134	732	1
BIOFAR	82	4	Leuconidae	Leucon (Crymoleucon) noerrevangi	-8.2507	60.3134	732	1
BIOFAR	82	4	Diastylidae	Diastylis echinata	-8.2507	60.3134	732	1
BIOFAR	82	4	Diastylidae	Diastylis polaris	-8.2507	60.3134	732	1
BIOFAR	82	4	Diastylidae	Diastylis scorpioides	-8.2507	60.3134	732	1
BIOFAR	95	4	Pseudocumatidae	Petalosarsia declivis	-5.1863	60.4151	803	1
BIOFAR	95	4	Lampropidae	Hemilamprops cristatus	-5.1863	60.4151	803	1
BIOFAR	95	4	Lampropidae	Hemilamprops uniplicatus	-5.1863	60.4151	803	1
BIOFAR	95	4	Nannastacidae	Campylaspis intermedia	-5.1863	60.4151	803	1
BIOFAR	95	4	Leuconidae	Eudorella pusilla	-5.1863	60.4151	803	1
BIOFAR	95	4	Leuconidae	Leucon (Alytoleucon) pallidus	-5.1863	60.4151	803	1
BIOFAR	95	4	Leuconidae	Leucon (Crymoleucon) noerrevangi	-5.1863	60.4151	803	1
BIOFAR	95	4	Leuconidae	Leucon (Leucon) serratus	-5.1863	60.4151	803	1
BIOFAR	95	4	Diastylidae	Diastylis echinata	-5.1863	60.4151	803	1
BIOFAR	95	4	Diastylidae	Diastylis polaris	-5.1863	60.4151	803	1
BIOFAR	95	4	Diastylidae	Leptostylis longimana	-5.1863	60.4151	803	1
BIOFAR	95	4	Diastylidae	Leptostylis macrura	-5.1863	60.4151	803	1
BIOFAR	98	4	Lampropidae	Hemilamprops uniplicatus	-6.1469	60.5442	150	1
BIOFAR	100	4	Pseudocumatidae	Petalosarsia declivis	-6.1706	61.3476	283	1
BIOFAR	100	4	Lampropidae	Hemilamprops assimilis	-6.1706	61.3476	283	1
BIOFAR	100	4	Lampropidae	Hemilamprops uniplicatus	-6.1706	61.3476	283	1
BIOFAR	100	4	Nannastacidae	Campylaspis horrida	-6.1706	61.3476	283	1
BIOFAR	100	4	Nannastacidae	Campylaspis laticarpa	-6.1706	61.3476	283	1
BIOFAR	100	4	Nannastacidae	Campylaspis rubicunda	-6.1706	61.3476	283	1
BIOFAR	100	4	Leuconidae	Eudorella truncatula	-6.1706	61.3476	283	1
BIOFAR	100	4	Diastylidae	Diastylis cornuta	-6.1706	61.3476	283	1
BIOFAR	100	4	Diastylidae	Diastylis echinata	-6.1706	61.3476	283	1
BIOFAR	100	4	Diastylidae	Diastyloides scaber	-6.1706	61.3476	283	1
BIOFAR	100	4	Diastylidae	Leptostylis villosa	-6.1706	61.3476	283	1
BIOFAR	103	4	Lampropidae	Hemilamprops assimilis	-6.4550	62.0560	32	1
BIOFAR	110	4	Diastylidae	Diastylis echinata	-6.3137	62.1467	32	1
BIOFAR	110	4	Diastylidae	Diastylis jonesi	-6.3137	62.1467	32	1
BIOFAR	115	4	Nannastacidae	Campylaspis globosa	-8.4243	61.5213	422	1
BIOFAR	115	4	Nannastacidae	Campylaspis undata	-8.4243	61.5213	422	1
BIOFAR	120	4	Lampropidae	Hemilamprops assimilis	-9.1486	62.1202	515	1
BIOFAR	120	4	Lampropidae	Hemilamprops cristatus	-9.1486	62.1202	515	1
BIOFAR	120	4	Lampropidae	Hemilamprops uniplicatus	-9.1486	62.1202	515	1
BIOFAR	120	4	Nannastacidae	Campylaspis horrida	-9.1486	62.1202	515	1
BIOFAR	120	4	Nannastacidae	Campylaspis undata	-9.1486	62.1202	515	1
BIOFAR	124	4	Lampropidae	Hemilamprops assimilis	-9.3893	62.1694	600	1
BIOFAR	124	4	Lampropidae	Hemilamprops cristatus	-9.3893	62.1694	600	1
BIOFAR	124	4	Nannastacidae	Campylaspis horrida	-9.3893	62.1694	600	1
BIOFAR	124	4	Nannastacidae	Campylaspis rubicunda	-9.3893	62.1694	600	1
BIOFAR	124	4	Nannastacidae	Campylaspis sulcata	-9.3893	62.1694	600	1
BIOFAR	124	4	Nannastacidae	Campylaspis undata	-9.3893	62.1694	600	1
BIOFAR	124	4	Leuconidae	Leucon (Leucon) robustus	-9.3893	62.1694	600	1
BIOFAR	124	4	Leuconidae	Leucon (Leucon) serratus	-9.3893	62.1694	600	1
BIOFAR	127	4	Leuconidae	Eudorella intermedia	-6.4400	62.0800	58	1
BIOFAR	137	4	Lampropidae	Hemilamprops cristatus	-7.1185	61.0251	542	1
BIOFAR	137	4	Lampropidae	Hemilamprops uniplicatus	-7.1185	61.0251	542	1
BIOFAR	137	4	Nannastacidae	Campylaspis globosa	-7.1185	61.0251	542	1
BIOFAR	137	4	Nannastacidae	Campylaspis horrida	-7.1185	61.0251	542	1
BIOFAR	137	4	Nannastacidae	Campylaspis verrucosa	-7.1185	61.0251	542	1
BIOFAR	137	4	Leuconidae	Leucon (Crymoleucon) noerrevangi	-7.1185	61.0251	542	1
BIOFAR	158	4	Lampropidae	Hemilamprops cristatus	-5.3820	61.3840	322	1
BIOFAR	158	4	Bodotriidae	Cyclaspis longicaudata	-5.3820	61.3840	322	1
BIOFAR	158	4	Nannastacidae	Campylaspis caperata	-5.3820	61.3840	322	1
BIOFAR	158	4	Nannastacidae	Campylaspis globosa	-5.3820	61.3840	322	1
BIOFAR	158	4	Nannastacidae	Campylaspis horrida	-5.3820	61.3840	322	1
BIOFAR	158	4	Nannastacidae	Campylaspis undata	-5.3820	61.3840	322	1
BIOFAR	158	4	Nannastacidae	Campylaspis verrucosa	-5.3820	61.3840	322	1
BIOFAR	158	4	Nannastacidae	Cumella angustata	-5.3820	61.3840	322	1
BIOFAR	158	4	Bodotriidae	Styloptocuma gracillimum	-5.3820	61.3840	322	1
BIOFAR	158	4	Nannastacidae	Cumellopsis helgae	-5.3820	61.3840	322	1
BIOFAR	158	4	Diastylidae	Diastylis cornuta	-5.3820	61.3840	322	1
BIOFAR	158	4	Diastylidae	Diastylis echinata	-5.3820	61.3840	322	1
BIOFAR	158	4	Diastylidae	Diastyloides scaber	-5.3820	61.3840	322	1
BIOFAR	165	4	Pseudocumatidae	Petalosarsia declivis	-4.5800	62.1104	184	1
BIOFAR	165	4	Lampropidae	Hemilamprops assimilis	-4.5800	62.1104	184	1
BIOFAR	165	4	Lampropidae	Hemilamprops uniplicatus	-4.5800	62.1104	184	1
BIOFAR	165	4	Nannastacidae	Campylaspis horrida	-4.5800	62.1104	184	1
BIOFAR	165	4	Nannastacidae	Campylaspis rubicunda	-4.5800	62.1104	184	1
BIOFAR	165	4	Nannastacidae	Campylaspis sulcata	-4.5800	62.1104	184	1
BIOFAR	165	4	Nannastacidae	Campylaspis undata	-4.5800	62.1104	184	1
BIOFAR	165	4	Diastylidae	Diastylis echinata	-4.5800	62.1104	184	1
BIOFAR	165	4	Diastylidae	Diastyloides scaber	-4.5800	62.1104	184	1
BIOFAR	165	4	Diastylidae	Leptostylis longimana	-4.5800	62.1104	184	1

Supplemental Table xx Data source and station information on specimens incorporated in the distribution maps.

Data source	Station	Ecoregion	Taxon 1	Taxon 2	decLong	decLat	minDepth	Specimen count
BIOFAR	167	4	Nannastacidae	Campylaspis affinis	-3.3120	62.4680	1032	1
BIOFAR	167	4	Nannastacidae	Campylaspis rubicunda	-3.3120	62.4680	1032	1
BIOFAR	167	4	Leuconidae	Eudorella truncatula	-3.3120	62.4680	1032	1
BIOFAR	167	4	Leuconidae	Leucon (Alytoleucon) pallidus	-3.3120	62.4680	1032	1
BIOFAR	167	4	Diastylidae	Diastylis glabra	-3.3120	62.4680	1032	1
BIOFAR	167	4	Diastylidae	Diastylis polaris	-3.3120	62.4680	1032	1
BIOFAR	167	4	Diastylidae	Leptostylis longimana	-3.3120	62.4680	1032	1
BIOFAR	167	4	Diastylidae	Leptostylis macrura	-3.3120	62.4680	1032	1
BIOFAR	168	4	Leuconidae	Eudorella intermedia	-3.3724	62.4172	899	1
BIOFAR	168	4	Leuconidae	Eudorella truncatula	-3.3724	62.4172	899	1
BIOFAR	168	4	Leuconidae	Leucon (Alytoleucon) pallidus	-3.3724	62.4172	899	1
BIOFAR	168	4	Leuconidae	Leucon (Macrauloleucon) siphonatus	-3.3724	62.4172	899	1
BIOFAR	168	4	Diastylidae	Diastylis glabra	-3.3724	62.4172	899	1
BIOFAR	168	4	Diastylidae	Diastylis polaris	-3.3724	62.4172	899	1
BIOFAR	168	4	Diastylidae	Leptostylis longimana	-3.3724	62.4172	899	1
BIOFAR	168	4	Diastylidae	Leptostylis macrura	-3.3724	62.4172	899	1
BIOFAR	168	4	Diastylidae	Leptostylis villosa	-3.3724	62.4172	899	1
BIOFAR	169	4	Lampropidae	Hemilamprops cristatus	-3.3244	62.3729	808	1
BIOFAR	169	4	Nannastacidae	Campylaspis rubicunda	-3.3244	62.3729	808	1
BIOFAR	169	4	Leuconidae	Eudorella truncatula	-3.3244	62.3729	808	1
BIOFAR	169	4	Leuconidae	Leucon (Alytoleucon) pallidus	-3.3244	62.3729	808	1
BIOFAR	169	4	Diastylidae	Diastylis glabra	-3.3244	62.3729	808	1
BIOFAR	169	4	Diastylidae	Leptostylis longimana	-3.3244	62.3729	808	1
BIOFAR	169	4	Diastylidae	Leptostylis macrura	-3.3244	62.3729	808	1
BIOFAR	170	4	Lampropidae	Hemilamprops cristatus	-3.3108	62.3186	699	1
BIOFAR	170	4	Leuconidae	Eudorella truncatula	-3.3108	62.3186	699	1
BIOFAR	170	4	Leuconidae	Leucon (Alytoleucon) pallidus	-3.3108	62.3186	699	1
BIOFAR	170	4	Diastylidae	Diastylis glabra	-3.3108	62.3186	699	1
BIOFAR	170	4	Diastylidae	Diastylis polaris	-3.3108	62.3186	699	1
BIOFAR	170	4	Diastylidae	Leptostylis longimana	-3.3108	62.3186	699	1
BIOFAR	170	4	Diastylidae	Leptostylis macrura	-3.3108	62.3186	699	1
BIOFAR	171	4	Lampropidae	Hemilamprops cristatus	-3.3160	62.2545	601	1
BIOFAR	171	4	Leuconidae	Eudorella truncatula	-3.3160	62.2545	601	1
BIOFAR	171	4	Leuconidae	Leucon (Alytoleucon) pallidus	-3.3160	62.2545	601	1
BIOFAR	171	4	Diastylidae	Diastylis glabra	-3.3160	62.2545	601	1
BIOFAR	171	4	Diastylidae	Diastylis polaris	-3.3160	62.2545	601	1
BIOFAR	171	4	Diastylidae	Leptostylis macrura	-3.3160	62.2545	601	1
BIOFAR	172	4	Lampropidae	Hemilamprops cristatus	-3.5479	62.1912	507	1
BIOFAR	172	4	Lampropidae	Paralamprops orbicularis	-3.5479	62.1912	507	1
BIOFAR	172	4	Nannastacidae	Campylaspis globosa	-3.5479	62.1912	507	1
BIOFAR	172	4	Nannastacidae	Campylaspis rubicunda	-3.5479	62.1912	507	1
BIOFAR	172	4	Nannastacidae	Campylaspis serratipes	-3.5479	62.1912	507	1
BIOFAR	172	4	Nannastacidae	Campylaspis verrucosa	-3.5479	62.1912	507	1
BIOFAR	172	4	Leuconidae	Eudorella truncatula	-3.5479	62.1912	507	1
BIOFAR	172	4	Leuconidae	Leucon (Alytoleucon) pallidus	-3.5479	62.1912	507	1
BIOFAR	172	4	Diastylidae	Diastylis echinata	-3.5479	62.1912	507	1
BIOFAR	172	4	Diastylidae	Diastylis lucifera	-3.5479	62.1912	507	1
BIOFAR	172	4	Diastylidae	Diastylis polaris	-3.5479	62.1912	507	1
BIOFAR	172	4	Diastylidae	Leptostylis longimana	-3.5479	62.1912	507	1
BIOFAR	172	4	Diastylidae	Leptostylis macrura	-3.5479	62.1912	507	1
BIOFAR	172	4	Diastylidae	Leptostylis villosa	-3.5479	62.1912	507	1
BIOFAR	174	4	Lampropidae	Hemilamprops assimilis	-4.0992	62.1611	300	1
BIOFAR	174	4	Lampropidae	Hemilamprops cristatus	-4.0992	62.1611	300	1
BIOFAR	174	4	Lampropidae	Hemilamprops uniplicatus	-4.0992	62.1611	300	1
BIOFAR	174	4	Nannastacidae	Campylaspis affinis	-4.0992	62.1611	300	1
BIOFAR	174	4	Nannastacidae	Campylaspis horrida	-4.0992	62.1611	300	1
BIOFAR	174	4	Nannastacidae	Campylaspis intermedia	-4.0992	62.1611	300	1
BIOFAR	174	4	Nannastacidae	Campylaspis sulcata	-4.0992	62.1611	300	1
BIOFAR	188	4	Leuconidae	Eudorella truncatula	-6.3147	62.5827	990	1
BIOFAR	188	4	Leuconidae	Leucon (Alytoleucon) pallidus	-6.3147	62.5827	990	1
BIOFAR	188	4	Diastylidae	Diastylis glabra	-6.3147	62.5827	990	1
BIOFAR	189	4	Lampropidae	Hemilamprops assimilis	-6.3970	62.5082	509	1
BIOFAR	189	4	Lampropidae	Hemilamprops uniplicatus	-6.3970	62.5082	509	1
BIOFAR	189	4	Lampropidae	Platysympus tricarinatus	-6.3970	62.5082	509	1
BIOFAR	189	4	Nannastacidae	Campylaspis affinis	-6.3970	62.5082	509	1
BIOFAR	189	4	Nannastacidae	Campylaspis globosa	-6.3970	62.5082	509	1
BIOFAR	189	4	Nannastacidae	Campylaspis horrida	-6.3970	62.5082	509	1
BIOFAR	192	4	Diastylidae	Leptostylis longimana	-6.5308	62.3064	107	1
BIOFAR	227	4	Leuconidae	Leucon (Leucon) serratus	-4.2967	61.0094	1098	1
BIOFAR	227	4	Diastylidae	Diastylis echinata	-4.2967	61.0094	1098	1
BIOFAR	228	4	Lampropidae	Hemilamprops cristatus	-4.4931	61.0647	870	1
BIOFAR	228	4	Lampropidae	Hemilamprops uniplicatus	-4.4931	61.0647	870	1
BIOFAR	228	4	Nannastacidae	Campylaspis laticarpa	-4.4931	61.0647	870	1
BIOFAR	228	4	Leuconidae	Eudorella truncatula	-4.4931	61.0647	870	1
BIOFAR	228	4	Diastylidae	Diastylis echinata	-4.4931	61.0647	870	1
BIOFAR	228	4	Diastylidae	Diastylis glabra	-4.4931	61.0647	870	1
BIOFAR	228	4	Diastylidae	Leptostylis longimana	-4.4931	61.0647	870	1
BIOFAR	261	4	Nannastacidae	Campylaspis globosa	-9.3547	61.3557	1003	1
BIOFAR	261	4	Nannastacidae	Campylaspis intermedia	-9.3547	61.3557	1003	1

Supplemental Table xx Data source and station information on specimens incorporated in the distribution maps.

Data source	Station	Ecoregion	Taxon 1	Taxon 2	decLong	decLat	minDepth	Specimen count
BIOFAR	261	4	Nannastacidae	Campylaspis serratipes	-9.3547	61.3557	1003	1
BIOFAR	261	4	Nannastacidae	Campylaspis undata	-9.3547	61.3557	1003	1
BIOFAR	261	4	Leuconidae	Leucon (Leucon) nathorsti	-9.3547	61.3557	1003	1
BIOFAR	261	4	Leuconidae	Leucon (Leucon) serratus	-9.3547	61.3557	1003	1
BIOFAR	263	4	Lampropidae	Hemilamprops cristatus	-10.0671	62.0532	859	1
BIOFAR	263	4	Lampropidae	Hemilamprops uniplicatus	-10.0671	62.0532	859	1
BIOFAR	263	4	Lampropidae	Platysympus tricarinatus	-10.0671	62.0532	859	1
BIOFAR	263	4	Nannastacidae	Campylaspis affinis	-10.0671	62.0532	859	1
BIOFAR	263	4	Nannastacidae	Campylaspis globosa	-10.0671	62.0532	859	1
BIOFAR	263	4	Nannastacidae	Campylaspis sulcata	-10.0671	62.0532	859	1
BIOFAR	263	4	Nannastacidae	Campylaspis undata	-10.0671	62.0532	859	1
BIOFAR	263	4	Leuconidae	Leucon (Crymoleucon) noerrevangi	-10.0671	62.0532	859	1
BIOFAR	263	4	Diastylidae	Diastylis echinata	-10.0671	62.0532	859	1
BIOFAR	264	4	Nannastacidae	Campylaspis globosa	-9.5890	62.0840	780	1
BIOFAR	264	4	Nannastacidae	Campylaspis undata	-9.5890	62.0840	780	1
BIOFAR	265	4	Nannastacidae	Campylaspis serratipes	-9.5152	62.1107	684	1
BIOFAR	267	4	Lampropidae	Hemilamprops assimilis	-8.3375	62.4240	498	1
BIOFAR	267	4	Lampropidae	Hemilamprops cristatus	-8.3375	62.4240	498	1
BIOFAR	267	4	Nannastacidae	Campylaspis globosa	-8.3375	62.4240	498	1
BIOFAR	267	4	Nannastacidae	Campylaspis horrida	-8.3375	62.4240	498	1
BIOFAR	267	4	Leuconidae	Leucon (Leucon) robustus	-8.3375	62.4240	498	1
BIOFAR	270	4	Leuconidae	Leucon (Leucon) fulvus	-8.0658	62.5302	570	1
BIOFAR	271	4	Lampropidae	Hemilamprops cristatus	-8.0924	62.5230	559	1
BIOFAR	271	4	Lampropidae	Hemilamprops uniplicatus	-8.0924	62.5230	559	1
BIOFAR	271	4	Nannastacidae	Campylaspis globosa	-8.0924	62.5230	559	1
BIOFAR	271	4	Leuconidae	Eudorella truncatula	-8.0924	62.5230	559	1
BIOFAR	271	4	Leuconidae	Leucon (Alytoleucon) pallidus	-8.0924	62.5230	559	1
BIOFAR	271	4	Leuconidae	Leucon (Leucon) robustus	-8.0924	62.5230	559	1
BIOFAR	271	4	Diastylidae	Diastylis echinata	-8.0924	62.5230	559	1
BIOFAR	271	4	Diastylidae	Diastylis glabra	-8.0924	62.5230	559	1
BIOFAR	271	4	Diastylidae	Diastylis polaris	-8.0924	62.5230	559	1
BIOFAR	271	4	Diastylidae	Leptostylis longimana	-8.0924	62.5230	559	1
BIOFAR	274	4	Pseudocumatidae	Petalosarsia declivis	-7.4922	63.0079	698	1
BIOFAR	274	4	Lampropidae	Hemilamprops cristatus	-7.4922	63.0079	698	1
BIOFAR	274	4	Lampropidae	Hemilamprops uniplicatus	-7.4922	63.0079	698	1
BIOFAR	274	4	Nannastacidae	Campylaspis affinis	-7.4922	63.0079	698	1
BIOFAR	274	4	Leuconidae	Eudorella truncatula	-7.4922	63.0079	698	1
BIOFAR	274	4	Leuconidae	Leucon (Crymoleucon) noerrevangi	-7.4922	63.0079	698	1
BIOFAR	274	4	Leuconidae	Leucon (Epileucon) acutirostris	-7.4922	63.0079	698	1
BIOFAR	274	4	Diastylidae	Diastylis polaris	-7.4922	63.0079	698	1
BIOFAR	274	4	Diastylidae	Leptostylis villosa	-7.4922	63.0079	698	1
BIOFAR	275	4	Lampropidae	Hemilamprops uniplicatus	-7.4158	63.0318	804	1
BIOFAR	275	4	Leuconidae	Eudorella truncatula	-7.4158	63.0318	804	1
BIOFAR	275	4	Diastylidae	Diastylis polaris	-7.4158	63.0318	804	1
BIOFAR	295	4	Lampropidae	Hemilamprops cristatus	-7.3700	59.5440	655	1
BIOFAR	295	4	Lampropidae	Hemilamprops uniplicatus	-7.3700	59.5440	655	1
BIOFAR	295	4	Nannastacidae	Campylaspis sulcata	-7.3700	59.5440	655	1
BIOFAR	295	4	Nannastacidae	Campylaspis undata	-7.3700	59.5440	655	1
BIOFAR	295	4	Leuconidae	Leucon (Leucon) serratus	-7.3700	59.5440	655	1
BIOFAR	295	4	Diastylidae	Makrokylindrus (Adiastylis) josephinae	-7.3700	59.5440	655	1
BIOFAR	295	4	Diastylidae	Makrokylindrus (Adiastylis) longipes	-7.3700	59.5440	655	1
BIOFAR	317	4	Lampropidae	Hemilamprops cristatus	-11.0410	60.3520	650	1
BIOFAR	343	4	Lampropidae	Hemilamprops cristatus	-9.3260	62.1460	594	1
BIOFAR	343	4	Nannastacidae	Campylaspis undata	-9.3260	62.1460	594	1
BIOFAR	355	4	Pseudocumatidae	Petalosarsia declivis	-5.5780	62.3380	149	1
BIOFAR	355	4	Lampropidae	Hemilamprops assimilis	-5.5780	62.3380	149	1
BIOFAR	355	4	Lampropidae	Hemilamprops uniplicatus	-5.5780	62.3380	149	1
BIOFAR	355	4	Diastylidae	Diastylis tumida	-5.5780	62.3380	149	1
BIOFAR	356	4	Lampropidae	Hemilamprops assimilis	-5.1960	62.2540	240	1
BIOFAR	356	4	Lampropidae	Hemilamprops uniplicatus	-5.1960	62.2540	240	1
BIOFAR	356	4	Nannastacidae	Campylaspis horrida	-5.1960	62.2540	240	1
BIOFAR	357	4	Lampropidae	Hemilamprops uniplicatus	-4.4360	62.0410	205	1
BIOFAR	357	4	Bodotriidae	Cyclaspis longicaudata	-4.4360	62.0410	205	1
BIOFAR	357	4	Nannastacidae	Campylaspis globosa	-4.4360	62.0410	205	1
BIOFAR	361	4	Lampropidae	Hemilamprops cristatus	-4.0500	61.5030	700	1
BIOFAR	361	4	Leuconidae	Eudorella truncatula	-4.0500	61.5030	700	1
BIOFAR	361	4	Diastylidae	Diastylis echinata	-4.0500	61.5030	700	1
BIOFAR	361	4	Diastylidae	Diastylis glabra	-4.0500	61.5030	700	1
BIOFAR	361	4	Diastylidae	Diastylis polaris	-4.0500	61.5030	700	1
BIOFAR	361	4	Diastylidae	Diastylis stygia	-4.0500	61.5030	700	1
BIOFAR	362	4	Lampropidae	Hemilamprops cristatus	-3.5120	61.4930	798	1
BIOFAR	380	4	Pseudocumatidae	Petalosarsia declivis	-3.5815	62.1348	425	1
BIOFAR	380	4	Lampropidae	Hemilamprops assimilis	-3.5815	62.1348	425	1
BIOFAR	380	4	Lampropidae	Hemilamprops cristatus	-3.5815	62.1348	425	1
BIOFAR	380	4	Lampropidae	Platysympus tricarinatus	-3.5815	62.1348	425	1
BIOFAR	380	4	Nannastacidae	Campylaspis affinis	-3.5815	62.1348	425	1
BIOFAR	380	4	Nannastacidae	Campylaspis globosa	-3.5815	62.1348	425	1
BIOFAR	380	4	Nannastacidae	Campylaspis horrida	-3.5815	62.1348	425	1
BIOFAR	380	4	Nannastacidae	Campylaspis intermedia	-3.5815	62.1348	425	1

Supplemental Table xx Data source and station information on specimens incorporated in the distribution maps.

Data source	Station	Ecoregion	Taxon 1	Taxon 2	decLong	decLat	minDepth	Specimen count
BIOFAR	380	4	Nannastacidae	Campylaspis rubicunda	-3.5815	62.1348	425	1
BIOFAR	380	4	Nannastacidae	Campylaspis serratipes	-3.5815	62.1348	425	1
BIOFAR	380	4	Nannastacidae	Campylaspis undata	-3.5815	62.1348	425	1
BIOFAR	380	4	Nannastacidae	Campylaspis verrucosa	-3.5815	62.1348	425	1
BIOFAR	380	4	Leuconidae	Leucon (Crymoleucon) noerrevangi	-3.5815	62.1348	425	1
BIOFAR	380	4	Diastylidae	Diastylis echinata	-3.5815	62.1348	425	1
BIOFAR	381	4	Lampropidae	Hemilamprops assimilis	-3.5954	62.1230	402	1
BIOFAR	381	4	Lampropidae	Hemilamprops cristatus	-3.5954	62.1230	402	1
BIOFAR	381	4	Lampropidae	Hemilamprops uniplicatus	-3.5954	62.1230	402	1
BIOFAR	381	4	Lampropidae	Platysympus tricarinatus	-3.5954	62.1230	402	1
BIOFAR	381	4	Nannastacidae	Campylaspis affinis	-3.5954	62.1230	402	1
BIOFAR	381	4	Nannastacidae	Campylaspis globosa	-3.5954	62.1230	402	1
BIOFAR	381	4	Nannastacidae	Campylaspis horrida	-3.5954	62.1230	402	1
BIOFAR	381	4	Nannastacidae	Cumellopsis helgae	-3.5954	62.1230	402	1
BIOFAR	381	4	Leuconidae	Leucon (Crymoleucon) noerrevangi	-3.5954	62.1230	402	1
BIOFAR	381	4	Diastylidae	Diastylis echinata	-3.5954	62.1230	402	1
BIOFAR	382	4	Lampropidae	Hemilamprops uniplicatus	-4.2157	62.1178	281	1
BIOFAR	382	4	Nannastacidae	Campylaspis horrida	-4.2157	62.1178	281	1
BIOFAR	382	4	Nannastacidae	Campylaspis serratipes	-4.2157	62.1178	281	1
BIOFAR	382	4	Nannastacidae	Campylaspis undata	-4.2157	62.1178	281	1
BIOFAR	408	4	Pseudocumatidae	Petalosarsia declivis	-6.2000	61.5970	110	1
BIOFAR	408	4	Leuconidae	Leucon (Leucon) nathorsti	-6.2000	61.5970	110	1
BIOFAR	408	4	Diastylidae	Diastylis tumida	-6.2000	61.5970	110	1
BIOFAR	408	4	Diastylidae	Leptostylis villosa	-6.2000	61.5970	110	1
BIOFAR	409	4	Lampropidae	Hemilamprops uniplicatus	-8.1623	62.2173	129	1
BIOFAR	410	4	Lampropidae	Hemilamprops uniplicatus	-8.3367	62.1996	240	1
BIOFAR	410	4	Diastylidae	Diastylis tumida	-8.3367	62.1996	240	1
BIOFAR	411	4	Lampropidae	Hemilamprops assimilis	-8.4730	62.2385	430	1
BIOFAR	411	4	Lampropidae	Hemilamprops uniplicatus	-8.4730	62.2385	430	1
BIOFAR	411	4	Lampropidae	Platysympus tricarinatus	-8.4730	62.2385	430	1
BIOFAR	411	4	Bodotriidae	Cyclaspis longicaudata	-8.4730	62.2385	430	1
BIOFAR	411	4	Nannastacidae	Campylaspis globosa	-8.4730	62.2385	430	1
BIOFAR	411	4	Nannastacidae	Campylaspis horrida	-8.4730	62.2385	430	1
BIOFAR	411	4	Nannastacidae	Campylaspis serratipes	-8.4730	62.2385	430	1
BIOFAR	411	4	Nannastacidae	Campylaspis undata	-8.4730	62.2385	430	1
BIOFAR	411	4	Nannastacidae	Cumellopsis helgae	-8.4730	62.2385	430	1
BIOFAR	411	4	Leuconidae	Leucon (Crymoleucon) noerrevangi	-8.4730	62.2385	430	1
BIOFAR	416	4	Lampropidae	Hemilamprops assimilis	-11.1110	62.0893	1038	1
BIOFAR	416	4	Lampropidae	Hemilamprops cristatus	-11.1110	62.0893	1038	1
BIOFAR	416	4	Lampropidae	Platysympus tricarinatus	-11.1110	62.0893	1038	1
BIOFAR	416	4	Nannastacidae	Campylaspis affinis	-11.1110	62.0893	1038	1
BIOFAR	416	4	Nannastacidae	Campylaspis globosa	-11.1110	62.0893	1038	1
BIOFAR	416	4	Nannastacidae	Campylaspis horrida	-11.1110	62.0893	1038	1
BIOFAR	416	4	Nannastacidae	Campylaspis intermedia	-11.1110	62.0893	1038	1
BIOFAR	416	4	Nannastacidae	Campylaspis undata	-11.1110	62.0893	1038	1
BIOFAR	416	4	Leuconidae	Leucon (Crymoleucon) noerrevangi	-11.1110	62.0893	1038	1
BIOFAR	416	4	Leuconidae	Leucon (Leucon) nathorsti	-11.1110	62.0893	1038	1
BIOFAR	416	4	Diastylidae	Diastylis echinata	-11.1110	62.0893	1038	1
BIOFAR	416	4	Diastylidae	Leptostylis longimana	-11.1110	62.0893	1038	1
BIOFAR	417	4	Lampropidae	Hemilamprops cristatus	-10.5813	62.1656	894	1
BIOFAR	417	4	Lampropidae	Hemilamprops uniplicatus	-10.5813	62.1656	894	1
BIOFAR	417	4	Lampropidae	Platysympus tricarinatus	-10.5813	62.1656	894	1
BIOFAR	417	4	Nannastacidae	Campylaspis globosa	-10.5813	62.1656	894	1
BIOFAR	417	4	Nannastacidae	Campylaspis undata	-10.5813	62.1656	894	1
BIOFAR	417	4	Leuconidae	Leucon (Crymoleucon) noerrevangi	-10.5813	62.1656	894	1
BIOFAR	421	4	Pseudocumatidae	Petalosarsia declivis	-10.2692	62.3215	597	1
BIOFAR	421	4	Lampropidae	Hemilamprops assimilis	-10.2692	62.3215	597	1
BIOFAR	421	4	Lampropidae	Hemilamprops cristatus	-10.2692	62.3215	597	1
BIOFAR	421	4	Lampropidae	Hemilamprops uniplicatus	-10.2692	62.3215	597	1
BIOFAR	421	4	Lampropidae	Platysympus tricarinatus	-10.2692	62.3215	597	1
BIOFAR	421	4	Nannastacidae	Campylaspis affinis	-10.2692	62.3215	597	1
BIOFAR	421	4	Nannastacidae	Campylaspis globosa	-10.2692	62.3215	597	1
BIOFAR	421	4	Nannastacidae	Campylaspis horrida	-10.2692	62.3215	597	1
BIOFAR	421	4	Nannastacidae	Campylaspis rubicunda	-10.2692	62.3215	597	1
BIOFAR	421	4	Nannastacidae	Campylaspis undata	-10.2692	62.3215	597	1
BIOFAR	421	4	Nannastacidae	Cumellopsis helgae	-10.2692	62.3215	597	1
BIOFAR	421	4	Leuconidae	Leucon (Crymoleucon) noerrevangi	-10.2692	62.3215	597	1
BIOFAR	421	4	Diastylidae	Diastylis echinata	-10.2692	62.3215	597	1
BIOFAR	422	4	Lampropidae	Hemilamprops assimilis	-10.0390	62.4131	500	1
BIOFAR	422	4	Lampropidae	Hemilamprops cristatus	-10.0390	62.4131	500	1
BIOFAR	422	4	Lampropidae	Hemilamprops uniplicatus	-10.0390	62.4131	500	1
BIOFAR	422	4	Nannastacidae	Campylaspis globosa	-10.0390	62.4131	500	1
BIOFAR	422	4	Nannastacidae	Campylaspis horrida	-10.0390	62.4131	500	1
BIOFAR	422	4	Nannastacidae	Campylaspis undata	-10.0390	62.4131	500	1
BIOFAR	422	4	Nannastacidae	Cumellopsis helgae	-10.0390	62.4131	500	1
BIOFAR	422	4	Leuconidae	Leucon (Crymoleucon) noerrevangi	-10.0390	62.4131	500	1
BIOFAR	424	4	Pseudocumatidae	Petalosarsia declivis	-9.3442	62.5024	509	1
BIOFAR	424	4	Lampropidae	Hemilamprops assimilis	-9.3442	62.5024	509	1
BIOFAR	424	4	Lampropidae	Hemilamprops cristatus	-9.3442	62.5024	509	1

Supplemental Table xx Data source and station information on specimens incorporated in the distribution maps.

Data source	Station	Ecoregion	Taxon 1	Taxon 2	decLong	decLat	minDepth	Specimen count
BIOFAR	424	4	Lampropidae	Hemilamprops uniplicatus	-9.3442	62.5024	509	1
BIOFAR	424	4	Lampropidae	Platysympus tricarinatus	-9.3442	62.5024	509	1
BIOFAR	424	4	Nannastacidae	Campylaspis affinis	-9.3442	62.5024	509	1
BIOFAR	424	4	Nannastacidae	Campylaspis globosa	-9.3442	62.5024	509	1
BIOFAR	424	4	Nannastacidae	Campylaspis horrida	-9.3442	62.5024	509	1
BIOFAR	424	4	Nannastacidae	Campylaspis rubicunda	-9.3442	62.5024	509	1
BIOFAR	424	4	Leuconidae	Eudorella truncatula	-9.3442	62.5024	509	1
BIOFAR	424	4	Leuconidae	Leucon (Alytoleucon) pallidus	-9.3442	62.5024	509	1
BIOFAR	424	4	Leuconidae	Leucon (Crymoleucon) noerrevangi	-9.3442	62.5024	509	1
BIOFAR	424	4	Leuconidae	Leucon (Leucon) serratus	-9.3442	62.5024	509	1
BIOFAR	424	4	Diastylidae	Diastylis echinata	-9.3442	62.5024	509	1
BIOFAR	424	4	Diastylidae	Diastylis glabra	-9.3442	62.5024	509	1
BIOFAR	424	4	Diastylidae	Diastylis polaris	-9.3442	62.5024	509	1
BIOFAR	424	4	Diastylidae	Leptostylis villosa	-9.3442	62.5024	509	1
BIOFAR	425	4	Lampropidae	Hemilamprops assimilis	-9.2812	62.5603	509	1
BIOFAR	425	4	Lampropidae	Hemilamprops cristatus	-9.2812	62.5603	509	1
BIOFAR	425	4	Lampropidae	Hemilamprops uniplicatus	-9.2812	62.5603	509	1
BIOFAR	425	4	Nannastacidae	Campylaspis globosa	-9.2812	62.5603	509	1
BIOFAR	425	4	Leuconidae	Eudorella truncatula	-9.2812	62.5603	509	1
BIOFAR	425	4	Diastylidae	Diastylis echinata	-9.2812	62.5603	509	1
BIOFAR	425	4	Diastylidae	Diastylis glabra	-9.2812	62.5603	509	1
BIOFAR	425	4	Diastylidae	Diastylis polaris	-9.2812	62.5603	509	1
BIOFAR	452	4	Lampropidae	Hemilamprops assimilis	-7.1423	62.4583	416	1
BIOFAR	452	4	Lampropidae	Hemilamprops uniplicatus	-7.1423	62.4583	416	1
BIOFAR	452	4	Bodotriidae	Cyclaspis longicaudata	-7.1423	62.4583	416	1
BIOFAR	452	4	Nannastacidae	Campylaspis globosa	-7.1423	62.4583	416	1
BIOFAR	452	4	Nannastacidae	Campylaspis horrida	-7.1423	62.4583	416	1
BIOFAR	452	4	Nannastacidae	Campylaspis undata	-7.1423	62.4583	416	1
BIOFAR	458	4	Pseudocumatidae	Petalosarsia declivis	-7.0023	62.5492	675	1
BIOFAR	458	4	Pseudocumatidae	Petalosarsia declivis	-7.0023	62.5492	675	1
BIOFAR	458	4	Lampropidae	Hemilamprops cristatus	-7.0023	62.5492	675	1
BIOFAR	458	4	Nannastacidae	Campylaspis globosa	-7.0023	62.5492	675	1
BIOFAR	458	4	Leuconidae	Eudorella truncatula	-7.0023	62.5492	675	1
BIOFAR	458	4	Leuconidae	Leucon (Crymoleucon) noerrevangi	-7.0023	62.5492	675	1
BIOFAR	458	4	Diastylidae	Diastylis tumida	-7.0023	62.5492	675	1
BIOFAR	459	4	Lampropidae	Hemilamprops cristatus	-6.5752	62.5942	910	1
BIOFAR	459	4	Lampropidae	Hemilamprops uniplicatus	-6.5752	62.5942	910	1
BIOFAR	459	4	Diastylidae	Diastylis glabra	-6.5752	62.5942	910	1
BIOFAR	459	4	Diastylidae	Leptostylis villosa	-6.5752	62.5942	910	1
BIOFAR	473	4	Lampropidae	Hemilamprops uniplicatus	-5.4300	62.3611	198	1
BIOFAR	478	4	Lampropidae	Hemilamprops cristatus	-4.4392	61.0366	973	1
BIOFAR	478	4	Lampropidae	Hemilamprops uniplicatus	-4.4392	61.0366	973	1
BIOFAR	478	4	Leuconidae	Eudorella truncatula	-4.4392	61.0366	973	1
BIOFAR	478	4	Leuconidae	Leucon (Alytoleucon) pallidus	-4.4392	61.0366	973	1
BIOFAR	478	4	Diastylidae	Leptostylis macrura	-4.4392	61.0366	973	1
BIOFAR	480	4	Lampropidae	Hemilamprops cristatus	-5.0650	60.5637	806	1
BIOFAR	480	4	Leuconidae	Eudorella truncatula	-5.0650	60.5637	806	1
BIOFAR	480	4	Diastylidae	Leptostylis villosa	-5.0650	60.5637	806	1
BIOFAR	482	4	Lampropidae	Hemilamprops uniplicatus	-5.1394	61.0194	509	1
BIOFAR	482	4	Nannastacidae	Campylaspis globosa	-5.1394	61.0194	509	1
BIOFAR	482	4	Nannastacidae	Campylaspis horrida	-5.1394	61.0194	509	1
BIOFAR	482	4	Nannastacidae	Campylaspis rubicunda	-5.1394	61.0194	509	1
BIOFAR	490	4	Ceratocumatidae	Ceratocuma horridum	-9.5584	60.5851	1083	1
BIOFAR	490	4	Nannastacidae	Cumellopsis helgae	-9.5584	60.5851	1083	1
BIOFAR	490	4	Nannastacidae	Procampylaspis bituberculata	-9.5584	60.5851	1083	1
BIOFAR	490	4	Diastylidae	Diastylis echinata	-9.5584	60.5851	1083	1
BIOFAR	490	4	Diastylidae	Diastylis glabra	-9.5584	60.5851	1083	1
BIOFAR	490	4	Diastylidae	Diastylis goodsiri	-9.5584	60.5851	1083	1
BIOFAR	492	4	Lampropidae	Hemilamprops assimilis	-9.5680	60.5260	900	1
BIOFAR	492	4	Bodotriidae	Cyclaspis longicaudata	-9.5680	60.5260	900	1
BIOFAR	492	4	Diastylidae	Diastylis echinata	-9.5680	60.5260	900	1
BIOFAR	493	4	Lampropidae	Hemilamprops assimilis	-9.5327	60.4938	800	1
BIOFAR	493	4	Bodotriidae	Cyclaspis longicaudata	-9.5327	60.4938	800	1
BIOFAR	495	4	Lampropidae	Hemilamprops assimilis	-9.3904	60.3822	584	1
BIOFAR	495	4	Lampropidae	Hemilamprops cristatus	-9.3904	60.3822	584	1
BIOFAR	495	4	Lampropidae	Platysympus tricarinatus	-9.3904	60.3822	584	1
BIOFAR	495	4	Bodotriidae	Cyclaspis longicaudata	-9.3904	60.3822	584	1
BIOFAR	495	4	Nannastacidae	Campylaspis globosa	-9.3904	60.3822	584	1
BIOFAR	495	4	Nannastacidae	Campylaspis laticarpa	-9.3904	60.3822	584	1
BIOFAR	495	4	Nannastacidae	Campylaspis undata	-9.3904	60.3822	584	1
BIOFAR	495	4	Diastylidae	Diastylis echinata	-9.3904	60.3822	584	1
BIOFAR	496	4	Lampropidae	Hemilamprops assimilis	-9.3560	60.3370	515	1
BIOFAR	496	4	Lampropidae	Hemilamprops uniplicatus	-9.3560	60.3370	515	1
BIOFAR	496	4	Nannastacidae	Campylaspis globosa	-9.3560	60.3370	515	1
BIOFAR	496	4	Nannastacidae	Campylaspis laticarpa	-9.3560	60.3370	515	1
BIOFAR	496	4	Nannastacidae	Campylaspis sulcata	-9.3560	60.3370	515	1
BIOFAR	500	4	Lampropidae	Hemilamprops uniplicatus	-8.2264	60.2687	714	1
BIOFAR	500	4	Nannastacidae	Campylaspis globosa	-8.2264	60.2687	714	1
BIOFAR	500	4	Leuconidae	Leucon (Crymoleucon) noerrevangi	-8.2264	60.2687	714	1

Supplemental Table xx Data source and station information on specimens incorporated in the distribution maps.

Data source	Station	Ecoregion	Taxon 1	Taxon 2	decLong	decLat	minDepth	Specimen count
BIOFAR	500	4	Diastylidae	Diastylis polaris	-8.2264	60.2687	714	1
BIOFAR	501	4	Lampropidae	Hemilamprops cristatus	-8.1570	60.3020	804	1
BIOFAR	501	4	Lampropidae	Hemilamprops uniplicatus	-8.1570	60.3020	804	1
BIOFAR	501	4	Leuconidae	Leucon (Crymoleucon) noerrevangi	-8.1570	60.3020	804	1
BIOFAR	501	4	Leuconidae	Leucon (Leucon) serratus	-8.1570	60.3020	804	1
BIOFAR	501	4	Diastylidae	Diastylis echinata	-8.1570	60.3020	804	1
BIOFAR	502	4	Lampropidae	Hemilamprops uniplicatus	-8.0404	60.3026	890	1
BIOFAR	502	4	Leuconidae	Leucon (Crymoleucon) noerrevangi	-8.0404	60.3026	890	1
BIOFAR	506	4	Lampropidae	Hemilamprops uniplicatus	-8.3480	60.4060	350	1
BIOFAR	506	4	Nannastacidae	Campylaspis undata	-8.3480	60.4060	350	1
BIOFAR	515	4	Lampropidae	Hemilamprops assimilis	-11.4650	60.4180	700	1
BIOFAR	515	4	Bodotriidae	Cyclaspis longicaudata	-11.4650	60.4180	700	1
BIOFAR	515	4	Nannastacidae	Campylaspis rubicunda	-11.4650	60.4180	700	1
BIOFAR	515	4	Nannastacidae	Campylaspis undata	-11.4650	60.4180	700	1
BIOFAR	516	4	Ceratocumatidae	Cimmerius reticulatus	-11.4056	60.4005	914	1
BIOFAR	516	4	Nannastacidae	Campylaspis undata	-11.4056	60.4005	914	1
BIOFAR	516	4	Nannastacidae	Cumellopsis helgae	-11.4056	60.4005	914	1
BIOFAR	517	4	Ceratocumatidae	Cimmerius reticulatus	-11.3771	60.3574	1099	1
BIOFAR	517	4	Lampropidae	Hemilamprops assimilis	-11.3771	60.3574	1099	1
BIOFAR	517	4	Lampropidae	Platysympus typicus	-11.3771	60.3574	1099	1
BIOFAR	517	4	Nannastacidae	Cumellopsis helgae	-11.3771	60.3574	1099	1
BIOFAR	517	4	Diastylidae	Diastylis tumida	-11.3771	60.3574	1099	1
BIOFAR	518	4	Lampropidae	Hemilamprops uniplicatus	-11.5545	60.3331	423	1
BIOFAR	518	4	Lampropidae	Paralamprops orbicularis	-11.5545	60.3331	423	1
BIOFAR	518	4	Lampropidae	Platysympus tricarinatus	-11.5545	60.3331	423	1
BIOFAR	518	4	Bodotriidae	Cyclaspis longicaudata	-11.5545	60.3331	423	1
BIOFAR	518	4	Nannastacidae	Campylaspis intermedia	-11.5545	60.3331	423	1
BIOFAR	518	4	Nannastacidae	Campylaspis rubicunda	-11.5545	60.3331	423	1
BIOFAR	518	4	Diastylidae	Diastylis echinata	-11.5545	60.3331	423	1
BIOFAR	518	4	Diastylidae	Diastylis tumida	-11.5545	60.3331	423	1
BIOFAR	519	4	Lampropidae	Hemilamprops uniplicatus	-12.3279	60.2485	303	1
BIOFAR	522	4	Lampropidae	Hemilamprops assimilis	-12.3630	60.3953	514	1
BIOFAR	522	4	Lampropidae	Hemilamprops uniplicatus	-12.3630	60.3953	514	1
BIOFAR	522	4	Lampropidae	Paralamprops orbicularis	-12.3630	60.3953	514	1
BIOFAR	522	4	Lampropidae	Platysympus tricarinatus	-12.3630	60.3953	514	1
BIOFAR	522	4	Bodotriidae	Cyclaspis longicaudata	-12.3630	60.3953	514	1
BIOFAR	522	4	Nannastacidae	Campylaspis intermedia	-12.3630	60.3953	514	1
BIOFAR	522	4	Nannastacidae	Campylaspis rubicunda	-12.3630	60.3953	514	1
BIOFAR	522	4	Diastylidae	Diastylis echinata	-12.3630	60.3953	514	1
BIOFAR	524	4	Lampropidae	Hemilamprops assimilis	-12.3720	60.4411	702	1
BIOFAR	524	4	Bodotriidae	Cyclaspis longicaudata	-12.3720	60.4411	702	1
BIOFAR	524	4	Nannastacidae	Campylaspis rubicunda	-12.3720	60.4411	702	1
BIOFAR	524	4	Nannastacidae	Campylaspis undata	-12.3720	60.4411	702	1
BIOFAR	524	4	Nannastacidae	Cumellopsis helgae	-12.3720	60.4411	702	1
BIOFAR	524	4	Leuconidae	Leucon (Leucon) nathorsti	-12.3720	60.4411	702	1
BIOFAR	689	4	Lampropidae	Hemilamprops assimilis	-12.1896	60.3501	351	1
BIOFAR	689	4	Lampropidae	Hemilamprops uniplicatus	-12.1896	60.3501	351	1
BIOFAR	689	4	Bodotriidae	Cyclaspis longicaudata	-12.1896	60.3501	351	1
BIOFAR	689	4	Nannastacidae	Campylaspis horrida	-12.1896	60.3501	351	1
BIOFAR	689	4	Nannastacidae	Campylaspis rubicunda	-12.1896	60.3501	351	1
BIOFAR	689	4	Diastylidae	Diastylis cornuta	-12.1896	60.3501	351	1
BIOFAR	690	4	Lampropidae	Hemilamprops assimilis	-12.1663	60.3459	357	1
BIOFAR	690	4	Lampropidae	Hemilamprops uniplicatus	-12.1663	60.3459	357	1
BIOFAR	690	4	Lampropidae	Platysympus tricarinatus	-12.1663	60.3459	357	1
BIOFAR	690	4	Bodotriidae	Cyclaspis longicaudata	-12.1663	60.3459	357	1
BIOFAR	690	4	Nannastacidae	Campylaspis affinis	-12.1663	60.3459	357	1
BIOFAR	690	4	Nannastacidae	Campylaspis horridoides	-12.1663	60.3459	357	1
BIOFAR	690	4	Nannastacidae	Campylaspis laticarpa	-12.1663	60.3459	357	1
BIOFAR	690	4	Nannastacidae	Campylaspis undata	-12.1663	60.3459	357	1
BIOFAR	690	4	Nannastacidae	Cumellopsis helgae	-12.1663	60.3459	357	1
BIOFAR	690	4	Diastylidae	Diastylis cornuta	-12.1663	60.3459	357	1
BIOFAR	690	4	Diastylidae	Diastylis echinata	-12.1663	60.3459	357	1
BIOFAR	690	4	Diastylidae	Diastylis jonesi	-12.1663	60.3459	357	1
BIOFAR	692	4	Lampropidae	Hemilamprops assimilis	-12.4937	60.2479	300	1
BIOFAR	692	4	Lampropidae	Hemilamprops uniplicatus	-12.4937	60.2479	300	1
BIOFAR	693	4	Lampropidae	Hemilamprops uniplicatus	-12.4840	60.2427	290	1
BIOFAR	694	4	Lampropidae	Hemilamprops assimilis	-10.5995	60.5725	624	1
BIOFAR	694	4	Lampropidae	Hemilamprops cristatus	-10.5995	60.5725	624	1
BIOFAR	694	4	Lampropidae	Platysympus tricarinatus	-10.5995	60.5725	624	1
BIOFAR	694	4	Bodotriidae	Cyclaspis longicaudata	-10.5995	60.5725	624	1
BIOFAR	694	4	Nannastacidae	Campylaspis globosa	-10.5995	60.5725	624	1
BIOFAR	694	4	Nannastacidae	Campylaspis horridoides	-10.5995	60.5725	624	1
BIOFAR	694	4	Nannastacidae	Campylaspis intermedia	-10.5995	60.5725	624	1
BIOFAR	694	4	Nannastacidae	Campylaspis laticarpa	-10.5995	60.5725	624	1
BIOFAR	694	4	Nannastacidae	Campylaspis serratipes	-10.5995	60.5725	624	1
BIOFAR	694	4	Nannastacidae	Campylaspis undata	-10.5995	60.5725	624	1
BIOFAR	696	4	Lampropidae	Hemilamprops assimilis	-10.4640	61.3569	1319	1
BIOFAR	696	4	Lampropidae	Hemilamprops cristatus	-10.4640	61.3569	1319	1
BIOFAR	696	4	Nannastacidae	Campylaspis globosa	-10.4640	61.3569	1319	1

Supplemental Table xx Data source and station information on specimens incorporated in the distribution maps.

Data source	Station	Ecoregion	Taxon 1	Taxon 2	decLong	decLat	minDepth	Specimen count
BIOFAR	696	4	Nannastacidae	Campylaspis intermedia	-10.4640	61.3569	1319	1
BIOFAR	696	4	Nannastacidae	Campylaspis rubicunda	-10.4640	61.3569	1319	1
BIOFAR	696	4	Leuconidae	Leucon (Crymoleucon) noerrevangi	-10.4640	61.3569	1319	1
BIOFAR	696	4	Diastylidae	Diastylis echinata	-10.4640	61.3569	1319	1
BIOFAR	705	4	Lampropidae	Hemilamprops uniplicatus	-7.3102	60.3838	1038	1
BIOFAR	705	4	Nannastacidae	Campylaspis serratipes	-7.3102	60.3838	1038	1
BIOFAR	718	4	Lampropidae	Hemilamprops cristatus	-5.0810	61.0610	496	1
BIOFAR	718	4	Nannastacidae	Campylaspis globosa	-5.0810	61.0610	496	1
BIOFAR	718	4	Nannastacidae	Campylaspis horrida	-5.0810	61.0610	496	1
BIOFAR	718	4	Nannastacidae	Campylaspis serratipes	-5.0810	61.0610	496	1
BIOFAR	718	4	Leuconidae	Leucon (Leucon) nathorsti	-5.0810	61.0610	496	1
BIOFAR	722	4	Lampropidae	Hemilamprops cristatus	-4.4620	61.0670	918	1
BIOFAR	722	4	Nannastacidae	Campylaspis verrucosa	-4.4620	61.0670	918	1
BIOFAR	722	4	Leuconidae	Eudorella arctica	-4.4620	61.0670	918	1
BIOFAR	722	4	Diastylidae	Diastylis echinata	-4.4620	61.0670	918	1
BIOFAR	722	4	Diastylidae	Leptostylis longimana	-4.4620	61.0670	918	1
BIOFAR	729	4	Lampropidae	Hemilamprops uniplicatus	-7.0430	60.3440	850	1
BIOFAR	730	4	Lampropidae	Hemilamprops uniplicatus	-7.0820	60.3220	949	1
BIOFAR	730	4	Leuconidae	Leucon (Crymoleucon) noerrevangi	-7.0820	60.3220	949	1
BIOFAR	731	4	Lampropidae	Hemilamprops uniplicatus	-7.1410	60.2970	1042	1
BIOFAR	731	4	Nannastacidae	Campylaspis intermedia	-7.1410	60.2970	1042	1
BIOFAR	731	4	Nannastacidae	Campylaspis rubicunda	-7.1410	60.2970	1042	1
BIOFAR	731	4	Leuconidae	Leucon (Leucon) nathorsti	-7.1410	60.2970	1042	1
BIOFAR	731	4	Diastylidae	Diastylis echinata	-7.1410	60.2970	1042	1
BIOFAR	731	4	Diastylidae	Diastylis polaris	-7.1410	60.2970	1042	1
BIOFAR	736	4	Ceratocumatidae	Ceratocuma horridum	-10.3207	61.1706	1157	1
BIOFAR	736	4	Lampropidae	Hemilamprops pellucidus	-10.3207	61.1706	1157	1
BIOFAR	736	4	Bodotriidae	Cyclaspis longicaudata	-10.3207	61.1706	1157	1
BIOFAR	736	4	Bodotriidae	Cyclaspoides sarsi	-10.3207	61.1706	1157	1
BIOFAR	736	4	Nannastacidae	Campylaspis horrida	-10.3207	61.1706	1157	1
BIOFAR	736	4	Nannastacidae	Campylaspis rubicunda	-10.3207	61.1706	1157	1
BIOFAR	736	4	Nannastacidae	Cumellopsis helgae	-10.3207	61.1706	1157	1
BIOFAR	736	4	Nannastacidae	Procampylaspis bituberculata	-10.3207	61.1706	1157	1
BIOFAR	736	4	Nannastacidae	Procampylaspis lutensis	-10.3207	61.1706	1157	1
BIOFAR	736	4	Leuconidae	Leucon (Epileucon) longirostris	-10.3207	61.1706	1157	1
BIOFAR	736	4	Leuconidae	Leucon (Leucon) fulvus	-10.3207	61.1706	1157	1
BIOFAR	736	4	Diastylidae	Diastylis echinata	-10.3207	61.1706	1157	1
BIOFAR	738	4	Lampropidae	Hemilamprops cristatus	-10.1330	62.1930	749	1
BIOFAR	738	4	Lampropidae	Platysympus tricarinatus	-10.1330	62.1930	749	1
BIOFAR	738	4	Nannastacidae	Campylaspis globosa	-10.1330	62.1930	749	1
BIOFAR	738	4	Nannastacidae	Campylaspis undata	-10.1330	62.1930	749	1
BIOFAR	738	4	Leuconidae	Leucon (Crymoleucon) noerrevangi	-10.1330	62.1930	749	1
BIOFAR	739	4	Lampropidae	Hemilamprops assimilis	-10.0180	62.2460	630	1
BIOFAR	739	4	Lampropidae	Hemilamprops cristatus	-10.0180	62.2460	630	1
BIOFAR	739	4	Nannastacidae	Campylaspis horrida	-10.0180	62.2460	630	1
BIOFAR	739	4	Nannastacidae	Campylaspis undata	-10.0180	62.2460	630	1
BIOFAR	739	4	Leuconidae	Leucon (Leucon) robustus	-10.0180	62.2460	630	1
BIOFAR	739	4	Diastylidae	Diastylis echinata	-10.0180	62.2460	630	1
BIOFAR	747	4	Lampropidae	Hemilamprops assimilis	-5.5580	62.4340	394	1
BIOFAR	747	4	Nannastacidae	Campylaspis intermedia	-5.5580	62.4340	394	1
BIOFAR	747	4	Nannastacidae	Campylaspis undata	-5.5580	62.4340	394	1
BIOFAR	747	4	Diastylidae	Diastylis echinata	-5.5580	62.4340	394	1
BIOFAR	9012	4	Lampropidae	Hemilamprops uniplicatus	-6.5880	63.0250	1022	1
BIOFAR	9012	4	Leuconidae	Eudorella truncatula	-6.5880	63.0250	1022	1
BIOFAR	9012	4	Diastylidae	Diastylis glabra	-6.5880	63.0250	1022	1
BIOFAR	9012	4	Diastylidae	Diastylis polaris	-6.5880	63.0250	1022	1
BIOFAR	9014	4	Pseudocumatidae	Petalosarsia declivis	-6.5770	62.5711	763	1
BIOFAR	9014	4	Lampropidae	Hemilamprops cristatus	-6.5770	62.5711	763	1
BIOFAR	9014	4	Lampropidae	Hemilamprops uniplicatus	-6.5770	62.5711	763	1
BIOFAR	9014	4	Nannastacidae	Campylaspis affinis	-6.5770	62.5711	763	1
BIOFAR	9014	4	Leuconidae	Eudorella truncatula	-6.5770	62.5711	763	1
BIOFAR	9014	4	Leuconidae	Leucon (Alytoleucon) pallidus	-6.5770	62.5711	763	1
BIOFAR	9014	4	Leuconidae	Leucon (Crymoleucon) noerrevangi	-6.5770	62.5711	763	1
BIOFAR	9014	4	Diastylidae	Diastylis echinata	-6.5770	62.5711	763	1
BIOFAR	9014	4	Diastylidae	Diastylis polaris	-6.5770	62.5711	763	1
BIOFAR	9014	4	Diastylidae	Leptostylis villosa	-6.5770	62.5711	763	1
BIOICE	1051_1	2	Cumacea	Cumacea	-12.3730	66.3010	730.8	1
BIOICE	1090_1	2	Cumacea	Cumacea	-25.0530	66.0005	742.5	6
BIOICE	1090_1	2	Cumacea	Cumacea	-25.0530	66.0005	742.5	10
BIOICE	1090_1	2	Cumacea	Cumacea	-25.0530	66.0005	742.5	30
BIOICE	1104_1	2	Cumacea	Cumacea	-24.5328	66.6433	118.8	15
BIOICE	1104_1	2	Cumacea	Cumacea	-24.5328	66.6433	118.8	5
BIOICE	1116_1	2	Cumacea	Cumacea	-26.2718	67.2137	683.1	4
BIOICE	1116_1	2	Cumacea	Cumacea	-26.2718	67.2137	683.1	1
BIOICE	1116_1	2	Cumacea	Cumacea	-26.2718	67.2137	683.1	1
BIOICE	1119_1	2	Cumacea	Cumacea	-26.2417	67.2135	696.9	45
BIOICE	1119_1	2	Cumacea	Cumacea	-26.2417	67.2135	696.9	20
BIOICE	1119_1	2	Cumacea	Cumacea	-26.2417	67.2135	696.9	1
BIOICE	1123_1	2	Cumacea	Cumacea	-26.2075	67.2138	716.5	46

Supplemental Table xx Data source and station information on specimens incorporated in the distribution maps.

Data source	Station	Ecoregion	Taxon 1	Taxon 2	decLong	decLat	minDepth	Specimen count
BIOICE	1123_1	2	Cumacea	Cumacea	-26.2075	67.2138	716.5	115
BIOICE	1123_1	2	Cumacea	Cumacea	-26.2075	67.2138	716.5	126
BIOICE	1123_1	2	Cumacea	Cumacea	-26.2075	67.2138	716.5	28
BIOICE	1123_1	2	Cumacea	Cumacea	-26.2075	67.2138	716.5	13
BIOICE	1129_1	2	Cumacea	Cumacea	-26.7463	67.6462	320.6	13
BIOICE	1129_1	2	Cumacea	Cumacea	-26.7463	67.6462	320.6	33
BIOICE	1129_1	2	Cumacea	Cumacea	-26.7463	67.6462	320.6	1
BIOICE	1129_1	2	Cumacea	Cumacea	-26.7463	67.6462	320.6	9
BIOICE	1129_1	2	Cumacea	Cumacea	-26.7463	67.6462	320.6	1
BIOICE	1129_1	2	Cumacea	Cumacea	-26.7463	67.6462	320.6	1
BIOICE	1129_1	2	Cumacea	Cumacea	-26.7463	67.6462	320.6	6
BIOICE	1132_1	2	Cumacea	Cumacea	-26.7547	67.6413	318.1	2
BIOICE	1132_1	2	Cumacea	Cumacea	-26.7547	67.6413	318.1	11
BIOICE	1132_1	2	Cumacea	Cumacea	-26.7547	67.6413	318.1	21
BIOICE	1136_1	2	Cumacea	Cumacea	-26.7665	67.6358	315.9	148
BIOICE	1136_1	2	Cumacea	Cumacea	-26.7665	67.6358	315.9	13
BIOICE	1136_1	2	Cumacea	Cumacea	-26.7665	67.6358	315.9	30
BIOICE	1136_1	2	Cumacea	Cumacea	-26.7665	67.6358	315.9	58
BIOICE	1144_1	2	Cumacea	Cumacea	-23.6963	67.8678	1281	36
BIOICE	1144_1	2	Cumacea	Cumacea	-23.6963	67.8678	1281	7
BIOICE	1148_1	2	Cumacea	Cumacea	-23.6960	67.8465	1248.8	19
BIOICE	1148_1	2	Cumacea	Cumacea	-23.6960	67.8465	1248.8	256
BIOICE	1148_1	2	Cumacea	Cumacea	-23.6960	67.8465	1248.8	106
BIOICE	1148_1	2	Cumacea	Cumacea	-23.6960	67.8465	1248.8	16
BIOICE	1152_1	2	Cumacea	Cumacea	-9.9335	69.0933	2172.6	2
BIOICE	1153_1	2	Cumacea	Cumacea	-9.9335	69.0933	2173.4	2
BIOICE	1155_1	2	Cumacea	Cumacea	-9.9120	69.1148	2203.8	8
BIOICE	1155_1	2	Cumacea	Cumacea	-9.9120	69.1148	2203.8	2
BIOICE	1155_1	2	Cumacea	Cumacea	-9.9120	69.1148	2203.8	2
BIOICE	1155_1	2	Cumacea	Cumacea	-9.9120	69.1148	2203.8	1
BIOICE	1159_1	2	Cumacea	Cumacea	-9.9170	69.1110	2202.8	1
BIOICE	1159_1	2	Cumacea	Cumacea	-9.9170	69.1110	2202.8	64
BIOICE	1159_1	2	Cumacea	Cumacea	-9.9170	69.1110	2202.8	3
BIOICE	1159_1	2	Cumacea	Cumacea	-9.9170	69.1110	2202.8	1
BIOICE	1168_1	2	Cumacea	Cumacea	-7.0013	67.6063	2372.6	1
BIOICE	1172_1	2	Cumacea	Cumacea	-6.9347	67.5782	2422.4	1
BIOICE	1184_1	2	Cumacea	Cumacea	-12.1620	67.6438	1819.3	31
BIOICE	1184_1	2	Cumacea	Cumacea	-12.1620	67.6438	1819.3	74
BIOICE	1184_1	2	Cumacea	Cumacea	-12.1620	67.6438	1819.3	32
BIOICE	1184_1	2	Cumacea	Cumacea	-12.1620	67.6438	1819.3	74
BIOICE	1184_1	2	Cumacea	Cumacea	-12.1620	67.6438	1819.3	3
BIOICE	1191_1	2	Cumacea	Cumacea	-13.0638	67.0787	1574.7	7
BIOICE	1191_1	2	Cumacea	Cumacea	-13.0638	67.0787	1574.7	1
BIOICE	1191_1	2	Cumacea	Cumacea	-13.0638	67.0787	1574.7	3
BIOICE	1191_1	2	Cumacea	Cumacea	-13.0638	67.0787	1574.7	4
BIOICE	1209_1	2	Cumacea	Cumacea	-12.8648	66.5382	315.9	435
BIOICE	1209_1	2	Cumacea	Cumacea	-12.8648	66.5382	315.9	227
BIOICE	1209_1	2	Cumacea	Cumacea	-12.8648	66.5382	315.9	225
BIOICE	1209_1	2	Cumacea	Cumacea	-12.8648	66.5382	315.9	207
BIOICE	1212_1	2	Cumacea	Cumacea	-12.8747	66.5438	317.2	264
BIOICE	1216_1	2	Cumacea	Cumacea	-12.3730	66.3010	730.8	1
BIOICE	1216_1	2	Cumacea	Cumacea	-12.3730	66.3010	730.8	7
BIOICE	1216_1	2	Cumacea	Cumacea	-12.3730	66.3010	730.8	2
BIOICE	1216_1	2	Cumacea	Cumacea	-12.3730	66.3010	730.8	15
BIOICE	1216_1	2	Cumacea	Cumacea	-12.3730	66.3010	730.8	1
BIOICE	1216_1	2	Cumacea	Cumacea	-12.3730	66.3010	730.8	1
BIOICE	1216_1	2	Cumacea	Cumacea	-12.3730	66.3010	730.8	3
BIOICE	1216_1	2	Cumacea	Cumacea	-12.3730	66.3010	730.8	1
BIOICE	1219_1	2	Cumacea	Cumacea	-12.3470	66.2890	579.1	149
BIOICE	1219_1	2	Cumacea	Cumacea	-12.3470	66.2890	579.1	203
BIOICE	1219_1	2	Cumacea	Cumacea	-12.3470	66.2890	579.1	200
BIOICE	1219_1	2	Cumacea	Cumacea	-12.3470	66.2890	579.1	200
BIOICE	1219_1	2	Cumacea	Cumacea	-12.3470	66.2890	579.1	21
BIOICE	1222_1	2	Cumacea	Cumacea	-12.3515	66.2915	610.8	8
BIOICE	983_1	4	Cumacea	Cumacea	-18.1357	60.3573	2567.7	57
BIOICE	983_1	4	Cumacea	Cumacea	-18.1357	60.3573	2567.7	87
BIOICE	983_1	4	Cumacea	Cumacea	-18.1357	60.3573	2567.7	398
BIOICE	983_1	4	Cumacea	Cumacea	-18.1357	60.3573	2567.7	14
BIOICE	989_1	4	Cumacea	Cumacea	-19.5493	61.7105	1912.3	18
BIOICE	989_1	4	Cumacea	Cumacea	-19.5493	61.7105	1912.3	26
BIOICE	989_1	4	Cumacea	Cumacea	-19.5493	61.7105	1912.3	1
BIOICE	996_1	4	Cumacea	Cumacea	-19.5463	61.7082	1913	6
BIOICE	1002_1	4	Cumacea	Cumacea	-20.3530	62.5583	1392.4	1
BIOICE	1002_1	4	Cumacea	Cumacea	-20.3530	62.5583	1392.4	4
BIOICE	1002_1	4	Cumacea	Cumacea	-20.3530	62.5583	1392.4	2
BIOICE	1002_1	4	Cumacea	Cumacea	-20.3530	62.5583	1392.4	1
BIOICE	1002_1	4	Cumacea	Cumacea	-20.3530	62.5583	1392.4	1
BIOICE	1002_1	4	Cumacea	Cumacea	-20.3530	62.5583	1392.4	3
BIOICE	1003_1	4	Cumacea	Cumacea	-20.3530	62.5583	1390	1

Supplemental Table xx Data source and station information on specimens incorporated in the distribution maps.

Data source	Station	Ecoregion	Taxon 1	Taxon 2	decLong	decLat	minDepth	Specimen count
BIOICE	1006_1	4	Cumacea	Cumacea	-20.3750	62.5508	1386.8	4
BIOICE	1006_1	4	Cumacea	Cumacea	-20.3750	62.5508	1386.8	2
BIOICE	1006_1	4	Cumacea	Cumacea	-20.3750	62.5508	1386.8	2
BIOICE	1010_1	4	Cumacea	Cumacea	-20.3952	62.5517	1384.8	4
BIOICE	1010_1	4	Cumacea	Cumacea	-20.3952	62.5517	1384.8	9
BIOICE	1010_1	4	Cumacea	Cumacea	-20.3952	62.5517	1384.8	2
BIOICE	1010_1	4	Cumacea	Cumacea	-20.3952	62.5517	1384.8	15
BIOICE	1010_1	4	Cumacea	Cumacea	-20.3952	62.5517	1384.8	13
BIOICE	1010_1	4	Cumacea	Cumacea	-20.3952	62.5517	1384.8	124
BIOICE	1010_1	4	Cumacea	Cumacea	-20.3952	62.5517	1384.8	57
BIOICE	1010_1	4	Cumacea	Cumacea	-20.3952	62.5517	1384.8	144
BIOICE	1010_1	4	Cumacea	Cumacea	-20.3952	62.5517	1384.8	85
BIOICE	1010_1	4	Cumacea	Cumacea	-20.3952	62.5517	1384.8	12
BIOICE	1010_1	4	Cumacea	Cumacea	-20.3952	62.5517	1384.8	1
BIOICE	1017_1	4	Cumacea	Cumacea	-20.7738	62.9307	891.7	1
BIOICE	1017_1	4	Cumacea	Cumacea	-20.7738	62.9307	891.7	3
BIOICE	1017_1	4	Cumacea	Cumacea	-20.7738	62.9307	891.7	3
BIOICE	1019_1	4	Cumacea	Cumacea	-20.7435	62.9387	913.6	11
BIOICE	1019_1	4	Cumacea	Cumacea	-20.7435	62.9387	913.6	2
BIOICE	1019_1	4	Cumacea	Cumacea	-20.7435	62.9387	913.6	55
BIOICE	1019_1	4	Cumacea	Cumacea	-20.7435	62.9387	913.6	52
BIOICE	1022_1	4	Cumacea	Cumacea	-20.7893	62.9263	906.8	3
BIOICE	1031_1	4	Cumacea	Cumacea	-23.1667	63.3333	305.3	1
BIOICE	1032_1	4	Cumacea	Cumacea	-23.1577	63.3085	289.4	2
BIOICE	1032_1	4	Cumacea	Cumacea	-23.1577	63.3085	289.4	29
BIOICE	1032_1	4	Cumacea	Cumacea	-23.1577	63.3085	289.4	238
BIOICE	1032_1	4	Cumacea	Cumacea	-23.1577	63.3085	289.4	4
BIOICE	1033_1	4	Cumacea	Cumacea	-23.1602	63.3147	288.5	1
BIOICE	1033_1	4	Cumacea	Cumacea	-23.1602	63.3147	288.5	2
BIOICE	1033_1	4	Cumacea	Cumacea	-23.1602	63.3147	288.5	3
BIOICE	1033_1	4	Cumacea	Cumacea	-23.1602	63.3147	288.5	1
BIOICE	1033_1	4	Cumacea	Cumacea	-23.1602	63.3147	288.5	3
BIOICE	1033_1	4	Cumacea	Cumacea	-23.1602	63.3147	288.5	6
BIOICE	1033_1	4	Cumacea	Cumacea	-23.1602	63.3147	288.5	9
BIOICE	1041_1	4	Cumacea	Cumacea	-25.9642	63.9227	214.9	1
BIOICE	1043_1	4	Cumacea	Cumacea	-25.9610	63.9243	213.9	1
BIOICE	1043_1	4	Cumacea	Cumacea	-25.9610	63.9243	213.9	1
BIOICE	1043_1	4	Cumacea	Cumacea	-25.9610	63.9243	213.9	4
BIOICE	1043_1	4	Cumacea	Cumacea	-25.9610	63.9243	213.9	5
BIOICE	1043_1	4	Cumacea	Cumacea	-25.9610	63.9243	213.9	47
BIOICE	1043_1	4	Cumacea	Cumacea	-25.9610	63.9243	213.9	166
BIOICE	1045_1	4	Cumacea	Cumacea	-25.9535	63.9283	218.4	13
BIOICE	1045_1	4	Cumacea	Cumacea	-25.9535	63.9283	218.4	5
BIOICE	1045_1	4	Cumacea	Cumacea	-25.9535	63.9283	218.4	1
BIOICE	1045_1	4	Cumacea	Cumacea	-25.9535	63.9283	218.4	17
BIOICE	1054_1	4	Cumacea	Cumacea	-31.3767	61.6032	2537.3	162
BIOICE	1054_1	4	Cumacea	Cumacea	-31.3767	61.6032	2537.3	15
BIOICE	1054_1	4	Cumacea	Cumacea	-31.3767	61.6032	2537.3	3
BIOICE	1057_1	4	Cumacea	Cumacea	-31.3562	61.6417	2504.7	1
BIOICE	1057_1	4	Cumacea	Cumacea	-31.3562	61.6417	2504.7	2
BIOICE	1057_1	4	Cumacea	Cumacea	-31.3562	61.6417	2504.7	2
BIOICE	1057_1	4	Cumacea	Cumacea	-31.3562	61.6417	2504.7	3
BIOICE	1057_1	4	Cumacea	Cumacea	-31.3562	61.6417	2504.7	1
BIOICE	1057_1	4	Cumacea	Cumacea	-31.3562	61.6417	2504.7	19
BIOICE	1069_1	4	Cumacea	Cumacea	-28.0950	62.9888	1588.2	1
BIOICE	1069_1	4	Cumacea	Cumacea	-28.0950	62.9888	1588.2	4
BIOICE	1072_1	4	Cumacea	Cumacea	-28.0682	63.0077	1593.8	1
BIOICE	1072_1	4	Cumacea	Cumacea	-28.0682	63.0077	1593.8	8
BIOICE	1072_1	4	Cumacea	Cumacea	-28.0682	63.0077	1593.8	35
BIOICE	1072_1	4	Cumacea	Cumacea	-28.0682	63.0077	1593.8	49
BIOICE	1072_1	4	Cumacea	Cumacea	-28.0682	63.0077	1593.8	63
BIOICE	1072_1	4	Cumacea	Cumacea	-28.0682	63.0077	1593.8	73
BIOICE	1080_1	4	Cumacea	Cumacea	-26.4073	63.6983	741	2
BIOICE	1080_1	4	Cumacea	Cumacea	-26.4073	63.6983	741	3
BIOICE	1082_1	4	Cumacea	Cumacea	-26.3940	63.7017	724.4	1
BIOICE	1082_1	4	Cumacea	Cumacea	-26.3940	63.7017	724.4	2
BIOICE	1082_1	4	Cumacea	Cumacea	-26.3940	63.7017	724.4	11
BIOICE	1086_1	4	Cumacea	Cumacea	-26.3842	63.7088	698.1	134
BIOICE	1086_1	4	Cumacea	Cumacea	-26.3842	63.7088	698.1	7
BIOICE	1086_1	4	Cumacea	Cumacea	-26.3842	63.7088	698.1	195
BIOICE	1086_1	4	Cumacea	Cumacea	-26.3842	63.7088	698.1	15
BIOICE	1086_1	4	Cumacea	Cumacea	-26.3842	63.7088	698.1	106
BIOICE	866_7	5	Cumacea	Cumacea	1.3512	61.4272	169.1	59
BIOICE	866_7	5	Cumacea	Cumacea	1.3512	61.4272	169.1	50
BIOICE	866_7	5	Cumacea	Cumacea	1.3512	61.4272	169.1	77
BIOICE	866_7	5	Cumacea	Cumacea	1.3512	61.4272	169.1	1
BIOICE	866_7	5	Cumacea	Cumacea	1.3512	61.4272	169.1	12
BIOICE	866_7	5	Cumacea	Cumacea	1.3512	61.4272	169.1	2
BIOICE	866_7	5	Cumacea	Cumacea	1.3512	61.4272	169.1	330

Supplemental Table xx Data source and station information on specimens incorporated in the distribution maps.

Data source	Station	Ecoregion	Taxon 1	Taxon 2	decLong	decLat	minDepth	Specimen count
BIOICE	866_7	5	Cumacea	Cumacea	1.3512	61.4272	169.1	124
BIOICE	866_7	5	Cumacea	Cumacea	1.3512	61.4272	169.1	581
BIOICE	867_1	5	Cumacea	Cumacea	0.5067	61.9972	302.5	4
BIOICE	867_1	5	Cumacea	Cumacea	0.5067	61.9972	302.5	21
BIOICE	867_1	5	Cumacea	Cumacea	0.5067	61.9972	302.5	21
BIOICE	867_1	5	Cumacea	Cumacea	0.5067	61.9972	302.5	27
BIOICE	867_1	5	Cumacea	Cumacea	0.5067	61.9972	302.5	207
BIOICE	868_3	5	Cumacea	Cumacea	0.2585	62.1523	587.4	4
BIOICE	868_3	5	Cumacea	Cumacea	0.2585	62.1523	587.4	62
BIOICE	868_3	5	Cumacea	Cumacea	0.2585	62.1523	587.4	8
BIOICE	868_3	5	Cumacea	Cumacea	0.2585	62.1523	587.4	57
BIOICE	868_3	5	Cumacea	Cumacea	0.2585	62.1523	587.4	17
BIOICE	868_3	5	Cumacea	Cumacea	0.2585	62.1523	587.4	481
BIOICE	869_3	5	Cumacea	Cumacea	0.0202	62.2700	846.4	1
BIOICE	869_3	5	Cumacea	Cumacea	0.0202	62.2700	846.4	5
BIOICE	869_3	5	Cumacea	Cumacea	0.0202	62.2700	846.4	2
IceAGE 2	873_6	4	Cumacea	Cumacea	-3.8730	61.7753	833.7	1
IceAGE 2	873_6	4	Cumacea	Cumacea	-3.8730	61.7753	833.7	15
IceAGE 2	873_6	4	Cumacea	Cumacea	-3.8730	61.7753	833.7	2
IceAGE 2	873_6	4	Cumacea	Cumacea	-3.8730	61.7753	833.7	39
IceAGE 2	874_2	4	Cumacea	Cumacea	-4.3663	61.5470	901.8	15
IceAGE 2	874_2	4	Cumacea	Cumacea	-4.3663	61.5470	901.8	5
IceAGE 2	874_2	4	Cumacea	Cumacea	-4.3663	61.5470	901.8	7
IceAGE 2	876_5	4	Cumacea	Cumacea	-6.6152	60.4055	554.3	2
IceAGE 2	876_5	4	Cumacea	Cumacea	-6.6152	60.4055	554.3	1
IceAGE 2	879_5	4	Cumacea	Cumacea	-8.5720	63.1017	510.9	47
IceAGE 2	879_5	4	Cumacea	Cumacea	-8.5720	63.1017	510.9	2
IceAGE 2	879_5	4	Cumacea	Cumacea	-8.5720	63.1017	510.9	71
IceAGE 2	879_5	4	Cumacea	Cumacea	-8.5720	63.1017	510.9	115
IceAGE 2	879_5	4	Cumacea	Cumacea	-8.5720	63.1017	510.9	699
IceAGE 2	880_2	4	Cumacea	Cumacea	-8.1570	63.3893	686	51
IceAGE 2	880_2	4	Cumacea	Cumacea	-8.1570	63.3893	686	28
IceAGE 2	880_2	4	Cumacea	Cumacea	-8.1570	63.3893	686	5
IceAGE 2	880_2	4	Cumacea	Cumacea	-8.1570	63.3893	686	176
IceAGE 2	880_2	4	Cumacea	Cumacea	-8.1570	63.3893	686	169
IceAGE 2	881_4	4	Cumacea	Cumacea	-7.7115	63.5777	1043.6	7
IceAGE 2	881_4	4	Cumacea	Cumacea	-7.7115	63.5777	1043.6	29
IceAGE 2	881_4	4	Cumacea	Cumacea	-7.7115	63.5777	1043.6	2
IceAGE 2	881_4	4	Cumacea	Cumacea	-7.7115	63.5777	1043.6	9
IceAGE 2	881_4	4	Cumacea	Cumacea	-7.7115	63.5777	1043.6	30
IceAGE 2	881_4	4	Cumacea	Cumacea	-7.7115	63.5777	1043.6	9
IceAGE 2	881_4	4	Cumacea	Cumacea	-7.7115	63.5777	1043.6	5
IceAGE 2	881_5	4	Cumacea	Cumacea	-7.7535	63.6090	1056.2	85
IceAGE 2	881_5	4	Cumacea	Cumacea	-7.7535	63.6090	1056.2	3
IceAGE 2	881_5	4	Cumacea	Cumacea	-7.7535	63.6090	1056.2	186
IceAGE 2	881_5	4	Cumacea	Cumacea	-7.7535	63.6090	1056.2	82
IceAGE 2	881_5	4	Cumacea	Cumacea	-7.7535	63.6090	1056.2	11
IceAGE 2	882_5	4	Cumacea	Cumacea	-10.9700	63.4173	440.5	9
IceAGE 2	882_5	4	Cumacea	Cumacea	-10.9700	63.4173	440.5	27
IceAGE 2	882_5	4	Cumacea	Cumacea	-10.9700	63.4173	440.5	5
IceAGE 2	882_5	4	Cumacea	Cumacea	-10.9700	63.4173	440.5	20
IceAGE 2	882_5	4	Cumacea	Cumacea	-10.9700	63.4173	440.5	167
IceAGE 2	882_5	4	Cumacea	Cumacea	-10.9700	63.4173	440.5	839
IceAGE 2	866_7	5	Cumacea	Cumacea	1.3512	61.4272	169.1	59
IceAGE 2	866_7	5	Cumacea	Cumacea	1.3512	61.4272	169.1	50
IceAGE 2	866_7	5	Cumacea	Cumacea	1.3512	61.4272	169.1	77
IceAGE 2	866_7	5	Cumacea	Cumacea	1.3512	61.4272	169.1	1
IceAGE 2	866_7	5	Cumacea	Cumacea	1.3512	61.4272	169.1	12
IceAGE 2	866_7	5	Cumacea	Cumacea	1.3512	61.4272	169.1	2
IceAGE 2	866_7	5	Cumacea	Cumacea	1.3512	61.4272	169.1	330
IceAGE 2	866_7	5	Cumacea	Cumacea	1.3512	61.4272	169.1	124
IceAGE 2	866_7	5	Cumacea	Cumacea	1.3512	61.4272	169.1	581
IceAGE 2	867_1	5	Cumacea	Cumacea	0.5067	61.9972	302.5	4
IceAGE 2	867_1	5	Cumacea	Cumacea	0.5067	61.9972	302.5	21
IceAGE 2	867_1	5	Cumacea	Cumacea	0.5067	61.9972	302.5	21
IceAGE 2	867_1	5	Cumacea	Cumacea	0.5067	61.9972	302.5	27
IceAGE 2	867_1	5	Cumacea	Cumacea	0.5067	61.9972	302.5	207
IceAGE 2	868_3	5	Cumacea	Cumacea	0.2585	62.1523	587.4	4
IceAGE 2	868_3	5	Cumacea	Cumacea	0.2585	62.1523	587.4	62
IceAGE 2	868_3	5	Cumacea	Cumacea	0.2585	62.1523	587.4	8
IceAGE 2	868_3	5	Cumacea	Cumacea	0.2585	62.1523	587.4	57
IceAGE 2	868_3	5	Cumacea	Cumacea	0.2585	62.1523	587.4	17
IceAGE 2	868_3	5	Cumacea	Cumacea	0.2585	62.1523	587.4	481
IceAGE 2	869_3	5	Cumacea	Cumacea	0.0202	62.2700	846.4	1
IceAGE 2	869_3	5	Cumacea	Cumacea	0.0202	62.2700	846.4	5
IceAGE 2	869_3	5	Cumacea	Cumacea	0.0202	62.2700	846.4	2
IceAGE 2	869_3	5	Cumacea	Cumacea	0.0202	62.2700	846.4	43
IceAGE 2	869_3	5	Cumacea	Cumacea	0.0202	62.2700	846.4	15
IceAGE 2	869_3	5	Cumacea	Cumacea	0.0202	62.2700	846.4	63

Supplemental Table xx Data source and station information on specimens incorporated in the distribution maps.

Data source	Station	Ecoregion	Taxon 1	Taxon 2	decLong	decLat	minDepth	Specimen count
IceAGE 2	870_4	5	Cumacea	Cumacea	-0.1017	62.3288	1058.4	23
IceAGE 2	870_4	5	Cumacea	Cumacea	-0.1017	62.3288	1058.4	6
IceAGE 2	870_4	5	Cumacea	Cumacea	-0.1017	62.3288	1058.4	6
IceAGE 2	870_4	5	Cumacea	Cumacea	-0.1017	62.3288	1058.4	202
IceAGE 2	870_4	5	Cumacea	Cumacea	-0.1017	62.3288	1058.4	63
IceAGE 2	871_1	5	Cumacea	Cumacea	-0.9463	62.7372	1577.4	7
IceAGE 2	871_1	5	Cumacea	Cumacea	-0.9463	62.7372	1577.4	10
IceAGE 2	871_1	5	Cumacea	Cumacea	-0.9463	62.7372	1577.4	26
IceAGE 2	872_4	5	Cumacea	Cumacea	-1.4985	63.0313	1858.3	19
IceAGE 2	872_4	5	Cumacea	Cumacea	-1.4985	63.0313	1858.3	38
IceAGE 2	872_4	5	Cumacea	Cumacea	-1.4985	63.0313	1858.3	40
MAREANO	NA	1	Nannastacidae	Campylaspis affinis	16.9354	80.1524	340	1
MAREANO	NA	1	Nannastacidae	Campylaspis affinis	19.2967	81.0011	168	1
MAREANO	NA	1	Nannastacidae	Campylaspis affinis	12.3368	80.4576	497	1
MAREANO	NA	1	Nannastacidae	Campylaspis costata	19.2967	81.0011	168	1
MAREANO	NA	1	Nannastacidae	Campylaspis globosa	12.3368	80.4576	497	1
MAREANO	NA	1	Nannastacidae	Campylaspis globosa	19.2967	81.0011	168	1
MAREANO	NA	1	Nannastacidae	Campylaspis intermedia	16.9354	80.1524	340	1
MAREANO	NA	1	Nannastacidae	Campylaspis intermedia	16.9354	80.1524	340	1
MAREANO	NA	1	Nannastacidae	Campylaspis rubicunda	22.1414	80.1086	216	1
MAREANO	NA	1	Diastylidae	Diastylis edwardsii	19.2967	81.0011	168	1
MAREANO	NA	1	Diastylidae	Diastylis edwardsii	22.1532	80.3169	245	1
MAREANO	NA	1	Diastylidae	Diastylis edwardsii	16.9354	80.1524	340	1
MAREANO	NA	1	Diastylidae	Diastylis goodsiri	22.1684	80.0968	215	1
MAREANO	NA	1	Diastylidae	Diastylis goodsiri	12.7806	80.4568	452	1
MAREANO	NA	1	Diastylidae	Diastylis goodsiri	22.1972	80.0835	188	1
MAREANO	NA	1	Diastylidae	Diastylis spinulosa	22.1011	80.6486	126	1
MAREANO	NA	1	Diastylidae	Diastylis spinulosa	12.7806	80.4568	452	1
MAREANO	NA	1	Diastylidae	Diastylis spinulosa	12.3368	80.4576	497	1
MAREANO	NA	1	Diastylidae	Diastylis spinulosa	19.2967	81.0011	168	1
MAREANO	NA	1	Diastylidae	Diastylis spinulosa	22.1414	80.1086	216	1
MAREANO	NA	1	Diastylidae	Diastylis stygia	16.7371	81.1876	1389	1
MAREANO	NA	1	Leuconidae	Eudorella emarginata	11.1145	79.6937	212	1
MAREANO	NA	1	Leuconidae	Eudorella emarginata	22.1414	80.1086	216	1
MAREANO	NA	1	Leuconidae	Eudorella truncatula	12.3368	80.4576	497	1
MAREANO	NA	1	Diastylidae	Leptostylis ampullacea	12.3368	80.4576	497	1
MAREANO	NA	1	Diastylidae	Leptostylis longimana (G.O. Sars, 1865)	16.7371	81.1876	1389	1
MAREANO	NA	1	Leuconidae	Leucon (Leucon) nasica	22.1414	80.1086	216	1
MAREANO	NA	1	Leuconidae	Leucon (Leucon) nathorsti	12.3368	80.4576	497	1
MAREANO	NA	1	Lampropidae	Platysympus tricarinatus	12.3368	80.4576	497	1
MAREANO	NA	1	Lampropidae	Platysympus tricarinatus	12.7806	80.4568	452	1
MAREANO	NA	2	Diastylidae	Brachydiastylis resima	-8.5400	70.3830	426	1
MAREANO	NA	2	Diastylidae	Brachydiastylis resima	-8.5330	70.3960	296	1
MAREANO	NA	2	Nannastacidae	Campylaspis affinis	-11.6110	67.6580	1811	1
MAREANO	NA	2	Nannastacidae	Campylaspis affinis	-9.9100	69.6060	2212	1
MAREANO	NA	2	Nannastacidae	Campylaspis intermedia cf.	-8.4100	69.0230	879	1
MAREANO	NA	2	Nannastacidae	Campylaspis costata	-6.1660	69.9760	2574	1
MAREANO	NA	2	Nannastacidae	Campylaspis rubicunda	-8.5400	70.3830	426	1
MAREANO	NA	2	Nannastacidae	Campylaspis rubicunda	-8.5330	70.3960	296	1
MAREANO	NA	2	Diastylidae	Diastylis edwardsii	-8.5560	70.7730	128	1
MAREANO	NA	2	Diastylidae	Diastylis goodsiri	-8.5400	70.3830	426	1
MAREANO	NA	2	Diastylidae	Diastylis goodsiri	-8.5330	70.3960	296	1
MAREANO	NA	2	Diastylidae	Diastylis goodsiri	-8.4830	70.2550	515	1
MAREANO	NA	2	Diastylidae	Diastylis lucifera	-6.1660	69.9760	2574	1
MAREANO	NA	2	Diastylidae	Diastylis polaris	-8.4100	69.0230	879	1
MAREANO	NA	2	Diastylidae	Diastylis polaris	-7.6310	70.6780	1243	1
MAREANO	NA	2	Diastylidae	Diastylis polaris	-8.3850	70.1780	663	1
MAREANO	NA	2	Diastylidae	Diastylis stygia	-10.4910	68.7060	2168	1
MAREANO	NA	2	Diastylidae	Diastylis stygia	-9.9100	69.6060	2212	1
MAREANO	NA	2	Diastylidae	Diastylis stygia	-6.5300	70.4360	2525	1
MAREANO	NA	2	Diastylidae	Diastylis stygia	-18.1480	69.9550	1618	1
MAREANO	NA	2	Diastylidae	Diastylis stygia	-17.9930	69.9930	1632	1
MAREANO	NA	2	Diastylidae	Diastylis stygia	-12.5660	69.9680	1850	1
MAREANO	NA	2	Diastylidae	Diastylis stygia	-9.5100	69.5000	2200	1
MAREANO	NA	2	Diastylidae	Diastylis stygia	-8.3850	70.1780	663	1
MAREANO	NA	2	Diastylidae	Diastylis stygia	-6.1660	69.9760	2574	1
MAREANO	NA	2	Leuconidae	Eudorella hirsuta	-8.4100	69.0230	879	1
MAREANO	NA	2	Lampropidae	Hemilamprops assimilis	-6.7880	67.0280	2407	1
MAREANO	NA	2	Lampropidae	Hemilamprops roseus	-6.1660	69.9760	2574	1
MAREANO	NA	2	Diastylidae	Leptostylis longimana (G.O. Sars, 1865)	-18.1480	69.9550	1618	1
MAREANO	NA	2	Leuconidae	Leucon (Leucon) nasica	-18.1480	69.9550	1618	1
MAREANO	NA	2	Leuconidae	Leucon (Leucon) nathorsti	-8.5400	70.3830	426	1
MAREANO	NA	2	Leuconidae	Leucon pallidus	-9.9100	69.6060	2212	1
MAREANO	NA	2	Leuconidae	Leucon pallidus	-18.1480	69.9550	1618	1
MAREANO	NA	2	Leuconidae	Leucon pallidus	-8.5330	70.3960	296	1
MAREANO	NA	2	Leuconidae	Leucon spinulosus	-14.2380	68.8910	1588	1
MAREANO	NA	2	Leuconidae	Leucon spinulosus	-11.6110	67.6580	1811	1
MAREANO	NA	2	Leuconidae	Leucon spinulosus	-6.7880	67.0280	2407	1
MAREANO	NA	4	Diastylidae	Brachydiastylis resima	-11.1700	64.4350	400	1

Supplemental Table xx Data source and station information on specimens incorporated in the distribution maps.

Data source	Station	Ecoregion	Taxon 1	Taxon 2	decLong	decLat	minDepth	Specimen count
MAREANO	NA	4	Nannastacidae	Campylaspis affinis	-12.8530	63.5810	574	1
MAREANO	NA	4	Nannastacidae	Campylaspis horrida	-3.1850	61.3430	1338	1
MAREANO	NA	4	Nannastacidae	Campylaspis rubicunda	1.9900	58.5060	87	1
MAREANO	NA	4	Nannastacidae	Campylaspis undata	-12.8530	63.5810	574	1
MAREANO	NA	4	Diastylidae	Diastylis echinata	-6.6250	60.2010	1220	1
MAREANO	NA	4	Diastylidae	Diastylis echinata	-12.8530	63.5810	574	1
MAREANO	NA	4	Diastylidae	Diastylis echinata	-7.5880	65.4600	1626	1
MAREANO	NA	4	Diastylidae	Diastylis edwardsii	-11.1700	64.4350	400	1
MAREANO	NA	4	Diastylidae	Diastylis polaris	-7.0020	62.9480	748	1
MAREANO	NA	4	Diastylidae	Diastylis stygia	-6.6250	60.2010	1220	1
MAREANO	NA	4	Diastylidae	Diastylis stygia	-7.5880	65.4600	1626	1
MAREANO	NA	4	Diastylidae	Diastyloides biplicatus	1.9900	58.5060	87	1
MAREANO	NA	4	Leuconidae	Eudorella truncatula	-11.1700	64.4350	400	1
MAREANO	NA	4	Leuconidae	Eudorellopsis deformis	1.9900	58.5060	87	1
MAREANO	NA	4	Lampropidae	Hemilamprops assimilis	-12.8530	63.5810	574	1
MAREANO	NA	4	Lampropidae	Hemilamprops cristatus	-12.8530	63.5810	574	1
MAREANO	NA	4	Lampropidae	Hemilamprops uniplicatus	-12.8530	63.5810	574	1
MAREANO	NA	4	Lampropidae	Hemilamprops uniplicatus	-11.1700	64.4350	400	1
MAREANO	NA	4	Diastylidae	Leptostylis longimana (G.O. Sars, 1865)	-3.1850	61.3430	1338	1
MAREANO	NA	4	Diastylidae	Leptostylis longimana (G.O. Sars, 1865)	-7.5880	65.4600	1626	1
MAREANO	NA	4	Leuconidae	Leucon (Leucon) acutirostris	-11.1700	64.4350	400	1
MAREANO	NA	4	Leuconidae	Leucon (Crymoleucon) noerrevangi	-7.0020	62.9480	748	1
MAREANO	NA	4	Leuconidae	Leucon pallidus	-3.1850	61.3430	1338	1
MAREANO	NA	4	Leuconidae	Leucon spinulosus	-7.6530	64.7960	2680	1
MAREANO	NA	4	Pseudocumatidae	Petalosarsia declivis	1.9900	58.5060	87	1
MAREANO	NA	4	Pseudocumatidae	Pseudocuma (Pseudocuma) simile	1.9900	58.5060	87	1
MAREANO	NA	4	Nannastacidae	Campylaspis costata	10.4184	58.5122	275-291	1
MAREANO	NA	4	Nannastacidae	Campylaspis costata	10.3081	58.5863	287-297	1
MAREANO	NA	4	Nannastacidae	Campylaspis globosa	10.1333	58.4695	497-513	1
MAREANO	NA	4	Nannastacidae	Campylaspis rubicunda	10.3081	58.5863	287-297	1
MAREANO	NA	4	Diastylidae	Diastylis laevis cf.	10.6061	58.9115	276-306	1
MAREANO	NA	4	Diastylidae	Diastylis laevis	10.4184	58.5122	275-291	1
MAREANO	NA	4	Diastylidae	Diastylis lucifera	10.3252	58.3761	410-433	1
MAREANO	NA	4	Diastylidae	Diastylis lucifera	10.4435	58.4818	267-301	1
MAREANO	NA	4	Diastylidae	Diastylis lucifera	10.1333	58.4695	497-513	1
MAREANO	NA	4	Diastylidae	Diastylis lucifera	10.4391	58.8576	230-246	1
MAREANO	NA	4	Diastylidae	Diastylis lucifera	10.3230	58.7806	191-197	1
MAREANO	NA	4	Leuconidae	Eudorella emarginata	10.3759	58.6285	200-251	1
MAREANO	NA	4	Leuconidae	Eudorella hirsuta	10.1333	58.4695	497-513	1
MAREANO	NA	4	Leuconidae	Eudorella hirsuta	10.2242	58.6660	284-359	1
MAREANO	NA	4	Leuconidae	Eudorella truncatula	10.3230	58.7806	191-197	1
MAREANO	NA	4	Lampropidae	Hemilamprops cristatus	9.6472	58.4256	681-710	1
MAREANO	NA	4	Lampropidae	Hemilamprops roseus	10.5239	58.9236	170-230	1
MAREANO	NA	4	Diastylidae	Leptostylis longimana (G.O. Sars, 1865)	10.6061	58.9115	276-306	1
MAREANO	NA	4	Diastylidae	Leptostylis villosa	10.4184	58.5122	275-291	1
MAREANO	NA	4	Diastylidae	Leptostylis villosa	10.5239	58.9236	170-230	1
MAREANO	NA	4	Diastylidae	Leptostylis villosa	10.3230	58.7806	191-197	1
MAREANO	NA	4	Leuconidae	Leucon (Leucon) acutirostris	10.2242	58.6660	284-359	1
MAREANO	NA	4	Leuconidae	Leucon (Leucon) acutirostris	10.3851	58.4581	324-333	1
MAREANO	NA	4	Leuconidae	Leucon (Leucon) acutirostris	10.3759	58.6285	200-251	1
MAREANO	NA	4	Leuconidae	Leucon (Leucon) acutirostris	10.3232	58.5875	274-282	1
MAREANO	NA	4	Leuconidae	Leucon (Leucon) acutirostris	10.6061	58.9115	276-306	1
MAREANO	NA	4	Leuconidae	Leucon (Leucon) nasica	10.4184	58.5122	275-291	1
MAREANO	NA	4	Leuconidae	Leucon (Leucon) nasica	10.2242	58.6660	284-359	1
MAREANO	NA	4	Leuconidae	Leucon tener	10.1333	58.4695	497-513	1
MAREANO	NA	4	Leuconidae	Leucon tener	9.6472	58.4256	681-710	1
MAREANO	NA	5	Bodotriidae	Bodotria arenosa	2.7960	60.6010	108	1
MAREANO	NA	5	Nannastacidae	Campylaspis affinis	4.4130	63.2850	1260	1
MAREANO	NA	5	Nannastacidae	Campylaspis affinis	7.4260	66.0210	454	1
MAREANO	NA	5	Nannastacidae	Campylaspis affinis	8.8310	65.7160	438	1
MAREANO	NA	5	Nannastacidae	Campylaspis affinis	7.0430	65.6160	398	1
MAREANO	NA	5	Nannastacidae	Campylaspis affinis	7.0150	64.2580	343	1
MAREANO	NA	5	Nannastacidae	Campylaspis affinis	8.6960	64.2550	517	1
MAREANO	NA	5	Nannastacidae	Campylaspis affinis	2.0360	62.4710	502	1
MAREANO	NA	5	Nannastacidae	Campylaspis affinis	0.9810	62.5530	800	1
MAREANO	NA	5	Nannastacidae	Campylaspis affinis	2.1360	62.4950	497	1
MAREANO	NA	5	Nannastacidae	Campylaspis affinis	0.9810	62.5600	804	1
MAREANO	NA	5	Nannastacidae	Campylaspis affinis	3.2180	62.9850	804	1
MAREANO	NA	5	Nannastacidae	Campylaspis affinis	2.5150	66.6260	1626	1
MAREANO	NA	5	Nannastacidae	Campylaspis affinis	1.2330	62.5930	781	1
MAREANO	NA	5	Nannastacidae	Campylaspis affinis	1.9500	62.4300	473	1
MAREANO	NA	5	Nannastacidae	Campylaspis affinis	1.5400	62.4110	575	1
MAREANO	NA	5	Nannastacidae	Campylaspis affinis	1.4430	62.5250	701	1
MAREANO	NA	5	Nannastacidae	Campylaspis affinis	2.1310	62.4660	458	1
MAREANO	NA	5	Nannastacidae	Campylaspis affinis	1.7630	62.6660	746	1
MAREANO	NA	5	Nannastacidae	Campylaspis affinis	1.9030	67.2530	1595	1
MAREANO	NA	5	Nannastacidae	Campylaspis sulcata cf.	2.5100	61.7350	388	1
MAREANO	NA	5	Nannastacidae	Campylaspis costata	5.7830	63.0660	217	1
MAREANO	NA	5	Nannastacidae	Campylaspis costata	6.3380	63.0050	160	1

Supplemental Table xx Data source and station information on specimens incorporated in the distribution maps.

Data source	Station	Ecoregion	Taxon 1	Taxon 2	decLong	decLat	minDepth	Specimen count
MAREANO	NA	5	Nannastacidae	Campylaspis costata	6.4660	62.9780	147	1
MAREANO	NA	5	Nannastacidae	Campylaspis costata	5.7800	63.0950	210	1
MAREANO	NA	5	Nannastacidae	Campylaspis costata	6.4630	62.4680	201	1
MAREANO	NA	5	Nannastacidae	Campylaspis costata	5.8360	62.4680	258	1
MAREANO	NA	5	Nannastacidae	Campylaspis costata	13.0110	67.4860	264	1
MAREANO	NA	5	Nannastacidae	Campylaspis costata	7.0150	64.2580	343	1
MAREANO	NA	5	Nannastacidae	Campylaspis costata	7.0300	64.2400	341	1
MAREANO	NA	5	Nannastacidae	Campylaspis costata	5.7950	62.4730	258	1
MAREANO	NA	5	Nannastacidae	Campylaspis costata	3.0830	61.4930	400	1
MAREANO	NA	5	Nannastacidae	Campylaspis costata	4.5660	62.1230	225	1
MAREANO	NA	5	Nannastacidae	Campylaspis costata	4.4500	62.4730	216	1
MAREANO	NA	5	Nannastacidae	Campylaspis costata	4.4500	62.4730	216	1
MAREANO	NA	5	Nannastacidae	Campylaspis costata	6.2210	60.3780	63	1
MAREANO	NA	5	Nannastacidae	Campylaspis costata	6.2210	60.3780	63	1
MAREANO	NA	5	Nannastacidae	Campylaspis costata	6.2730	60.1410	204	1
MAREANO	NA	5	Nannastacidae	Campylaspis costata	5.9780	59.9700	166	1
MAREANO	NA	5	Nannastacidae	Campylaspis costata	3.4900	60.2550	291	1
MAREANO	NA	5	Nannastacidae	Campylaspis costata	5.4760	60.4560	220	1
MAREANO	NA	5	Nannastacidae	Campylaspis costata	5.2360	60.4850	315	1
MAREANO	NA	5	Nannastacidae	Campylaspis costata	2.0020	61.5000	311	1
MAREANO	NA	5	Nannastacidae	Campylaspis costata	2.5100	61.7350	388	1
MAREANO	NA	5	Nannastacidae	Campylaspis costata	3.5150	61.2510	374	1
MAREANO	NA	5	Nannastacidae	Campylaspis costata	3.9680	60.9950	343	1
MAREANO	NA	5	Nannastacidae	Campylaspis costata	5.5960	59.1760	557	1
MAREANO	NA	5	Nannastacidae	Campylaspis costata	4.9850	58.9910	234	1
MAREANO	NA	5	Nannastacidae	Campylaspis costata	2.7960	60.6010	108	1
MAREANO	NA	5	Nannastacidae	Campylaspis costata	2.8110	60.5780	106	1
MAREANO	NA	5	Nannastacidae	Campylaspis costata	2.9980	60.5030	118	1
MAREANO	NA	5	Nannastacidae	Campylaspis costata	2.7430	60.5000	104	1
MAREANO	NA	5	Nannastacidae	Campylaspis costata	5.4850	61.0250	116	1
MAREANO	NA	5	Nannastacidae	Campylaspis costata	5.4760	61.0210	101	1
MAREANO	NA	5	Nannastacidae	Campylaspis costata	1.9500	62.4300	473	1
MAREANO	NA	5	Nannastacidae	Campylaspis costata	2.5060	61.1010	209	1
MAREANO	NA	5	Nannastacidae	Campylaspis costata	2.3150	61.0030	135	1
MAREANO	NA	5	Nannastacidae	Campylaspis costata	2.5760	61.1260	255	1
MAREANO	NA	5	Nannastacidae	Campylaspis costata	5.4410	61.3800	147	1
MAREANO	NA	5	Nannastacidae	Campylaspis costata	5.4800	61.0230	121	1
MAREANO	NA	5	Nannastacidae	Campylaspis costata	4.0130	60.2360	297	1
MAREANO	NA	5	Nannastacidae	Campylaspis costata	21.0303	70.7015	258-260	1
MAREANO	NA	5	Nannastacidae	Campylaspis costata	21.5623	70.7518	308-312	1
MAREANO	NA	5	Nannastacidae	Campylaspis costata	20.8527	70.6773	195-199	1
MAREANO	NA	5	Nannastacidae	Campylaspis costata	11.8142	67.8468	183-173	1
MAREANO	NA	5	Nannastacidae	Campylaspis costata	10.1875	67.3982	224-223	1
MAREANO	NA	5	Nannastacidae	Campylaspis costata	7.8637	63.0348	100	1
MAREANO	NA	5	Nannastacidae	Campylaspis glabra	4.0000	60.1330	295	1
MAREANO	NA	5	Nannastacidae	Campylaspis glabra	6.0810	63.4150	272	1
MAREANO	NA	5	Nannastacidae	Campylaspis glabra	6.4660	62.9780	147	1
MAREANO	NA	5	Nannastacidae	Campylaspis glabra	13.0110	67.4860	264	1
MAREANO	NA	5	Nannastacidae	Campylaspis glabra	3.2180	62.9850	804	1
MAREANO	NA	5	Nannastacidae	Campylaspis glabra	2.5100	61.7350	388	1
MAREANO	NA	5	Nannastacidae	Campylaspis glabra	3.5150	61.2510	374	1
MAREANO	NA	5	Nannastacidae	Campylaspis glabra	5.4760	61.0210	101	1
MAREANO	NA	5	Nannastacidae	Campylaspis glabra	5.4800	61.0230	121	1
MAREANO	NA	5	Nannastacidae	Campylaspis glabra	1.9725	61.2670	145	1
MAREANO	NA	5	Nannastacidae	Campylaspis globosa	5.4160	61.0500	1250	1
MAREANO	NA	5	Nannastacidae	Campylaspis globosa	5.7830	63.0660	217	1
MAREANO	NA	5	Nannastacidae	Campylaspis globosa	6.3380	63.0050	160	1
MAREANO	NA	5	Nannastacidae	Campylaspis globosa	6.0810	63.4150	272	1
MAREANO	NA	5	Nannastacidae	Campylaspis globosa	6.4660	62.9780	147	1
MAREANO	NA	5	Nannastacidae	Campylaspis globosa	6.4630	62.4680	201	1
MAREANO	NA	5	Nannastacidae	Campylaspis globosa	5.8360	62.4680	258	1
MAREANO	NA	5	Nannastacidae	Campylaspis globosa	5.3160	61.9010	490	1
MAREANO	NA	5	Nannastacidae	Campylaspis globosa	5.4160	61.0460	1250	1
MAREANO	NA	5	Nannastacidae	Campylaspis globosa	13.0110	67.4860	264	1
MAREANO	NA	5	Nannastacidae	Campylaspis globosa	9.1280	67.1760	462	1
MAREANO	NA	5	Nannastacidae	Campylaspis globosa	5.6330	65.6860	602	1
MAREANO	NA	5	Nannastacidae	Campylaspis globosa	5.7950	62.4730	258	1
MAREANO	NA	5	Nannastacidae	Campylaspis globosa	5.7060	63.1200	192	1
MAREANO	NA	5	Nannastacidae	Campylaspis globosa	2.0360	62.4710	502	1
MAREANO	NA	5	Nannastacidae	Campylaspis globosa	3.8300	61.3230	368	1
MAREANO	NA	5	Nannastacidae	Campylaspis globosa	2.1360	62.4950	497	1
MAREANO	NA	5	Nannastacidae	Campylaspis globosa	1.7210	62.4910	604	1
MAREANO	NA	5	Nannastacidae	Campylaspis globosa	4.5660	62.1230	225	1
MAREANO	NA	5	Nannastacidae	Campylaspis globosa	4.4500	62.4730	216	1
MAREANO	NA	5	Nannastacidae	Campylaspis globosa	4.4500	62.4730	216	1
MAREANO	NA	5	Nannastacidae	Campylaspis globosa	5.4150	62.0480	1252	1
MAREANO	NA	5	Nannastacidae	Campylaspis globosa	6.2060	60.2980	835	1
MAREANO	NA	5	Nannastacidae	Campylaspis globosa	5.9080	60.0080	659	1
MAREANO	NA	5	Nannastacidae	Campylaspis globosa	3.4900	60.2550	291	1

Supplemental Table xx Data source and station information on specimens incorporated in the distribution maps.

Data source	Station	Ecoregion	Taxon 1	Taxon 2	decLong	decLat	minDepth	Specimen count
MAREANO	NA	5	Nannastacidae	Campylaspis globosa	2.0020	61.5000	311	1
MAREANO	NA	5	Nannastacidae	Campylaspis globosa	-0.0030	62.1980	708	1
MAREANO	NA	5	Nannastacidae	Campylaspis globosa	1.9500	62.4300	473	1
MAREANO	NA	5	Nannastacidae	Campylaspis globosa	1.5400	62.4110	575	1
MAREANO	NA	5	Nannastacidae	Campylaspis globosa	2.7000	61.1810	307	1
MAREANO	NA	5	Nannastacidae	Campylaspis globosa	2.7210	61.1930	315	1
MAREANO	NA	5	Nannastacidae	Campylaspis globosa	2.8360	61.2400	382	1
MAREANO	NA	5	Nannastacidae	Campylaspis globosa	21.5623	70.7518	308-312	1
MAREANO	NA	5	Nannastacidae	Campylaspis globosa	7.8637	63.0348	100	1
MAREANO	NA	5	Nannastacidae	Campylaspis horrida	5.8230	62.4700	264	1
MAREANO	NA	5	Nannastacidae	Campylaspis horrida	5.7830	63.0660	217	1
MAREANO	NA	5	Nannastacidae	Campylaspis horrida	6.3380	63.0050	160	1
MAREANO	NA	5	Nannastacidae	Campylaspis horrida	6.4660	62.9780	147	1
MAREANO	NA	5	Nannastacidae	Campylaspis horrida	6.4630	62.4680	201	1
MAREANO	NA	5	Nannastacidae	Campylaspis horrida	13.0110	67.4860	264	1
MAREANO	NA	5	Nannastacidae	Campylaspis horrida	9.1280	67.1760	462	1
MAREANO	NA	5	Nannastacidae	Campylaspis horrida	7.4260	66.0210	454	1
MAREANO	NA	5	Nannastacidae	Campylaspis horrida	8.8310	65.7160	438	1
MAREANO	NA	5	Nannastacidae	Campylaspis horrida	7.0430	65.6160	398	1
MAREANO	NA	5	Nannastacidae	Campylaspis horrida	4.0900	63.4230	1288	1
MAREANO	NA	5	Nannastacidae	Campylaspis horrida	2.0360	62.4710	502	1
MAREANO	NA	5	Nannastacidae	Campylaspis horrida	1.0430	62.8000	1009	1
MAREANO	NA	5	Nannastacidae	Campylaspis horrida	4.3530	60.8630	471	1
MAREANO	NA	5	Nannastacidae	Campylaspis horrida	3.8300	61.3230	368	1
MAREANO	NA	5	Nannastacidae	Campylaspis horrida	4.5660	62.1230	225	1
MAREANO	NA	5	Nannastacidae	Campylaspis horrida	2.9810	62.1650	400	1
MAREANO	NA	5	Nannastacidae	Campylaspis horrida	4.4500	62.4730	216	1
MAREANO	NA	5	Nannastacidae	Campylaspis horrida	4.4500	62.4730	216	1
MAREANO	NA	5	Nannastacidae	Campylaspis horrida	4.4510	62.4730	216	1
MAREANO	NA	5	Nannastacidae	Campylaspis horrida	2.0020	61.5000	311	1
MAREANO	NA	5	Nannastacidae	Campylaspis horrida	2.5100	61.7350	388	1
MAREANO	NA	5	Nannastacidae	Campylaspis horrida	3.5150	61.2510	374	1
MAREANO	NA	5	Nannastacidae	Campylaspis horrida	3.9680	60.9950	343	1
MAREANO	NA	5	Nannastacidae	Campylaspis horrida	1.8950	63.1330	1087	1
MAREANO	NA	5	Nannastacidae	Campylaspis horrida	2.0210	62.0020	374	1
MAREANO	NA	5	Nannastacidae	Campylaspis horrida	1.9500	62.4300	473	1
MAREANO	NA	5	Nannastacidae	Campylaspis horrida	1.5400	62.4110	575	1
MAREANO	NA	5	Nannastacidae	Campylaspis horrida	2.8260	61.2380	382	1
MAREANO	NA	5	Nannastacidae	Campylaspis horrida	2.7000	61.1810	307	1
MAREANO	NA	5	Nannastacidae	Campylaspis horrida	2.7210	61.1930	315	1
MAREANO	NA	5	Nannastacidae	Campylaspis horrida	2.8400	61.2430	383	1
MAREANO	NA	5	Nannastacidae	Campylaspis horrida	2.1310	62.4660	458	1
MAREANO	NA	5	Nannastacidae	Campylaspis horrida	4.0070	60.7410	315	1
MAREANO	NA	5	Nannastacidae	Campylaspis horrida	3.5100	60.7460	320	1
MAREANO	NA	5	Nannastacidae	Campylaspis horrida	3.5030	60.0020	270	1
MAREANO	NA	5	Nannastacidae	Campylaspis horrida	10.7732	67.6730	175-175	1
MAREANO	NA	5	Nannastacidae	Campylaspis horrida	21.5623	70.7518	308-312	1
MAREANO	NA	5	Nannastacidae	Campylaspis horrida	8.9230	67.0960	401-401	1
MAREANO	NA	5	Nannastacidae	Campylaspis horrida	20.1058	70.6262	289-293	1
MAREANO	NA	5	Nannastacidae	Campylaspis horrida	20.8527	70.6773	195-199	1
MAREANO	NA	5	Nannastacidae	Campylaspis intermedia	3.1210	63.2130	1003	1
MAREANO	NA	5	Nannastacidae	Campylaspis intermedia	1.2330	62.5930	781	1
MAREANO	NA	5	Nannastacidae	Campylaspis macrophthalma	2.0020	61.5000	311	1
MAREANO	NA	5	Nannastacidae	Campylaspis macrophthalma	2.5880	61.1280	257	1
MAREANO	NA	5	Nannastacidae	Campylaspis macrophthalma	2.5880	61.1280	260	1
MAREANO	NA	5	Nannastacidae	Campylaspis macrophthalma	2.5060	61.1010	209	1
MAREANO	NA	5	Nannastacidae	Campylaspis macrophthalma	2.3150	61.0030	135	1
MAREANO	NA	5	Nannastacidae	Campylaspis macrophthalma	2.5760	61.1260	255	1
MAREANO	NA	5	Nannastacidae	Campylaspis macrophthalma	1.9489	61.4414	284	1
MAREANO	NA	5	Nannastacidae	Campylaspis macrophthalma	2.1209	61.3954	295	1
MAREANO	NA	5	Nannastacidae	Campylaspis macrophthalma	2.1209	61.3836	287	1
MAREANO	NA	5	Nannastacidae	Campylaspis rubicunda	5.7830	63.0660	217	1
MAREANO	NA	5	Nannastacidae	Campylaspis rubicunda	6.3380	63.0050	160	1
MAREANO	NA	5	Nannastacidae	Campylaspis rubicunda	5.7800	63.0950	210	1
MAREANO	NA	5	Nannastacidae	Campylaspis rubicunda	6.4630	62.4680	201	1
MAREANO	NA	5	Nannastacidae	Campylaspis rubicunda	7.4260	66.0210	454	1
MAREANO	NA	5	Nannastacidae	Campylaspis rubicunda	3.1550	65.6460	1500	1
MAREANO	NA	5	Nannastacidae	Campylaspis rubicunda	7.0150	64.2580	343	1
MAREANO	NA	5	Nannastacidae	Campylaspis rubicunda	7.0300	64.2400	341	1
MAREANO	NA	5	Nannastacidae	Campylaspis rubicunda	5.7950	62.4730	258	1
MAREANO	NA	5	Nannastacidae	Campylaspis rubicunda	5.7060	63.1200	192	1
MAREANO	NA	5	Nannastacidae	Campylaspis rubicunda	1.0430	62.8000	1009	1
MAREANO	NA	5	Nannastacidae	Campylaspis rubicunda	0.9810	62.5530	800	1
MAREANO	NA	5	Nannastacidae	Campylaspis rubicunda	3.0830	61.4930	400	1
MAREANO	NA	5	Nannastacidae	Campylaspis rubicunda	3.2180	62.9850	804	1
MAREANO	NA	5	Nannastacidae	Campylaspis rubicunda	3.1210	63.2130	1003	1
MAREANO	NA	5	Nannastacidae	Campylaspis rubicunda	6.2210	60.3780	63	1
MAREANO	NA	5	Nannastacidae	Campylaspis rubicunda	6.2060	60.2980	835	1
MAREANO	NA	5	Nannastacidae	Campylaspis rubicunda	5.9780	59.9700	166	1

Supplemental Table xx Data source and station information on specimens incorporated in the distribution maps.

Data source	Station	Ecoregion	Taxon 1	Taxon 2	decLong	decLat	minDepth	Specimen count
MAREANO	NA	5	Nannastacidae	Campylaspis rubicunda	5.4760	60.4560	220	1
MAREANO	NA	5	Nannastacidae	Campylaspis rubicunda	6.4410	59.3160	110	1
MAREANO	NA	5	Nannastacidae	Campylaspis rubicunda	6.4410	59.3180	110	1
MAREANO	NA	5	Nannastacidae	Campylaspis rubicunda	2.7960	60.6010	108	1
MAREANO	NA	5	Nannastacidae	Campylaspis rubicunda	2.8110	60.5780	106	1
MAREANO	NA	5	Nannastacidae	Campylaspis rubicunda	2.9980	60.5030	118	1
MAREANO	NA	5	Nannastacidae	Campylaspis rubicunda	2.7430	60.5000	104	1
MAREANO	NA	5	Nannastacidae	Campylaspis rubicunda	1.2330	62.5930	781	1
MAREANO	NA	5	Nannastacidae	Campylaspis rubicunda	1.4430	62.5250	701	1
MAREANO	NA	5	Nannastacidae	Campylaspis rubicunda	2.4510	61.0730	174	1
MAREANO	NA	5	Nannastacidae	Campylaspis rubicunda	2.3150	61.0030	135	1
MAREANO	NA	5	Nannastacidae	Campylaspis rubicunda	2.5760	61.1260	255	1
MAREANO	NA	5	Nannastacidae	Campylaspis rubicunda	10.3562	68.1885	799-890	1
MAREANO	NA	5	Nannastacidae	Campylaspis rubicunda	21.0303	70.7015	258-260	1
MAREANO	NA	5	Nannastacidae	Campylaspis rubicunda	8.6419	67.3496	849-842	1
MAREANO	NA	5	Nannastacidae	Campylaspis rubicunda	20.8183	70.7703	246-247	1
MAREANO	NA	5	Nannastacidae	Campylaspis sulcata	4.0000	60.1330	295	1
MAREANO	NA	5	Nannastacidae	Campylaspis sulcata	5.8230	62.4700	264	1
MAREANO	NA	5	Nannastacidae	Campylaspis sulcata	5.7830	63.0660	217	1
MAREANO	NA	5	Nannastacidae	Campylaspis sulcata	6.3380	63.0050	160	1
MAREANO	NA	5	Nannastacidae	Campylaspis sulcata	6.4630	62.4680	201	1
MAREANO	NA	5	Nannastacidae	Campylaspis sulcata	13.0110	67.4860	264	1
MAREANO	NA	5	Nannastacidae	Campylaspis sulcata	9.1280	67.1760	462	1
MAREANO	NA	5	Nannastacidae	Campylaspis sulcata	7.4260	66.0210	454	1
MAREANO	NA	5	Nannastacidae	Campylaspis sulcata	8.8310	65.7160	438	1
MAREANO	NA	5	Nannastacidae	Campylaspis sulcata	7.0430	65.6160	398	1
MAREANO	NA	5	Nannastacidae	Campylaspis sulcata	5.6330	65.6860	602	1
MAREANO	NA	5	Nannastacidae	Campylaspis sulcata	8.1800	63.0600	480	1
MAREANO	NA	5	Nannastacidae	Campylaspis sulcata	5.7950	62.4730	258	1
MAREANO	NA	5	Nannastacidae	Campylaspis sulcata	0.6930	63.1980	1494	1
MAREANO	NA	5	Nannastacidae	Campylaspis sulcata	3.8300	61.3230	368	1
MAREANO	NA	5	Nannastacidae	Campylaspis sulcata	7.6600	61.2300	155	1
MAREANO	NA	5	Nannastacidae	Campylaspis sulcata	4.5660	62.1230	225	1
MAREANO	NA	5	Nannastacidae	Campylaspis sulcata	5.4680	61.9250	580	1
MAREANO	NA	5	Nannastacidae	Campylaspis sulcata	2.9810	62.1650	400	1
MAREANO	NA	5	Nannastacidae	Campylaspis sulcata	4.4500	62.4730	216	1
MAREANO	NA	5	Nannastacidae	Campylaspis sulcata	6.2730	60.1410	204	1
MAREANO	NA	5	Nannastacidae	Campylaspis sulcata	5.9080	60.0080	659	1
MAREANO	NA	5	Nannastacidae	Campylaspis sulcata	4.4910	59.2550	255	1
MAREANO	NA	5	Nannastacidae	Campylaspis sulcata	3.4900	60.2550	291	1
MAREANO	NA	5	Nannastacidae	Campylaspis sulcata	4.4710	60.2530	306	1
MAREANO	NA	5	Nannastacidae	Campylaspis sulcata	5.2360	60.4850	315	1
MAREANO	NA	5	Nannastacidae	Campylaspis sulcata	2.0020	61.5000	311	1
MAREANO	NA	5	Nannastacidae	Campylaspis sulcata	3.9680	60.9950	343	1
MAREANO	NA	5	Nannastacidae	Campylaspis sulcata	2.0210	62.0020	374	1
MAREANO	NA	5	Nannastacidae	Campylaspis sulcata	2.7000	61.1810	307	1
MAREANO	NA	5	Nannastacidae	Campylaspis sulcata	2.7210	61.1930	315	1
MAREANO	NA	5	Nannastacidae	Campylaspis sulcata	2.8360	61.2400	382	1
MAREANO	NA	5	Nannastacidae	Campylaspis sulcata	2.8400	61.2430	383	1
MAREANO	NA	5	Nannastacidae	Campylaspis sulcata	4.0070	60.7410	315	1
MAREANO	NA	5	Nannastacidae	Campylaspis sulcata	2.4687	61.3696	339	1
MAREANO	NA	5	Nannastacidae	Campylaspis sulcata	10.7732	67.6730	175-175	1
MAREANO	NA	5	Nannastacidae	Campylaspis sulcata	21.5623	70.7518	308-312	1
MAREANO	NA	5	Nannastacidae	Campylaspis sulcata	11.2240	68.0355	169-171	1
MAREANO	NA	5	Nannastacidae	Campylaspis sulcata	20.8527	70.6773	195-199	1
MAREANO	NA	5	Nannastacidae	Campylaspis undata	5.7830	63.0660	217	1
MAREANO	NA	5	Nannastacidae	Campylaspis undata	9.1280	67.1760	462	1
MAREANO	NA	5	Nannastacidae	Campylaspis undata	2.0360	62.4710	502	1
MAREANO	NA	5	Nannastacidae	Campylaspis undata	3.8300	61.3230	368	1
MAREANO	NA	5	Nannastacidae	Campylaspis undata	4.3530	60.8630	471	1
MAREANO	NA	5	Nannastacidae	Campylaspis undata	0.9810	62.5600	804	1
MAREANO	NA	5	Nannastacidae	Campylaspis undata	2.1310	62.4660	458	1
MAREANO	NA	5	Nannastacidae	Campylaspis undata	10.7732	67.6730	175-175	1
MAREANO	NA	5	Nannastacidae	Campylaspis undata	21.5623	70.7518	308-312	1
MAREANO	NA	5	Nannastacidae	Campylaspis undata	9.6169	67.5401	244-242	1
MAREANO	NA	5	Nannastacidae	Campylaspis undata	10.1875	67.3982	224-223	1
MAREANO	NA	5	Nannastacidae	Campylaspis undata	20.8527	70.6773	195-199	1
MAREANO	NA	5	Nannastacidae	Campylaspis	20.8183	70.7703	246-247	1
MAREANO	NA	5	Nannastacidae	Campylaspis	20.8183	70.7703	246-247	1
MAREANO	NA	5	Nannastacidae	Campylaspis	20.2323	70.7717	213-213	1
MAREANO	NA	5	Nannastacidae	Campylaspis	20.2323	70.7717	213-213	1
MAREANO	NA	5	Nannastacidae	Campylaspis	20.2323	70.7717	213-213	1
MAREANO	NA	5	Nannastacidae	Campylaspis verrucosa	6.2685	65.5006	NA	1
MAREANO	NA	5	Nannastacidae	Campylaspis verrucosa	5.4760	60.4560	220	1
MAREANO	NA	5	Nannastacidae	Campylaspis verrucosa	10.7732	67.6730	175-175	1
MAREANO	NA	5	Nannastacidae	Campylaspis verrucosa	4.4957	62.6143	203-203	1
MAREANO	NA	5	Nannastacidae	Campylaspis verrucosa	10.1875	67.3982	224-223	1
MAREANO	NA	5	Nannastacidae	Cumella (Cumella) pygmaea	4.5660	62.1230	225	1
MAREANO	NA	5	Nannastacidae	Cumella (Cumella) pygmaea	2.8110	60.5780	106	1

Supplemental Table xx Data source and station information on specimens incorporated in the distribution maps.

Data source	Station	Ecoregion	Taxon 1	Taxon 2	decLong	decLat	minDepth	Specimen count
MAREANO	NA	5	Nannastacidae	Cumella (Cumella) pygmaea	2.7430	60.5000	104	1
MAREANO	NA	5	Nannastacidae	Cumella (Cumella) pygmaea	20.8527	70.6773	195-199	1
MAREANO	NA	5	Nannastacidae	Cumella (Cumella) pygmaea	10.1875	67.3982	224-223	1
MAREANO	NA	5	Nannastacidae	Cumella (Cumella) pygmaea	11.8142	67.8468	183-173	1
MAREANO	NA	5	Nannastacidae	Cumella (Cumella) pygmaea	20.6858	70.6020	114-92	1
MAREANO	NA	5	Bodotriidae	Cyclaspis longicaudata	5.8230	62.4700	264	1
MAREANO	NA	5	Bodotriidae	Cyclaspis longicaudata	5.7830	63.0660	217	1
MAREANO	NA	5	Bodotriidae	Cyclaspis longicaudata	6.3380	63.0050	160	1
MAREANO	NA	5	Bodotriidae	Cyclaspis longicaudata	6.0810	63.4150	272	1
MAREANO	NA	5	Bodotriidae	Cyclaspis longicaudata	5.7800	63.0950	210	1
MAREANO	NA	5	Bodotriidae	Cyclaspis longicaudata	6.4630	62.4680	201	1
MAREANO	NA	5	Bodotriidae	Cyclaspis longicaudata	5.8360	62.4680	258	1
MAREANO	NA	5	Bodotriidae	Cyclaspis longicaudata	5.3160	61.9010	490	1
MAREANO	NA	5	Bodotriidae	Cyclaspis longicaudata	13.0110	67.4860	264	1
MAREANO	NA	5	Bodotriidae	Cyclaspis longicaudata	9.1280	67.1760	462	1
MAREANO	NA	5	Bodotriidae	Cyclaspis longicaudata	8.8310	65.7160	438	1
MAREANO	NA	5	Bodotriidae	Cyclaspis longicaudata	7.0430	65.6160	398	1
MAREANO	NA	5	Bodotriidae	Cyclaspis longicaudata	7.0150	64.2580	343	1
MAREANO	NA	5	Bodotriidae	Cyclaspis longicaudata	8.1800	63.0600	480	1
MAREANO	NA	5	Bodotriidae	Cyclaspis longicaudata	7.8800	63.1400	326	1
MAREANO	NA	5	Bodotriidae	Cyclaspis longicaudata	5.1160	61.4660	419	1
MAREANO	NA	5	Bodotriidae	Cyclaspis longicaudata	5.7950	62.4730	258	1
MAREANO	NA	5	Bodotriidae	Cyclaspis longicaudata	5.7060	63.1200	192	1
MAREANO	NA	5	Bodotriidae	Cyclaspis longicaudata	3.8300	61.3230	368	1
MAREANO	NA	5	Bodotriidae	Cyclaspis longicaudata	4.5660	62.1230	225	1
MAREANO	NA	5	Bodotriidae	Cyclaspis longicaudata	5.4210	61.9230	580	1
MAREANO	NA	5	Bodotriidae	Cyclaspis longicaudata	4.4500	62.4730	216	1
MAREANO	NA	5	Bodotriidae	Cyclaspis longicaudata	4.4910	59.2550	255	1
MAREANO	NA	5	Bodotriidae	Cyclaspis longicaudata	3.4900	60.2550	291	1
MAREANO	NA	5	Bodotriidae	Cyclaspis longicaudata	4.4710	60.2530	306	1
MAREANO	NA	5	Bodotriidae	Cyclaspis longicaudata	3.9680	60.9950	343	1
MAREANO	NA	5	Bodotriidae	Cyclaspis longicaudata	2.9900	61.2410	378	1
MAREANO	NA	5	Bodotriidae	Cyclaspis longicaudata	2.8260	61.2380	382	1
MAREANO	NA	5	Bodotriidae	Cyclaspis longicaudata	2.7000	61.1810	307	1
MAREANO	NA	5	Bodotriidae	Cyclaspis longicaudata	10.1875	67.3982	224-223	1
MAREANO	NA	5	Bodotriidae	Cyclaspis longicaudata	10.7732	67.6730	175-175	1
MAREANO	NA	5	Bodotriidae	Cyclaspis longicaudata	20.8527	70.6773	195-199	1
MAREANO	NA	5	Bodotriidae	Cyclaspis longicaudata	4.4957	62.6143	203-203	1
MAREANO	NA	5	Bodotriidae	Cyclaspis longicaudata	20.8183	70.7703	246-247	1
MAREANO	NA	5	Diastylidae	Diastylis cornuta	5.4160	61.0460	1250	1
MAREANO	NA	5	Diastylidae	Diastylis cornuta	5.8230	62.4700	264	1
MAREANO	NA	5	Diastylidae	Diastylis cornuta	6.0810	63.4150	272	1
MAREANO	NA	5	Diastylidae	Diastylis cornuta	6.4660	62.9780	147	1
MAREANO	NA	5	Diastylidae	Diastylis cornuta	5.3160	61.9010	490	1
MAREANO	NA	5	Diastylidae	Diastylis cornuta	13.0110	67.4860	264	1
MAREANO	NA	5	Diastylidae	Diastylis cornuta	7.4260	66.0210	454	1
MAREANO	NA	5	Diastylidae	Diastylis cornuta	7.0430	65.6160	398	1
MAREANO	NA	5	Diastylidae	Diastylis cornuta	7.0150	64.2580	343	1
MAREANO	NA	5	Diastylidae	Diastylis cornuta	7.0300	64.2400	341	1
MAREANO	NA	5	Diastylidae	Diastylis cornuta	8.1800	63.0600	480	1
MAREANO	NA	5	Diastylidae	Diastylis cornuta	7.8800	63.1400	326	1
MAREANO	NA	5	Diastylidae	Diastylis cornuta	5.4450	61.9230	584	1
MAREANO	NA	5	Diastylidae	Diastylis cornuta	5.7950	62.4730	258	1
MAREANO	NA	5	Diastylidae	Diastylis cornuta	6.8380	62.4450	679	1
MAREANO	NA	5	Diastylidae	Diastylis cornuta	3.0830	61.4930	400	1
MAREANO	NA	5	Diastylidae	Diastylis cornuta	7.0070	60.9710	298	1
MAREANO	NA	5	Diastylidae	Diastylis cornuta	6.6810	61.3100	217	1
MAREANO	NA	5	Diastylidae	Diastylis cornuta	6.5710	61.2280	296	1
MAREANO	NA	5	Diastylidae	Diastylis cornuta	7.6600	61.2300	155	1
MAREANO	NA	5	Diastylidae	Diastylis cornuta	7.3610	61.2450	648	1
MAREANO	NA	5	Diastylidae	Diastylis cornuta	4.9250	60.8580	460	1
MAREANO	NA	5	Diastylidae	Diastylis cornuta	4.5660	62.1230	225	1
MAREANO	NA	5	Diastylidae	Diastylis cornuta	4.4500	62.4730	216	1
MAREANO	NA	5	Diastylidae	Diastylis cornuta	4.4500	62.4730	216	1
MAREANO	NA	5	Diastylidae	Diastylis cornuta	4.4510	62.4730	216	1
MAREANO	NA	5	Diastylidae	Diastylis cornuta	6.2210	60.3780	63	1
MAREANO	NA	5	Diastylidae	Diastylis cornuta	6.2210	60.3780	63	1
MAREANO	NA	5	Diastylidae	Diastylis cornuta	6.2060	60.2980	835	1
MAREANO	NA	5	Diastylidae	Diastylis cornuta	6.2330	60.1180	271	1
MAREANO	NA	5	Diastylidae	Diastylis cornuta	6.2730	60.1410	204	1
MAREANO	NA	5	Diastylidae	Diastylis cornuta	5.9780	59.9700	166	1
MAREANO	NA	5	Diastylidae	Diastylis cornuta	4.4910	59.2550	255	1
MAREANO	NA	5	Diastylidae	Diastylis cornuta	3.4900	60.2550	291	1
MAREANO	NA	5	Diastylidae	Diastylis cornuta	4.4710	60.2530	306	1
MAREANO	NA	5	Diastylidae	Diastylis cornuta	5.4760	60.4560	220	1
MAREANO	NA	5	Diastylidae	Diastylis cornuta	5.2360	60.4850	315	1
MAREANO	NA	5	Diastylidae	Diastylis cornuta	2.0020	61.5000	311	1
MAREANO	NA	5	Diastylidae	Diastylis cornuta	2.5100	61.7350	388	1
MAREANO	NA	5	Diastylidae	Diastylis cornuta	3.5150	61.2510	374	1

Supplemental Table xx Data source and station information on specimens incorporated in the distribution maps.

Data source	Station	Ecoregion	Taxon 1	Taxon 2	decLong	decLat	minDepth	Specimen count
MAREANO	NA	5	Diastylidae	Diastylis cornuta	5.5960	59.1760	557	1
MAREANO	NA	5	Diastylidae	Diastylis cornuta	6.4410	59.3180	110	1
MAREANO	NA	5	Diastylidae	Diastylis cornuta	4.9850	58.9910	234	1
MAREANO	NA	5	Diastylidae	Diastylis cornuta	5.4850	61.0250	116	1
MAREANO	NA	5	Diastylidae	Diastylis cornuta	2.9900	61.2410	378	1
MAREANO	NA	5	Diastylidae	Diastylis cornuta	6.7360	61.3550	181	1
MAREANO	NA	5	Diastylidae	Diastylis cornuta	6.5700	61.2350	294	1
MAREANO	NA	5	Diastylidae	Diastylis cornuta	6.0550	62.4030	449	1
MAREANO	NA	5	Diastylidae	Diastylis cornuta	2.0210	62.0020	374	1
MAREANO	NA	5	Diastylidae	Diastylis cornuta	6.0480	61.1860	254	1
MAREANO	NA	5	Diastylidae	Diastylis cornuta	5.4760	61.0210	101	1
MAREANO	NA	5	Diastylidae	Diastylis cornuta	2.8260	61.2380	382	1
MAREANO	NA	5	Diastylidae	Diastylis cornuta	2.7000	61.1810	307	1
MAREANO	NA	5	Diastylidae	Diastylis cornuta	2.5880	61.1280	257	1
MAREANO	NA	5	Diastylidae	Diastylis cornuta	2.5880	61.1280	260	1
MAREANO	NA	5	Diastylidae	Diastylis cornuta	2.5830	61.1280	257	1
MAREANO	NA	5	Diastylidae	Diastylis cornuta	2.5760	61.1260	255	1
MAREANO	NA	5	Diastylidae	Diastylis cornuta	2.8360	61.2400	382	1
MAREANO	NA	5	Diastylidae	Diastylis cornuta	2.8400	61.2430	383	1
MAREANO	NA	5	Diastylidae	Diastylis cornuta	5.5730	60.8710	37	1
MAREANO	NA	5	Diastylidae	Diastylis cornuta	7.5950	62.6980	360	1
MAREANO	NA	5	Diastylidae	Diastylis cornuta	5.4410	61.3800	147	1
MAREANO	NA	5	Diastylidae	Diastylis cornuta	4.0070	60.7410	315	1
MAREANO	NA	5	Diastylidae	Diastylis cornuta	3.5100	60.7460	320	1
MAREANO	NA	5	Diastylidae	Diastylis cornuta	4.0130	60.2360	297	1
MAREANO	NA	5	Diastylidae	Diastylis cornuta	10.7732	67.6730	175-175	1
MAREANO	NA	5	Diastylidae	Diastylis cornuta	20.1058	70.6262	289-293	1
MAREANO	NA	5	Diastylidae	Diastylis cornuta	21.5623	70.7518	308-312	1
MAREANO	NA	5	Diastylidae	Diastylis cornuta	20.8183	70.7703	246-247	1
MAREANO	NA	5	Diastylidae	Diastylis cornuta	20.8527	70.6773	195-199	1
MAREANO	NA	5	Diastylidae	Diastylis echinata	5.8230	62.4700	264	1
MAREANO	NA	5	Diastylidae	Diastylis echinata	6.3380	63.0050	160	1
MAREANO	NA	5	Diastylidae	Diastylis echinata	13.0110	67.4860	264	1
MAREANO	NA	5	Diastylidae	Diastylis echinata	7.4260	66.0210	454	1
MAREANO	NA	5	Diastylidae	Diastylis echinata	8.8310	65.7160	438	1
MAREANO	NA	5	Diastylidae	Diastylis echinata	7.0150	64.2580	343	1
MAREANO	NA	5	Diastylidae	Diastylis echinata	5.3160	61.9010	490	1
MAREANO	NA	5	Diastylidae	Diastylis echinata	5.1160	61.4660	419	1
MAREANO	NA	5	Diastylidae	Diastylis echinata	5.7060	63.1200	192	1
MAREANO	NA	5	Diastylidae	Diastylis echinata	3.0830	61.4930	400	1
MAREANO	NA	5	Diastylidae	Diastylis echinata	3.8300	61.3230	368	1
MAREANO	NA	5	Diastylidae	Diastylis echinata	1.7210	62.4910	604	1
MAREANO	NA	5	Diastylidae	Diastylis echinata	0.9810	62.5600	804	1
MAREANO	NA	5	Diastylidae	Diastylis echinata	4.4500	62.4730	216	1
MAREANO	NA	5	Diastylidae	Diastylis echinata	4.4910	59.2550	255	1
MAREANO	NA	5	Diastylidae	Diastylis echinata	3.4900	60.2550	291	1
MAREANO	NA	5	Diastylidae	Diastylis echinata	4.4710	60.2530	306	1
MAREANO	NA	5	Diastylidae	Diastylis echinata	3.5150	61.2510	374	1
MAREANO	NA	5	Diastylidae	Diastylis echinata	3.9680	60.9950	343	1
MAREANO	NA	5	Diastylidae	Diastylis echinata	4.9850	58.9910	234	1
MAREANO	NA	5	Diastylidae	Diastylis echinata	2.9900	61.2410	378	1
MAREANO	NA	5	Diastylidae	Diastylis echinata	1.2330	62.5930	781	1
MAREANO	NA	5	Diastylidae	Diastylis echinata	-0.0030	62.1980	708	1
MAREANO	NA	5	Diastylidae	Diastylis echinata	1.8200	62.5530	625	1
MAREANO	NA	5	Diastylidae	Diastylis echinata	2.0210	62.0020	374	1
MAREANO	NA	5	Diastylidae	Diastylis echinata	1.9500	62.4300	473	1
MAREANO	NA	5	Diastylidae	Diastylis echinata	1.4430	62.5250	701	1
MAREANO	NA	5	Diastylidae	Diastylis echinata	1.1860	62.7060	897	1
MAREANO	NA	5	Diastylidae	Diastylis echinata	2.8260	61.2380	382	1
MAREANO	NA	5	Diastylidae	Diastylis echinata	2.7000	61.1810	307	1
MAREANO	NA	5	Diastylidae	Diastylis echinata	2.1310	62.4660	458	1
MAREANO	NA	5	Diastylidae	Diastylis echinata	4.0070	60.7410	315	1
MAREANO	NA	5	Diastylidae	Diastylis echinata	4.0130	60.2360	297	1
MAREANO	NA	5	Diastylidae	Diastylis echinata	10.7732	67.6730	175-175	1
MAREANO	NA	5	Diastylidae	Diastylis echinata	21.5623	70.7518	308-312	1
MAREANO	NA	5	Diastylidae	Diastylis echinata	20.8183	70.7703	246-247	1
MAREANO	NA	5	Diastylidae	Diastylis echinata	20.8183	70.7703	246-247	1
MAREANO	NA	5	Diastylidae	Diastylis polaris	4.4130	63.2850	1260	1
MAREANO	NA	5	Diastylidae	Diastylis polaris	4.8150	65.6660	996	1
MAREANO	NA	5	Diastylidae	Diastylis polaris	1.7210	62.4910	604	1
MAREANO	NA	5	Diastylidae	Diastylis polaris	2.5150	66.6260	1626	1
MAREANO	NA	5	Diastylidae	Diastylis polaris	3.1210	63.2130	1003	1
MAREANO	NA	5	Diastylidae	Diastylis polaris	-0.0030	62.1980	708	1
MAREANO	NA	5	Diastylidae	Diastylis polaris	1.8950	63.1330	1087	1
MAREANO	NA	5	Diastylidae	Diastylis polaris	1.8200	62.5530	625	1
MAREANO	NA	5	Diastylidae	Diastylis polaris	1.4430	62.5250	701	1
MAREANO	NA	5	Diastylidae	Diastylis polaris	0.9280	62.9110	1112	1
MAREANO	NA	5	Diastylidae	Diastylis polaris	0.4710	63.2910	1698	1
MAREANO	NA	5	Diastylidae	Diastylis rathkei	13.0110	67.4860	264	1

Supplemental Table xx Data source and station information on specimens incorporated in the distribution maps.

Data source	Station	Ecoregion	Taxon 1	Taxon 2	decLong	decLat	minDepth	Specimen count
MAREANO	NA	5	Diastylidae	Diastylis rathkei	4.8150	65.6660	996	1
MAREANO	NA	5	Diastylidae	Diastylis rathkei	1.0430	62.8000	1009	1
MAREANO	NA	5	Diastylidae	Diastylis rathkei	1.0450	62.8060	1003	1
MAREANO	NA	5	Diastylidae	Diastylis rathkei	2.7650	63.1780	1030	1
MAREANO	NA	5	Diastylidae	Diastylis rathkei	3.2180	62.9850	804	1
MAREANO	NA	5	Diastylidae	Diastylis rathkei	1.8200	62.5530	625	1
MAREANO	NA	5	Diastylidae	Diastylis rathkei	1.4430	62.5250	701	1
MAREANO	NA	5	Diastylidae	Diastylis rathkei	1.1860	62.7060	897	1
MAREANO	NA	5	Diastylidae	Diastylis rathkei	0.9280	62.9110	1112	1
MAREANO	NA	5	Diastylidae	Diastylis rathkei	1.7630	62.6660	746	1
MAREANO	NA	5	Diastylidae	Diastylis rathkei	10.7732	67.6730	175-175	1
MAREANO	NA	5	Diastylidae	Diastylis rathkei	10.7732	67.6730	175-175	1
MAREANO	NA	5	Diastylidae	Diastylis rugosa	5.9080	60.0080	659	1
MAREANO	NA	5	Diastylidae	Diastylis spinulosa	4.0000	60.1330	295	1
MAREANO	NA	5	Diastylidae	Diastylis stygia	-1.5500	64.8030	3000	1
MAREANO	NA	5	Diastylidae	Diastylis stygia	0.8080	63.0480	1286	1
MAREANO	NA	5	Diastylidae	Diastylis stygia	-0.4980	64.1330	2497	1
MAREANO	NA	5	Diastylidae	Diastylis stygia	-0.3950	63.9700	2481	1
MAREANO	NA	5	Diastylidae	Diastylis stygia	-0.1610	63.7110	2259	1
MAREANO	NA	5	Diastylidae	Diastylis stygia	7.7565	73.5890	2452	1
MAREANO	NA	5	Diastylidae	Diastylis stygia	9.2487	68.2468	2346-2338	1
MAREANO	NA	5	Diastylidae	Diastylis stygia	0.4710	63.2910	1698	1
MAREANO	NA	5	Diastylidae	Diastylis stygia	7.7565	73.5890	2452	1
MAREANO	NA	5	Diastylidae	Diastylis stygia	7.7565	73.5890	2452	1
MAREANO	NA	5	Diastylidae	Diastylis tumida	7.8800	63.1400	326	1
MAREANO	NA	5	Diastylidae	Diastylis tumida	4.4500	62.4730	216	1
MAREANO	NA	5	Diastylidae	Diastylis tumida	5.9310	60.1300	499	1
MAREANO	NA	5	Diastylidae	Diastylis tumida	6.0930	60.2350	649	1
MAREANO	NA	5	Diastylidae	Diastylis tumida	6.2060	60.2980	835	1
MAREANO	NA	5	Diastylidae	Diastylis tumida	5.9080	60.0080	659	1
MAREANO	NA	5	Diastylidae	Diastylis tumida	9.6169	67.5401	244-242	1
MAREANO	NA	5	Diastylidae	Diastylis tumida	11.8142	67.8468	183-173	1
MAREANO	NA	5	Diastylidae	Diastylis tumida	11.2240	68.0355	169-171	1
MAREANO	NA	5	Diastylidae	Diastyloides biplicatus	5.8230	62.4700	264	1
MAREANO	NA	5	Diastylidae	Diastyloides biplicatus	5.7830	63.0660	217	1
MAREANO	NA	5	Diastylidae	Diastyloides biplicatus	6.3380	63.0050	160	1
MAREANO	NA	5	Diastylidae	Diastyloides biplicatus	6.4660	62.9780	147	1
MAREANO	NA	5	Diastylidae	Diastyloides biplicatus	5.7800	63.0950	210	1
MAREANO	NA	5	Diastylidae	Diastyloides biplicatus	6.0450	62.4150	449	1
MAREANO	NA	5	Diastylidae	Diastyloides biplicatus	6.4630	62.4680	201	1
MAREANO	NA	5	Diastylidae	Diastyloides biplicatus	5.8360	62.4680	258	1
MAREANO	NA	5	Diastylidae	Diastyloides biplicatus	5.3160	61.9010	490	1
MAREANO	NA	5	Diastylidae	Diastyloides biplicatus	13.0110	67.4860	264	1
MAREANO	NA	5	Diastylidae	Diastyloides biplicatus	7.4260	66.0210	454	1
MAREANO	NA	5	Diastylidae	Diastyloides biplicatus	8.8310	65.7160	438	1
MAREANO	NA	5	Diastylidae	Diastyloides biplicatus	7.0430	65.6160	398	1
MAREANO	NA	5	Diastylidae	Diastyloides biplicatus	7.0150	64.2580	343	1
MAREANO	NA	5	Diastylidae	Diastyloides biplicatus	7.0300	64.2400	341	1
MAREANO	NA	5	Diastylidae	Diastyloides biplicatus	8.6960	64.2550	517	1
MAREANO	NA	5	Diastylidae	Diastyloides biplicatus	8.1800	63.0600	480	1
MAREANO	NA	5	Diastylidae	Diastyloides biplicatus	7.8800	63.1400	326	1
MAREANO	NA	5	Diastylidae	Diastyloides biplicatus	4.8730	60.8450	460	1
MAREANO	NA	5	Diastylidae	Diastyloides biplicatus	5.1160	61.4660	419	1
MAREANO	NA	5	Diastylidae	Diastyloides biplicatus	5.7950	62.4730	258	1
MAREANO	NA	5	Diastylidae	Diastyloides biplicatus	5.7060	63.1200	192	1
MAREANO	NA	5	Diastylidae	Diastyloides biplicatus	6.0260	62.4210	445	1
MAREANO	NA	5	Diastylidae	Diastyloides biplicatus	3.0830	61.4930	400	1
MAREANO	NA	5	Diastylidae	Diastyloides biplicatus	5.8080	61.1850	230	1
MAREANO	NA	5	Diastylidae	Diastyloides biplicatus	7.0070	60.9710	298	1
MAREANO	NA	5	Diastylidae	Diastyloides biplicatus	7.0460	61.0150	506	1
MAREANO	NA	5	Diastylidae	Diastyloides biplicatus	7.6600	61.2300	155	1
MAREANO	NA	5	Diastylidae	Diastyloides biplicatus	7.5750	61.2010	277	1
MAREANO	NA	5	Diastylidae	Diastyloides biplicatus	7.6600	61.2300	155	1
MAREANO	NA	5	Diastylidae	Diastyloides biplicatus	4.9250	60.8580	460	1
MAREANO	NA	5	Diastylidae	Diastyloides biplicatus	4.5660	62.1230	225	1
MAREANO	NA	5	Diastylidae	Diastyloides biplicatus	5.4210	61.9230	580	1
MAREANO	NA	5	Diastylidae	Diastyloides biplicatus	5.1200	61.4680	420	1
MAREANO	NA	5	Diastylidae	Diastyloides biplicatus	5.1200	61.4680	420	1
MAREANO	NA	5	Diastylidae	Diastyloides biplicatus	6.0700	62.3910	447	1
MAREANO	NA	5	Diastylidae	Diastyloides biplicatus	4.4500	62.4730	216	1
MAREANO	NA	5	Diastylidae	Diastyloides biplicatus	4.4500	62.4730	216	1
MAREANO	NA	5	Diastylidae	Diastyloides biplicatus	6.2210	60.3780	63	1
MAREANO	NA	5	Diastylidae	Diastyloides biplicatus	6.2210	60.3780	63	1
MAREANO	NA	5	Diastylidae	Diastyloides biplicatus	6.2060	60.2980	835	1
MAREANO	NA	5	Diastylidae	Diastyloides biplicatus	6.2330	60.1180	271	1
MAREANO	NA	5	Diastylidae	Diastyloides biplicatus	6.2730	60.1410	204	1
MAREANO	NA	5	Diastylidae	Diastyloides biplicatus	5.9780	59.9700	166	1
MAREANO	NA	5	Diastylidae	Diastyloides biplicatus	5.4760	60.4560	220	1
MAREANO	NA	5	Diastylidae	Diastyloides biplicatus	5.2360	60.4850	315	1

Supplemental Table xx Data source and station information on specimens incorporated in the distribution maps.

Data source	Station	Ecoregion	Taxon 1	Taxon 2	decLong	decLat	minDepth	Specimen count
MAREANO	NA	5	Diastylidae	Diastyloides biplicatus	2.0020	61.5000	311	1
MAREANO	NA	5	Diastylidae	Diastyloides biplicatus	5.5960	59.1760	557	1
MAREANO	NA	5	Diastylidae	Diastyloides biplicatus	6.4410	59.3160	110	1
MAREANO	NA	5	Diastylidae	Diastyloides biplicatus	6.4410	59.3180	110	1
MAREANO	NA	5	Diastylidae	Diastyloides biplicatus	4.9850	58.9910	234	1
MAREANO	NA	5	Diastylidae	Diastyloides biplicatus	5.9700	59.8080	286	1
MAREANO	NA	5	Diastylidae	Diastyloides biplicatus	5.5900	59.7600	368	1
MAREANO	NA	5	Diastylidae	Diastyloides biplicatus	2.7960	60.6010	108	1
MAREANO	NA	5	Diastylidae	Diastyloides biplicatus	2.9980	60.5030	118	1
MAREANO	NA	5	Diastylidae	Diastyloides biplicatus	2.7430	60.5000	104	1
MAREANO	NA	5	Diastylidae	Diastyloides biplicatus	5.4850	61.0250	116	1
MAREANO	NA	5	Diastylidae	Diastyloides biplicatus	2.9900	61.2410	378	1
MAREANO	NA	5	Diastylidae	Diastyloides biplicatus	6.7360	61.3550	181	1
MAREANO	NA	5	Diastylidae	Diastyloides biplicatus	6.5700	61.2350	294	1
MAREANO	NA	5	Diastylidae	Diastyloides biplicatus	5.9650	61.7800	145	1
MAREANO	NA	5	Diastylidae	Diastyloides biplicatus	6.0550	62.4030	449	1
MAREANO	NA	5	Diastylidae	Diastyloides biplicatus	6.2360	61.1810	40	1
MAREANO	NA	5	Diastylidae	Diastyloides biplicatus	5.8330	61.0910	160	1
MAREANO	NA	5	Diastylidae	Diastyloides biplicatus	5.4760	61.0210	101	1
MAREANO	NA	5	Diastylidae	Diastyloides biplicatus	1.5400	62.4110	575	1
MAREANO	NA	5	Diastylidae	Diastyloides biplicatus	2.7000	61.1810	307	1
MAREANO	NA	5	Diastylidae	Diastyloides biplicatus	2.5880	61.1280	257	1
MAREANO	NA	5	Diastylidae	Diastyloides biplicatus	2.5880	61.1280	260	1
MAREANO	NA	5	Diastylidae	Diastyloides biplicatus	2.5130	61.1000	211	1
MAREANO	NA	5	Diastylidae	Diastyloides biplicatus	2.5830	61.1280	257	1
MAREANO	NA	5	Diastylidae	Diastyloides biplicatus	2.5760	61.1260	255	1
MAREANO	NA	5	Diastylidae	Diastyloides biplicatus	5.5060	62.0610	150	1
MAREANO	NA	5	Diastylidae	Diastyloides biplicatus	4.9830	60.6250	410	1
MAREANO	NA	5	Diastylidae	Diastyloides biplicatus	5.4800	61.0230	121	1
MAREANO	NA	5	Diastylidae	Diastyloides biplicatus	10.7732	67.6730	175-175	1
MAREANO	NA	5	Diastylidae	Diastyloides biplicatus	11.8142	67.8468	183-173	1
MAREANO	NA	5	Diastylidae	Diastyloides biplicatus	4.4957	62.6143	203-203	1
MAREANO	NA	5	Diastylidae	Diastyloides biplicatus	21.0303	70.7015	258-260	1
MAREANO	NA	5	Diastylidae	Diastyloides biplicatus	8.9230	67.0960	401-401	1
MAREANO	NA	5	Diastylidae	Diastyloides biplicatus	10.1875	67.3982	224-223	1
MAREANO	NA	5	Diastylidae	Diastyloides serratus	5.4160	61.0500	1250	1
MAREANO	NA	5	Diastylidae	Diastyloides serratus	6.5850	61.1760	1100	1
MAREANO	NA	5	Diastylidae	Diastyloides serratus	4.8730	60.8450	470	1
MAREANO	NA	5	Diastylidae	Diastyloides serratus	5.0530	60.8300	549	1
MAREANO	NA	5	Diastylidae	Diastyloides serratus	4.0000	60.1330	295	1
MAREANO	NA	5	Diastylidae	Diastyloides serratus	5.4160	61.0460	1250	1
MAREANO	NA	5	Diastylidae	Diastyloides serratus	5.8230	62.4700	264	1
MAREANO	NA	5	Diastylidae	Diastyloides serratus	6.3380	63.0050	160	1
MAREANO	NA	5	Diastylidae	Diastyloides serratus	6.4630	62.4680	201	1
MAREANO	NA	5	Diastylidae	Diastyloides serratus	5.9610	62.1730	701	1
MAREANO	NA	5	Diastylidae	Diastyloides serratus	5.3160	61.9010	490	1
MAREANO	NA	5	Diastylidae	Diastyloides serratus	5.0380	61.7880	380	1
MAREANO	NA	5	Diastylidae	Diastyloides serratus	5.4160	61.0460	1250	1
MAREANO	NA	5	Diastylidae	Diastyloides serratus	7.4260	66.0210	454	1
MAREANO	NA	5	Diastylidae	Diastyloides serratus	8.8310	65.7160	438	1
MAREANO	NA	5	Diastylidae	Diastyloides serratus	7.0430	65.6160	398	1
MAREANO	NA	5	Diastylidae	Diastyloides serratus	7.0150	64.2580	343	1
MAREANO	NA	5	Diastylidae	Diastyloides serratus	7.0300	64.2400	341	1
MAREANO	NA	5	Diastylidae	Diastyloides serratus	8.1800	63.0600	480	1
MAREANO	NA	5	Diastylidae	Diastyloides serratus	7.8800	63.1400	326	1
MAREANO	NA	5	Diastylidae	Diastyloides serratus	4.8730	60.8450	460	1
MAREANO	NA	5	Diastylidae	Diastyloides serratus	5.4160	61.0460	1256	1
MAREANO	NA	5	Diastylidae	Diastyloides serratus	5.1160	61.4660	419	1
MAREANO	NA	5	Diastylidae	Diastyloides serratus	5.4450	61.9230	584	1
MAREANO	NA	5	Diastylidae	Diastyloides serratus	5.9950	62.1700	701	1
MAREANO	NA	5	Diastylidae	Diastyloides serratus	5.4450	61.9230	584	1
MAREANO	NA	5	Diastylidae	Diastyloides serratus	5.7950	62.4730	258	1
MAREANO	NA	5	Diastylidae	Diastyloides serratus	6.8380	62.4450	679	1
MAREANO	NA	5	Diastylidae	Diastyloides serratus	6.0260	62.4210	445	1
MAREANO	NA	5	Diastylidae	Diastyloides serratus	3.0830	61.4930	400	1
MAREANO	NA	5	Diastylidae	Diastyloides serratus	5.7680	61.1430	1260	1
MAREANO	NA	5	Diastylidae	Diastyloides serratus	6.1510	61.1380	1235	1
MAREANO	NA	5	Diastylidae	Diastyloides serratus	5.8080	61.1850	230	1
MAREANO	NA	5	Diastylidae	Diastyloides serratus	7.1000	61.2080	260	1
MAREANO	NA	5	Diastylidae	Diastyloides serratus	7.0070	60.9710	298	1
MAREANO	NA	5	Diastylidae	Diastyloides serratus	7.0460	61.0150	506	1
MAREANO	NA	5	Diastylidae	Diastyloides serratus	6.6810	61.3100	217	1
MAREANO	NA	5	Diastylidae	Diastyloides serratus	6.5710	61.2280	296	1
MAREANO	NA	5	Diastylidae	Diastyloides serratus	7.6600	61.2300	155	1
MAREANO	NA	5	Diastylidae	Diastyloides serratus	7.5750	61.2010	277	1
MAREANO	NA	5	Diastylidae	Diastyloides serratus	7.3660	61.3450	373	1
MAREANO	NA	5	Diastylidae	Diastyloides serratus	6.9030	61.1230	860	1
MAREANO	NA	5	Diastylidae	Diastyloides serratus	7.6600	61.2300	155	1
MAREANO	NA	5	Diastylidae	Diastyloides serratus	7.1480	61.1280	935	1

Supplemental Table xx Data source and station information on specimens incorporated in the distribution maps.

Data source	Station	Ecoregion	Taxon 1	Taxon 2	decLong	decLat	minDepth	Specimen count
MAREANO	NA	5	Diastylidae	Diastyloides serratus	7.3610	61.2450	648	1
MAREANO	NA	5	Diastylidae	Diastyloides serratus	4.9250	60.8580	460	1
MAREANO	NA	5	Diastylidae	Diastyloides serratus	4.5660	62.1230	225	1
MAREANO	NA	5	Diastylidae	Diastyloides serratus	5.4210	61.9230	580	1
MAREANO	NA	5	Diastylidae	Diastyloides serratus	5.1200	61.4680	420	1
MAREANO	NA	5	Diastylidae	Diastyloides serratus	5.1200	61.4680	420	1
MAREANO	NA	5	Diastylidae	Diastyloides serratus	5.3750	61.0560	1250	1
MAREANO	NA	5	Diastylidae	Diastyloides serratus	5.4680	61.9250	580	1
MAREANO	NA	5	Diastylidae	Diastyloides serratus	6.0700	62.3910	447	1
MAREANO	NA	5	Diastylidae	Diastyloides serratus	2.9810	62.1650	400	1
MAREANO	NA	5	Diastylidae	Diastyloides serratus	5.4150	62.0480	1252	1
MAREANO	NA	5	Diastylidae	Diastyloides serratus	5.9310	60.1300	499	1
MAREANO	NA	5	Diastylidae	Diastyloides serratus	6.0930	60.2350	649	1
MAREANO	NA	5	Diastylidae	Diastyloides serratus	6.9510	60.5380	254	1
MAREANO	NA	5	Diastylidae	Diastyloides serratus	7.0020	60.4960	388	1
MAREANO	NA	5	Diastylidae	Diastyloides serratus	6.7810	60.4610	534	1
MAREANO	NA	5	Diastylidae	Diastyloides serratus	6.5930	60.2450	386	1
MAREANO	NA	5	Diastylidae	Diastyloides serratus	6.3100	60.3600	855	1
MAREANO	NA	5	Diastylidae	Diastyloides serratus	6.2210	60.3780	63	1
MAREANO	NA	5	Diastylidae	Diastyloides serratus	6.2210	60.3780	63	1
MAREANO	NA	5	Diastylidae	Diastyloides serratus	6.2060	60.2980	835	1
MAREANO	NA	5	Diastylidae	Diastyloides serratus	5.8830	59.7480	340	1
MAREANO	NA	5	Diastylidae	Diastyloides serratus	6.2330	60.1180	271	1
MAREANO	NA	5	Diastylidae	Diastyloides serratus	6.2730	60.1410	204	1
MAREANO	NA	5	Diastylidae	Diastyloides serratus	5.9080	60.0080	659	1
MAREANO	NA	5	Diastylidae	Diastyloides serratus	5.9780	59.9700	166	1
MAREANO	NA	5	Diastylidae	Diastyloides serratus	4.4910	59.2550	255	1
MAREANO	NA	5	Diastylidae	Diastyloides serratus	3.4900	60.2550	291	1
MAREANO	NA	5	Diastylidae	Diastyloides serratus	4.4710	60.2530	306	1
MAREANO	NA	5	Diastylidae	Diastyloides serratus	5.4760	60.4560	220	1
MAREANO	NA	5	Diastylidae	Diastyloides serratus	5.3950	60.5710	591	1
MAREANO	NA	5	Diastylidae	Diastyloides serratus	5.2360	60.4850	315	1
MAREANO	NA	5	Diastylidae	Diastyloides serratus	5.2750	60.4230	310	1
MAREANO	NA	5	Diastylidae	Diastyloides serratus	2.0020	61.5000	311	1
MAREANO	NA	5	Diastylidae	Diastyloides serratus	3.5150	61.2510	374	1
MAREANO	NA	5	Diastylidae	Diastyloides serratus	3.9680	60.9950	343	1
MAREANO	NA	5	Diastylidae	Diastyloides serratus	5.5960	59.1760	557	1
MAREANO	NA	5	Diastylidae	Diastyloides serratus	5.8060	59.3050	700	1
MAREANO	NA	5	Diastylidae	Diastyloides serratus	6.2410	59.5000	420	1
MAREANO	NA	5	Diastylidae	Diastyloides serratus	6.2160	59.3160	315	1
MAREANO	NA	5	Diastylidae	Diastyloides serratus	6.2410	59.5000	404	1
MAREANO	NA	5	Diastylidae	Diastyloides serratus	6.4410	59.3180	110	1
MAREANO	NA	5	Diastylidae	Diastyloides serratus	6.3110	59.2900	643	1
MAREANO	NA	5	Diastylidae	Diastyloides serratus	4.9850	58.9910	234	1
MAREANO	NA	5	Diastylidae	Diastyloides serratus	5.5900	59.7600	368	1
MAREANO	NA	5	Diastylidae	Diastyloides serratus	5.4280	61.0480	1261	1
MAREANO	NA	5	Diastylidae	Diastyloides serratus	6.7360	61.3550	181	1
MAREANO	NA	5	Diastylidae	Diastyloides serratus	6.5980	61.1700	1092	1
MAREANO	NA	5	Diastylidae	Diastyloides serratus	6.5700	61.2350	294	1
MAREANO	NA	5	Diastylidae	Diastyloides serratus	7.0460	61.0130	503	1
MAREANO	NA	5	Diastylidae	Diastyloides serratus	6.0550	62.4030	449	1
MAREANO	NA	5	Diastylidae	Diastyloides serratus	4.9200	60.8480	461	1
MAREANO	NA	5	Diastylidae	Diastyloides serratus	5.4080	61.0510	1250	1
MAREANO	NA	5	Diastylidae	Diastyloides serratus	5.9000	61.1450	1280	1
MAREANO	NA	5	Diastylidae	Diastyloides serratus	5.6000	61.1100	1257	1
MAREANO	NA	5	Diastylidae	Diastyloides serratus	7.5950	62.6980	360	1
MAREANO	NA	5	Diastylidae	Diastyloides serratus	7.6650	62.6230	422	1
MAREANO	NA	5	Diastylidae	Diastyloides serratus	5.5930	61.9110	581	1
MAREANO	NA	5	Diastylidae	Diastyloides serratus	4.9830	60.6250	410	1
MAREANO	NA	5	Diastylidae	Diastyloides serratus	5.4410	61.3800	147	1
MAREANO	NA	5	Diastylidae	Diastyloides serratus	5.2350	61.3550	348	1
MAREANO	NA	5	Diastylidae	Diastyloides serratus	5.1780	61.3210	419	1
MAREANO	NA	5	Diastylidae	Diastyloides serratus	5.0460	61.3100	339	1
MAREANO	NA	5	Diastylidae	Diastyloides serratus	5.5900	61.1030	1257	1
MAREANO	NA	5	Diastylidae	Diastyloides serratus	3.5100	60.7460	320	1
MAREANO	NA	5	Diastylidae	Diastyloides serratus	3.5030	60.0020	270	1
MAREANO	NA	5	Diastylidae	Diastyloides serratus	10.7732	67.6730	175-175	1
MAREANO	NA	5	Diastylidae	Diastyloides serratus	20.8183	70.7703	246-247	1
MAREANO	NA	5	Diastylidae	Diastyloides serratus	21.0303	70.7015	258-260	1
MAREANO	NA	5	Diastylidae	Diastyloides serratus	11.8142	67.8468	183-173	1
MAREANO	NA	5	Diastylidae	Diastyloides serratus	10.1875	67.3982	224-223	1
MAREANO	NA	5	Diastylidae	Diastyloides serratus	11.1134	67.7971	265-266	1
MAREANO	NA	5	Leuconidae	Eudorella arctica	8.6419	67.3496	849-842	1
MAREANO	NA	5	Leuconidae	Eudorella arctica	9.8800	67.8962	777-775	1
MAREANO	NA	5	Leuconidae	Eudorella arctica	8.1330	67.2843	1117-1116	1
MAREANO	NA	5	Leuconidae	Eudorella cf. truncatula (Bate, 1856)	5.2750	60.4230	310	1
MAREANO	NA	5	Leuconidae	Eudorella cf. truncatula (Bate, 1856)	1.5400	62.4110	575	1
MAREANO	NA	5	Leuconidae	Eudorella emarginata	5.3160	61.9010	490	1
MAREANO	NA	5	Leuconidae	Eudorella emarginata	5.0380	61.7880	380	1

Supplemental Table xx Data source and station information on specimens incorporated in the distribution maps.

Data source	Station	Ecoregion	Taxon 1	Taxon 2	decLong	decLat	minDepth	Specimen count
MAREANO	NA	5	Leuconidae	Eudorella emarginata	13.0110	67.4860	264	1
MAREANO	NA	5	Leuconidae	Eudorella emarginata	5.4450	61.9230	584	1
MAREANO	NA	5	Leuconidae	Eudorella emarginata	5.4450	61.9230	584	1
MAREANO	NA	5	Leuconidae	Eudorella emarginata	5.8080	61.1850	230	1
MAREANO	NA	5	Leuconidae	Eudorella emarginata	6.4180	61.0500	161	1
MAREANO	NA	5	Leuconidae	Eudorella emarginata	6.6810	61.3100	217	1
MAREANO	NA	5	Leuconidae	Eudorella emarginata	7.6600	61.2300	155	1
MAREANO	NA	5	Leuconidae	Eudorella emarginata	7.3660	61.3450	373	1
MAREANO	NA	5	Leuconidae	Eudorella emarginata	5.4210	61.9230	580	1
MAREANO	NA	5	Leuconidae	Eudorella emarginata	5.4680	61.9250	580	1
MAREANO	NA	5	Leuconidae	Eudorella emarginata	6.5610	60.1660	296	1
MAREANO	NA	5	Leuconidae	Eudorella emarginata	6.5930	60.2450	386	1
MAREANO	NA	5	Leuconidae	Eudorella emarginata	6.4430	60.4160	858	1
MAREANO	NA	5	Leuconidae	Eudorella emarginata	6.2210	60.3780	63	1
MAREANO	NA	5	Leuconidae	Eudorella emarginata	6.2210	60.3780	63	1
MAREANO	NA	5	Leuconidae	Eudorella emarginata	6.2060	60.2980	835	1
MAREANO	NA	5	Leuconidae	Eudorella emarginata	6.2330	60.1180	271	1
MAREANO	NA	5	Leuconidae	Eudorella emarginata	6.2730	60.1410	204	1
MAREANO	NA	5	Leuconidae	Eudorella emarginata	5.9780	59.9700	166	1
MAREANO	NA	5	Leuconidae	Eudorella emarginata	3.4900	60.2550	291	1
MAREANO	NA	5	Leuconidae	Eudorella emarginata	5.4760	60.4560	220	1
MAREANO	NA	5	Leuconidae	Eudorella emarginata	5.2360	60.4850	315	1
MAREANO	NA	5	Leuconidae	Eudorella emarginata	6.4410	59.3160	110	1
MAREANO	NA	5	Leuconidae	Eudorella emarginata	6.4410	59.3180	110	1
MAREANO	NA	5	Leuconidae	Eudorella emarginata	4.9850	58.9910	234	1
MAREANO	NA	5	Leuconidae	Eudorella emarginata	5.9700	59.8080	286	1
MAREANO	NA	5	Leuconidae	Eudorella emarginata	6.7360	61.3550	181	1
MAREANO	NA	5	Leuconidae	Eudorella emarginata	6.0480	61.1860	254	1
MAREANO	NA	5	Leuconidae	Eudorella emarginata	5.5060	62.0610	150	1
MAREANO	NA	5	Leuconidae	Eudorella emarginata	5.5930	61.9110	581	1
MAREANO	NA	5	Leuconidae	Eudorella emarginata	3.5030	60.0020	270	1
MAREANO	NA	5	Leuconidae	Eudorella emarginata	10.1875	67.3982	224-223	1
MAREANO	NA	5	Leuconidae	Eudorella hirsuta	5.8230	62.4700	264	1
MAREANO	NA	5	Leuconidae	Eudorella hirsuta	5.7830	63.0660	217	1
MAREANO	NA	5	Leuconidae	Eudorella hirsuta	6.4630	62.4680	201	1
MAREANO	NA	5	Leuconidae	Eudorella hirsuta	5.0380	61.7880	380	1
MAREANO	NA	5	Leuconidae	Eudorella hirsuta	8.8310	65.7160	438	1
MAREANO	NA	5	Leuconidae	Eudorella hirsuta	7.8800	63.1400	326	1
MAREANO	NA	5	Leuconidae	Eudorella hirsuta	4.8730	60.8450	460	1
MAREANO	NA	5	Leuconidae	Eudorella hirsuta	5.1160	61.4660	419	1
MAREANO	NA	5	Leuconidae	Eudorella hirsuta	5.4450	61.9230	584	1
MAREANO	NA	5	Leuconidae	Eudorella hirsuta	3.0830	61.4930	400	1
MAREANO	NA	5	Leuconidae	Eudorella hirsuta	5.7680	61.1430	1260	1
MAREANO	NA	5	Leuconidae	Eudorella hirsuta	6.4180	61.0500	161	1
MAREANO	NA	5	Leuconidae	Eudorella hirsuta	7.5750	61.2010	277	1
MAREANO	NA	5	Leuconidae	Eudorella hirsuta	7.1480	61.1280	935	1
MAREANO	NA	5	Leuconidae	Eudorella hirsuta	2.7650	63.1780	1030	1
MAREANO	NA	5	Leuconidae	Eudorella hirsuta	3.2180	62.9850	804	1
MAREANO	NA	5	Leuconidae	Eudorella hirsuta	5.4210	61.9230	580	1
MAREANO	NA	5	Leuconidae	Eudorella hirsuta	5.1200	61.4680	420	1
MAREANO	NA	5	Leuconidae	Eudorella hirsuta	5.3750	61.0560	1250	1
MAREANO	NA	5	Leuconidae	Eudorella hirsuta	2.9810	62.1650	400	1
MAREANO	NA	5	Leuconidae	Eudorella hirsuta	0.8080	63.0480	1286	1
MAREANO	NA	5	Leuconidae	Eudorella hirsuta	3.1210	63.2130	1003	1
MAREANO	NA	5	Leuconidae	Eudorella hirsuta	5.4150	62.0480	1252	1
MAREANO	NA	5	Leuconidae	Eudorella hirsuta	5.9310	60.1300	499	1
MAREANO	NA	5	Leuconidae	Eudorella hirsuta	6.0930	60.2350	649	1
MAREANO	NA	5	Leuconidae	Eudorella hirsuta	6.2060	60.2980	835	1
MAREANO	NA	5	Leuconidae	Eudorella hirsuta	6.2330	60.1180	271	1
MAREANO	NA	5	Leuconidae	Eudorella hirsuta	5.9780	59.9700	166	1
MAREANO	NA	5	Leuconidae	Eudorella hirsuta	5.4760	60.4560	220	1
MAREANO	NA	5	Leuconidae	Eudorella hirsuta	5.2360	60.4850	315	1
MAREANO	NA	5	Leuconidae	Eudorella hirsuta	5.2750	60.4230	310	1
MAREANO	NA	5	Leuconidae	Eudorella hirsuta	2.0020	61.5000	311	1
MAREANO	NA	5	Leuconidae	Eudorella hirsuta	2.5100	61.7350	388	1
MAREANO	NA	5	Leuconidae	Eudorella hirsuta	3.5150	61.2510	374	1
MAREANO	NA	5	Leuconidae	Eudorella hirsuta	3.9680	60.9950	343	1
MAREANO	NA	5	Leuconidae	Eudorella hirsuta	5.5900	59.7600	368	1
MAREANO	NA	5	Leuconidae	Eudorella hirsuta	1.2330	62.5930	781	1
MAREANO	NA	5	Leuconidae	Eudorella hirsuta	7.0460	61.0130	503	1
MAREANO	NA	5	Leuconidae	Eudorella hirsuta	5.4080	61.0510	1250	1
MAREANO	NA	5	Leuconidae	Eudorella hirsuta	1.1860	62.7060	897	1
MAREANO	NA	5	Leuconidae	Eudorella hirsuta	5.6000	61.1100	1257	1
MAREANO	NA	5	Leuconidae	Eudorella hirsuta	7.6650	62.6230	422	1
MAREANO	NA	5	Leuconidae	Eudorella hirsuta	5.5930	61.9110	581	1
MAREANO	NA	5	Leuconidae	Eudorella hirsuta	5.0460	61.3100	339	1
MAREANO	NA	5	Leuconidae	Eudorella truncatula	4.8730	60.8450	470	1
MAREANO	NA	5	Leuconidae	Eudorella truncatula	6.4660	62.9780	147	1
MAREANO	NA	5	Leuconidae	Eudorella truncatula	4.4130	63.2850	1260	1

Supplemental Table xx Data source and station information on specimens incorporated in the distribution maps.

Data source	Station	Ecoregion	Taxon 1	Taxon 2	decLong	decLat	minDepth	Specimen count
MAREANO	NA	5	Leuconidae	Eudorella truncatula	5.2380	65.7160	794	1
MAREANO	NA	5	Leuconidae	Eudorella truncatula	4.8150	65.6660	996	1
MAREANO	NA	5	Leuconidae	Eudorella truncatula	4.3810	65.6960	1211	1
MAREANO	NA	5	Leuconidae	Eudorella truncatula	3.1550	65.6460	1500	1
MAREANO	NA	5	Leuconidae	Eudorella truncatula	5.0530	60.8300	544	1
MAREANO	NA	5	Leuconidae	Eudorella truncatula	5.7060	63.1200	192	1
MAREANO	NA	5	Leuconidae	Eudorella truncatula	4.0900	63.4230	1288	1
MAREANO	NA	5	Leuconidae	Eudorella truncatula	1.0430	62.8000	1009	1
MAREANO	NA	5	Leuconidae	Eudorella truncatula	1.0450	62.8060	1003	1
MAREANO	NA	5	Leuconidae	Eudorella truncatula	0.9810	62.5530	800	1
MAREANO	NA	5	Leuconidae	Eudorella truncatula	1.7210	62.4910	604	1
MAREANO	NA	5	Leuconidae	Eudorella truncatula	4.9250	60.8580	460	1
MAREANO	NA	5	Leuconidae	Eudorella truncatula	3.2180	62.9850	804	1
MAREANO	NA	5	Leuconidae	Eudorella truncatula	6.2210	60.3780	63	1
MAREANO	NA	5	Leuconidae	Eudorella truncatula	5.4760	60.4560	220	1
MAREANO	NA	5	Leuconidae	Eudorella truncatula	5.2360	60.4850	315	1
MAREANO	NA	5	Leuconidae	Eudorella truncatula	6.4410	59.3160	110	1
MAREANO	NA	5	Leuconidae	Eudorella truncatula	6.4410	59.3180	110	1
MAREANO	NA	5	Leuconidae	Eudorella truncatula	-0.0030	62.1980	708	1
MAREANO	NA	5	Leuconidae	Eudorella truncatula	4.9200	60.8480	461	1
MAREANO	NA	5	Leuconidae	Eudorella truncatula	1.8950	63.1330	1087	1
MAREANO	NA	5	Leuconidae	Eudorella truncatula	1.8200	62.5530	625	1
MAREANO	NA	5	Leuconidae	Eudorella truncatula	1.4430	62.5250	701	1
MAREANO	NA	5	Leuconidae	Eudorella truncatula	0.9280	62.9110	1112	1
MAREANO	NA	5	Leuconidae	Eudorella truncatula	1.7630	62.6660	746	1
MAREANO	NA	5	Leuconidae	Eudorella truncatula	7.5950	62.6980	360	1
MAREANO	NA	5	Leuconidae	Eudorella truncatula	4.9830	60.6250	410	1
MAREANO	NA	5	Leuconidae	Eudorella truncatula	5.4410	61.3800	147	1
MAREANO	NA	5	Leuconidae	Eudorella truncatula	5.1780	61.3210	419	1
MAREANO	NA	5	Leuconidae	Eudorella truncatula	5.4800	61.0230	121	1
MAREANO	NA	5	Leuconidae	Eudorella truncatula	4.0070	60.7410	315	1
MAREANO	NA	5	Leuconidae	Eudorella truncatula	4.0130	60.2360	297	1
MAREANO	NA	5	Leuconidae	Eudorella truncatula	11.8142	67.8468	183-173	1
MAREANO	NA	5	Leuconidae	Eudorella truncatula	7.7382	63.0008	100	1
MAREANO	NA	5	Leuconidae	Eudorellopsis deformis	3.9680	60.9950	343	1
MAREANO	NA	5	Leuconidae	Eudorellopsis deformis	2.7960	60.6010	108	1
MAREANO	NA	5	Leuconidae	Eudorellopsis deformis	2.8110	60.5780	106	1
MAREANO	NA	5	Leuconidae	Eudorellopsis deformis	2.7430	60.5000	104	1
MAREANO	NA	5	Lampropidae	Hemilamprops assimilis	5.7830	63.0660	217	1
MAREANO	NA	5	Lampropidae	Hemilamprops assimilis	6.3380	63.0050	160	1
MAREANO	NA	5	Lampropidae	Hemilamprops assimilis	6.4660	62.9780	147	1
MAREANO	NA	5	Lampropidae	Hemilamprops assimilis	5.7800	63.0950	210	1
MAREANO	NA	5	Lampropidae	Hemilamprops assimilis	6.4630	62.4680	201	1
MAREANO	NA	5	Lampropidae	Hemilamprops assimilis	5.8360	62.4680	258	1
MAREANO	NA	5	Lampropidae	Hemilamprops assimilis	5.7060	63.1200	192	1
MAREANO	NA	5	Lampropidae	Hemilamprops assimilis	2.0360	62.4710	502	1
MAREANO	NA	5	Lampropidae	Hemilamprops assimilis	1.7410	62.4860	602	1
MAREANO	NA	5	Lampropidae	Hemilamprops assimilis	4.4500	62.4730	216	1
MAREANO	NA	5	Lampropidae	Hemilamprops assimilis	4.4500	62.4730	216	1
MAREANO	NA	5	Lampropidae	Hemilamprops assimilis	1.9500	62.4300	473	1
MAREANO	NA	5	Lampropidae	Hemilamprops assimilis	2.1310	62.4660	458	1
MAREANO	NA	5	Lampropidae	Hemilamprops assimilis	2.1310	62.4650	456	1
MAREANO	NA	5	Lampropidae	Hemilamprops assimilis	11.6260	67.3902	128-139	1
MAREANO	NA	5	Lampropidae	Hemilamprops assimilis	21.1220	70.9638	149-136	1
MAREANO	NA	5	Lampropidae	Hemilamprops cristatus	11.8142	67.8468	183-173	1
MAREANO	NA	5	Lampropidae	Hemilamprops cristatus	4.0193	61.3636	358	1
MAREANO	NA	5	Lampropidae	Hemilamprops cristatus	4.0000	60.1330	295	1
MAREANO	NA	5	Lampropidae	Hemilamprops cristatus	6.3380	63.0050	160	1
MAREANO	NA	5	Lampropidae	Hemilamprops cristatus	6.0810	63.4150	272	1
MAREANO	NA	5	Lampropidae	Hemilamprops cristatus	6.4660	62.9780	147	1
MAREANO	NA	5	Lampropidae	Hemilamprops cristatus	6.4630	62.4680	201	1
MAREANO	NA	5	Lampropidae	Hemilamprops cristatus	5.3160	61.9010	490	1
MAREANO	NA	5	Lampropidae	Hemilamprops cristatus	13.0110	67.4860	264	1
MAREANO	NA	5	Lampropidae	Hemilamprops cristatus	9.1280	67.1760	462	1
MAREANO	NA	5	Lampropidae	Hemilamprops cristatus	7.4260	66.0210	454	1
MAREANO	NA	5	Lampropidae	Hemilamprops cristatus	8.8310	65.7160	438	1
MAREANO	NA	5	Lampropidae	Hemilamprops cristatus	7.0430	65.6160	398	1
MAREANO	NA	5	Lampropidae	Hemilamprops cristatus	5.6330	65.6860	602	1
MAREANO	NA	5	Lampropidae	Hemilamprops cristatus	7.0150	64.2580	343	1
MAREANO	NA	5	Lampropidae	Hemilamprops cristatus	7.0300	64.2400	341	1
MAREANO	NA	5	Lampropidae	Hemilamprops cristatus	8.6960	64.2550	517	1
MAREANO	NA	5	Lampropidae	Hemilamprops cristatus	7.8800	63.1400	326	1
MAREANO	NA	5	Lampropidae	Hemilamprops cristatus	2.0360	62.4710	502	1
MAREANO	NA	5	Lampropidae	Hemilamprops cristatus	3.8300	61.3230	368	1
MAREANO	NA	5	Lampropidae	Hemilamprops cristatus	2.1360	62.4950	497	1
MAREANO	NA	5	Lampropidae	Hemilamprops cristatus	4.5660	62.1230	225	1
MAREANO	NA	5	Lampropidae	Hemilamprops cristatus	4.4500	62.4730	216	1
MAREANO	NA	5	Lampropidae	Hemilamprops cristatus	4.4500	62.4730	216	1
MAREANO	NA	5	Lampropidae	Hemilamprops cristatus	4.4910	59.2550	255	1

Supplemental Table xx Data source and station information on specimens incorporated in the distribution maps.

Data source	Station	Ecoregion	Taxon 1	Taxon 2	decLong	decLat	minDepth	Specimen count
MAREANO	NA	5	Lampropidae	Hemilamprops cristatus	3.4900	60.2550	291	1
MAREANO	NA	5	Lampropidae	Hemilamprops cristatus	4.4710	60.2530	306	1
MAREANO	NA	5	Lampropidae	Hemilamprops cristatus	2.0020	61.5000	311	1
MAREANO	NA	5	Lampropidae	Hemilamprops cristatus	2.5100	61.7350	388	1
MAREANO	NA	5	Lampropidae	Hemilamprops cristatus	3.5150	61.2510	374	1
MAREANO	NA	5	Lampropidae	Hemilamprops cristatus	3.9680	60.9950	343	1
MAREANO	NA	5	Lampropidae	Hemilamprops cristatus	4.9850	58.9910	234	1
MAREANO	NA	5	Lampropidae	Hemilamprops cristatus	-0.0030	62.1980	708	1
MAREANO	NA	5	Lampropidae	Hemilamprops cristatus	1.8200	62.5530	625	1
MAREANO	NA	5	Lampropidae	Hemilamprops cristatus	1.8360	62.4200	501	1
MAREANO	NA	5	Lampropidae	Hemilamprops cristatus	2.0210	62.0020	374	1
MAREANO	NA	5	Lampropidae	Hemilamprops cristatus	5.9000	61.1450	1280	1
MAREANO	NA	5	Lampropidae	Hemilamprops cristatus	1.9500	62.4300	473	1
MAREANO	NA	5	Lampropidae	Hemilamprops cristatus	1.5400	62.4110	575	1
MAREANO	NA	5	Lampropidae	Hemilamprops cristatus	2.4510	61.0730	174	1
MAREANO	NA	5	Lampropidae	Hemilamprops cristatus	2.5880	61.1280	257	1
MAREANO	NA	5	Lampropidae	Hemilamprops cristatus	2.5880	61.1280	260	1
MAREANO	NA	5	Lampropidae	Hemilamprops cristatus	2.5060	61.1010	209	1
MAREANO	NA	5	Lampropidae	Hemilamprops cristatus	2.5130	61.1000	211	1
MAREANO	NA	5	Lampropidae	Hemilamprops cristatus	2.5830	61.1280	257	1
MAREANO	NA	5	Lampropidae	Hemilamprops cristatus	2.4580	61.0660	174	1
MAREANO	NA	5	Lampropidae	Hemilamprops cristatus	2.5760	61.1260	255	1
MAREANO	NA	5	Lampropidae	Hemilamprops cristatus	2.1310	62.4660	458	1
MAREANO	NA	5	Lampropidae	Hemilamprops cristatus	4.0070	60.7410	315	1
MAREANO	NA	5	Lampropidae	Hemilamprops cristatus	3.5100	60.7460	320	1
MAREANO	NA	5	Lampropidae	Hemilamprops cristatus	3.5030	60.0020	270	1
MAREANO	NA	5	Lampropidae	Hemilamprops cristatus	4.0130	60.2360	297	1
MAREANO	NA	5	Lampropidae	Hemilamprops cristatus	3.9584	61.3432	360	1
MAREANO	NA	5	Lampropidae	Hemilamprops cristatus	3.9634	61.3407	360	1
MAREANO	NA	5	Lampropidae	Hemilamprops cristatus	3.9803	61.3861	360	1
MAREANO	NA	5	Lampropidae	Hemilamprops cristatus	10.7732	67.6730	175-175	1
MAREANO	NA	5	Lampropidae	Hemilamprops cristatus	21.5623	70.7518	308-312	1
MAREANO	NA	5	Lampropidae	Hemilamprops cristatus	21.0303	70.7015	258-260	1
MAREANO	NA	5	Lampropidae	Hemilamprops roseus	5.7830	63.0660	217	1
MAREANO	NA	5	Lampropidae	Hemilamprops roseus	6.3380	63.0050	160	1
MAREANO	NA	5	Lampropidae	Hemilamprops roseus	6.0810	63.4150	272	1
MAREANO	NA	5	Lampropidae	Hemilamprops roseus	6.4660	62.9780	147	1
MAREANO	NA	5	Lampropidae	Hemilamprops roseus	5.7800	63.0950	210	1
MAREANO	NA	5	Lampropidae	Hemilamprops roseus	4.4500	62.4730	216	1
MAREANO	NA	5	Lampropidae	Hemilamprops roseus	3.4900	60.2550	291	1
MAREANO	NA	5	Lampropidae	Hemilamprops roseus	2.7960	60.6010	108	1
MAREANO	NA	5	Lampropidae	Hemilamprops roseus	2.8110	60.5780	106	1
MAREANO	NA	5	Lampropidae	Hemilamprops roseus	2.7430	60.5000	104	1
MAREANO	NA	5	Lampropidae	Hemilamprops roseus	2.4530	61.0730	174	1
MAREANO	NA	5	Lampropidae	Hemilamprops roseus	2.5880	61.1280	257	1
MAREANO	NA	5	Lampropidae	Hemilamprops roseus	2.5880	61.1280	260	1
MAREANO	NA	5	Lampropidae	Hemilamprops roseus	2.5160	61.0980	211	1
MAREANO	NA	5	Lampropidae	Hemilamprops roseus	2.5060	61.1010	209	1
MAREANO	NA	5	Lampropidae	Hemilamprops roseus	2.5130	61.1000	211	1
MAREANO	NA	5	Lampropidae	Hemilamprops roseus	2.5830	61.1280	257	1
MAREANO	NA	5	Lampropidae	Hemilamprops roseus	2.3150	61.0030	135	1
MAREANO	NA	5	Lampropidae	Hemilamprops roseus	2.4530	61.0660	174	1
MAREANO	NA	5	Lampropidae	Hemilamprops roseus	2.4580	61.0660	174	1
MAREANO	NA	5	Lampropidae	Hemilamprops roseus	2.5760	61.1260	255	1
MAREANO	NA	5	Lampropidae	Hemilamprops roseus	5.4800	61.0230	121	1
MAREANO	NA	5	Lampropidae	Hemilamprops roseus	4.4957	62.6143	203-203	1
MAREANO	NA	5	Lampropidae	Hemilamprops roseus	21.0303	70.7015	258-260	1
MAREANO	NA	5	Lampropidae	Hemilamprops roseus	10.7732	67.6730	175-175	1
MAREANO	NA	5	Lampropidae	Hemilamprops roseus	11.6260	67.3902	128-139	1
MAREANO	NA	5	Lampropidae	Hemilamprops roseus	7.8637	63.0348	100	1
MAREANO	NA	5	Lampropidae	Hemilamprops uniplicatus	6.3380	63.0050	160	1
MAREANO	NA	5	Lampropidae	Hemilamprops uniplicatus	5.7800	63.0950	210	1
MAREANO	NA	5	Lampropidae	Hemilamprops uniplicatus	5.8360	62.4680	258	1
MAREANO	NA	5	Lampropidae	Hemilamprops uniplicatus	7.7310	67.7830	2025	1
MAREANO	NA	5	Lampropidae	Hemilamprops uniplicatus	5.2380	65.7160	794	1
MAREANO	NA	5	Lampropidae	Hemilamprops uniplicatus	5.1160	61.4660	419	1
MAREANO	NA	5	Lampropidae	Hemilamprops uniplicatus	5.7060	63.1200	192	1
MAREANO	NA	5	Lampropidae	Hemilamprops uniplicatus	4.3530	60.8630	471	1
MAREANO	NA	5	Lampropidae	Hemilamprops uniplicatus	3.8300	61.3230	368	1
MAREANO	NA	5	Lampropidae	Hemilamprops uniplicatus	1.7210	62.4910	604	1
MAREANO	NA	5	Lampropidae	Hemilamprops uniplicatus	0.9810	62.5600	804	1
MAREANO	NA	5	Lampropidae	Hemilamprops uniplicatus	4.4500	62.4730	216	1
MAREANO	NA	5	Lampropidae	Hemilamprops uniplicatus	4.4500	62.4730	216	1
MAREANO	NA	5	Lampropidae	Hemilamprops uniplicatus	1.8200	62.5530	625	1
MAREANO	NA	5	Lampropidae	Hemilamprops uniplicatus	1.9500	62.4300	473	1
MAREANO	NA	5	Lampropidae	Hemilamprops uniplicatus	1.5400	62.4110	575	1
MAREANO	NA	5	Lampropidae	Hemilamprops uniplicatus	2.4530	61.0730	174	1
MAREANO	NA	5	Lampropidae	Hemilamprops uniplicatus	2.5880	61.1280	260	1
MAREANO	NA	5	Lampropidae	Hemilamprops uniplicatus	2.5160	61.0980	211	1

Supplemental Table xx Data source and station information on specimens incorporated in the distribution maps.

Data source	Station	Ecoregion	Taxon 1	Taxon 2	decLong	decLat	minDepth	Specimen count
MAREANO	NA	5	Lampropidae	Hemilamprops uniplicatus	2.5060	61.1010	209	1
MAREANO	NA	5	Lampropidae	Hemilamprops uniplicatus	2.7210	61.1930	315	1
MAREANO	NA	5	Lampropidae	Hemilamprops uniplicatus	2.5130	61.1000	211	1
MAREANO	NA	5	Lampropidae	Hemilamprops uniplicatus	2.3060	61.0030	137	1
MAREANO	NA	5	Lampropidae	Hemilamprops uniplicatus	2.3150	61.0030	135	1
MAREANO	NA	5	Lampropidae	Hemilamprops uniplicatus	2.3130	61.0020	135	1
MAREANO	NA	5	Lampropidae	Hemilamprops uniplicatus	2.4580	61.0660	174	1
MAREANO	NA	5	Lampropidae	Hemilamprops uniplicatus	2.5760	61.1260	255	1
MAREANO	NA	5	Lampropidae	Hemilamprops uniplicatus	11.6260	67.3902	128-139	1
MAREANO	NA	5	Lampropidae	Hemilamprops uniplicatus	9.8800	67.8962	777-775	1
MAREANO	NA	5	Lampropidae	Hemilamprops uniplicatus	8.9230	67.0960	401-401	1
MAREANO	NA	5	Lampropidae	Hemilamprops uniplicatus	10.3562	68.1885	799-890	1
MAREANO	NA	5	Lampropidae	Hemilamprops uniplicatus	4.4957	62.6143	203-203	1
MAREANO	NA	5	Diastylidae	Leptostylis ampullacea	2.7650	63.1780	1030	1
MAREANO	NA	5	Diastylidae	Leptostylis ampullacea	0.8080	63.0480	1286	1
MAREANO	NA	5	Diastylidae	Leptostylis ampullacea	0.6430	63.1660	1489	1
MAREANO	NA	5	Diastylidae	Leptostylis ampullacea	21.1220	70.9638	149-136	1
MAREANO	NA	5	Diastylidae	Leptostylis longimana cf.	7.0430	65.6160	398	1
MAREANO	NA	5	Diastylidae	Leptostylis macrura cf.	3.4900	60.2550	291	1
MAREANO	NA	5	Diastylidae	Leptostylis macrura cf.	5.2750	60.4230	310	1
MAREANO	NA	5	Diastylidae	Leptostylis longimana (G.O. Sars, 1865)	20.8183	70.7703	246-247	1
MAREANO	NA	5	Diastylidae	Leptostylis longimana (G.O. Sars, 1865)	21.5623	70.7518	308-312	1
MAREANO	NA	5	Diastylidae	Leptostylis longimana (G.O. Sars, 1865)	4.4130	63.2850	1260	1
MAREANO	NA	5	Diastylidae	Leptostylis longimana (G.O. Sars, 1865)	13.0110	67.4860	264	1
MAREANO	NA	5	Diastylidae	Leptostylis longimana (G.O. Sars, 1865)	4.2700	66.9830	1380	1
MAREANO	NA	5	Diastylidae	Leptostylis longimana (G.O. Sars, 1865)	7.4260	66.0210	454	1
MAREANO	NA	5	Diastylidae	Leptostylis longimana (G.O. Sars, 1865)	8.8310	65.7160	438	1
MAREANO	NA	5	Diastylidae	Leptostylis longimana (G.O. Sars, 1865)	4.8150	65.6660	996	1
MAREANO	NA	5	Diastylidae	Leptostylis longimana (G.O. Sars, 1865)	4.3810	65.6960	1211	1
MAREANO	NA	5	Diastylidae	Leptostylis longimana (G.O. Sars, 1865)	3.1550	65.6460	1500	1
MAREANO	NA	5	Diastylidae	Leptostylis longimana (G.O. Sars, 1865)	8.6960	64.2550	517	1
MAREANO	NA	5	Diastylidae	Leptostylis longimana (G.O. Sars, 1865)	7.8800	63.1400	326	1
MAREANO	NA	5	Diastylidae	Leptostylis longimana (G.O. Sars, 1865)	5.1160	61.4660	419	1
MAREANO	NA	5	Diastylidae	Leptostylis longimana (G.O. Sars, 1865)	5.4450	61.9230	584	1
MAREANO	NA	5	Diastylidae	Leptostylis longimana (G.O. Sars, 1865)	7.0460	61.0150	506	1
MAREANO	NA	5	Diastylidae	Leptostylis longimana (G.O. Sars, 1865)	6.5950	61.1530	1106	1
MAREANO	NA	5	Diastylidae	Leptostylis longimana (G.O. Sars, 1865)	6.9030	61.1230	860	1
MAREANO	NA	5	Diastylidae	Leptostylis longimana (G.O. Sars, 1865)	7.1480	61.1280	935	1
MAREANO	NA	5	Diastylidae	Leptostylis longimana (G.O. Sars, 1865)	2.7650	63.1780	1030	1
MAREANO	NA	5	Diastylidae	Leptostylis longimana (G.O. Sars, 1865)	3.2180	62.9850	804	1
MAREANO	NA	5	Diastylidae	Leptostylis longimana (G.O. Sars, 1865)	5.4210	61.9230	580	1
MAREANO	NA	5	Diastylidae	Leptostylis longimana (G.O. Sars, 1865)	5.1200	61.4680	420	1
MAREANO	NA	5	Diastylidae	Leptostylis longimana (G.O. Sars, 1865)	5.3750	61.0560	1250	1
MAREANO	NA	5	Diastylidae	Leptostylis longimana (G.O. Sars, 1865)	5.1210	61.4680	418	1
MAREANO	NA	5	Diastylidae	Leptostylis longimana (G.O. Sars, 1865)	5.4680	61.9250	580	1
MAREANO	NA	5	Diastylidae	Leptostylis longimana (G.O. Sars, 1865)	0.8080	63.0480	1286	1
MAREANO	NA	5	Diastylidae	Leptostylis longimana (G.O. Sars, 1865)	2.5150	66.6260	1626	1
MAREANO	NA	5	Diastylidae	Leptostylis longimana (G.O. Sars, 1865)	3.1210	63.2130	1003	1
MAREANO	NA	5	Diastylidae	Leptostylis longimana (G.O. Sars, 1865)	5.9310	60.1300	499	1
MAREANO	NA	5	Diastylidae	Leptostylis longimana (G.O. Sars, 1865)	6.0930	60.2350	649	1
MAREANO	NA	5	Diastylidae	Leptostylis longimana (G.O. Sars, 1865)	6.3100	60.3600	855	1
MAREANO	NA	5	Diastylidae	Leptostylis longimana (G.O. Sars, 1865)	6.2060	60.2980	835	1
MAREANO	NA	5	Diastylidae	Leptostylis longimana (G.O. Sars, 1865)	5.9080	60.0080	659	1
MAREANO	NA	5	Diastylidae	Leptostylis longimana (G.O. Sars, 1865)	3.4900	60.2550	291	1
MAREANO	NA	5	Diastylidae	Leptostylis longimana (G.O. Sars, 1865)	2.0020	61.5000	311	1
MAREANO	NA	5	Diastylidae	Leptostylis longimana (G.O. Sars, 1865)	2.5100	61.7350	388	1
MAREANO	NA	5	Diastylidae	Leptostylis longimana (G.O. Sars, 1865)	3.5150	61.2510	374	1
MAREANO	NA	5	Diastylidae	Leptostylis longimana (G.O. Sars, 1865)	3.9680	60.9950	343	1
MAREANO	NA	5	Diastylidae	Leptostylis longimana (G.O. Sars, 1865)	5.5960	59.1760	557	1
MAREANO	NA	5	Diastylidae	Leptostylis longimana (G.O. Sars, 1865)	6.2410	59.5000	404	1
MAREANO	NA	5	Diastylidae	Leptostylis longimana (G.O. Sars, 1865)	4.9850	58.9910	234	1
MAREANO	NA	5	Diastylidae	Leptostylis longimana (G.O. Sars, 1865)	6.7360	61.3550	181	1
MAREANO	NA	5	Diastylidae	Leptostylis longimana (G.O. Sars, 1865)	7.0460	61.0130	503	1
MAREANO	NA	5	Diastylidae	Leptostylis longimana (G.O. Sars, 1865)	5.4080	61.0510	1250	1
MAREANO	NA	5	Diastylidae	Leptostylis longimana (G.O. Sars, 1865)	1.8950	63.1330	1087	1
MAREANO	NA	5	Diastylidae	Leptostylis longimana (G.O. Sars, 1865)	2.0210	62.0020	374	1
MAREANO	NA	5	Diastylidae	Leptostylis longimana (G.O. Sars, 1865)	0.9280	62.9110	1112	1
MAREANO	NA	5	Diastylidae	Leptostylis longimana (G.O. Sars, 1865)	-0.1610	63.7110	2259	1
MAREANO	NA	5	Diastylidae	Leptostylis longimana (G.O. Sars, 1865)	3.5030	60.0020	270	1
MAREANO	NA	5	Diastylidae	Leptostylis longimana (G.O. Sars, 1865)	20.1058	70.6262	289-293	1
MAREANO	NA	5	Diastylidae	Leptostylis longimana (G.O. Sars, 1865)	8.1330	67.2843	1117-1116	1
MAREANO	NA	5	Diastylidae	Leptostylis longimana (G.O. Sars, 1865)	9.6854	67.8046	823-809	1
MAREANO	NA	5	Diastylidae	Leptostylis macrura	5.7830	63.0660	217	1
MAREANO	NA	5	Diastylidae	Leptostylis macrura	6.3380	63.0050	160	1
MAREANO	NA	5	Diastylidae	Leptostylis macrura	6.4660	62.9780	147	1
MAREANO	NA	5	Diastylidae	Leptostylis macrura	6.4630	62.4680	201	1
MAREANO	NA	5	Diastylidae	Leptostylis macrura	5.8360	62.4680	258	1
MAREANO	NA	5	Diastylidae	Leptostylis macrura	8.8310	65.7160	438	1
MAREANO	NA	5	Diastylidae	Leptostylis macrura	7.0150	64.2580	343	1

Supplemental Table xx Data source and station information on specimens incorporated in the distribution maps.

Data source	Station	Ecoregion	Taxon 1	Taxon 2	decLong	decLat	minDepth	Specimen count
MAREANO	NA	5	Diastylidae	Leptostylis macrura	7.0300	64.2400	341	1
MAREANO	NA	5	Diastylidae	Leptostylis macrura	7.8800	63.1400	326	1
MAREANO	NA	5	Diastylidae	Leptostylis macrura	5.1160	61.4660	419	1
MAREANO	NA	5	Diastylidae	Leptostylis macrura	5.7950	62.4730	258	1
MAREANO	NA	5	Diastylidae	Leptostylis macrura	4.4500	62.4730	216	1
MAREANO	NA	5	Diastylidae	Leptostylis macrura	4.4500	62.4730	216	1
MAREANO	NA	5	Diastylidae	Leptostylis macrura	4.4710	60.2530	306	1
MAREANO	NA	5	Diastylidae	Leptostylis macrura	10.1875	67.3982	224-223	1
MAREANO	NA	5	Diastylidae	Leptostylis macrura	20.8183	70.7703	246-247	1
MAREANO	NA	5	Diastylidae	Leptostylis macrura	11.8142	67.8468	183-173	1
MAREANO	NA	5	Diastylidae	Leptostylis macrura	10.7732	67.6730	175-175	1
MAREANO	NA	5	Diastylidae	Leptostylis macrura	21.0303	70.7015	258-260	1
MAREANO	NA	5	Leuconidae	Leucon (Macrauloleucon) spinulosus	10.3010	68.6544	2744-2754	1
MAREANO	NA	5	Leuconidae	Leucon (Leucon) acutirostris	5.9780	59.9700	166	1
MAREANO	NA	5	Leuconidae	Leucon (Leucon) acutirostris	4.9850	58.9910	234	1
MAREANO	NA	5	Leuconidae	Leucon (Leucon) nasica	4.8730	60.8450	470	1
MAREANO	NA	5	Leuconidae	Leucon (Leucon) nasica	2.0360	62.4710	502	1
MAREANO	NA	5	Leuconidae	Leucon (Leucon) nasica	2.7650	63.1780	1030	1
MAREANO	NA	5	Leuconidae	Leucon (Leucon) nasica	3.2180	62.9850	804	1
MAREANO	NA	5	Leuconidae	Leucon (Leucon) nasica	7.0000	60.5710	150	1
MAREANO	NA	5	Leuconidae	Leucon (Leucon) nasica	7.0030	60.5710	150	1
MAREANO	NA	5	Leuconidae	Leucon (Leucon) nasica	6.2210	60.3780	63	1
MAREANO	NA	5	Leuconidae	Leucon (Leucon) nasica	6.2060	60.2980	835	1
MAREANO	NA	5	Leuconidae	Leucon (Leucon) nasica	5.9780	59.9700	166	1
MAREANO	NA	5	Leuconidae	Leucon (Leucon) nasica	5.2360	60.4850	315	1
MAREANO	NA	5	Leuconidae	Leucon (Leucon) nasica	4.9850	58.9910	234	1
MAREANO	NA	5	Leuconidae	Leucon (Leucon) nasica	5.9650	61.7800	145	1
MAREANO	NA	5	Leuconidae	Leucon (Leucon) nasica	5.4410	61.3800	147	1
MAREANO	NA	5	Leuconidae	Leucon (Leucon) nasica	5.2350	61.3550	348	1
MAREANO	NA	5	Leuconidae	Leucon (Leucon) nasica	3.5030	60.0020	270	1
MAREANO	NA	5	Leuconidae	Leucon (Leucon) nasicoides	9.6854	67.8046	823-809	1
MAREANO	NA	5	Leuconidae	Leucon (Leucon) nathorsti	8.2233	67.0213	543-556	1
MAREANO	NA	5	Leuconidae	Leucon (Crymoleucon) noerrevangi	1.9500	62.4300	473	1
MAREANO	NA	5	Leuconidae	Leucon pallidus	10.3562	68.1885	799-890	1
MAREANO	NA	5	Leuconidae	Leucon pallidus	9.6854	67.8046	823-809	1
MAREANO	NA	5	Leuconidae	Leucon pallidus	10.3562	68.1885	799-890	1
MAREANO	NA	5	Leuconidae	Leucon pallidus	21.5623	70.7518	308-312	1
MAREANO	NA	5	Leuconidae	Leucon pallidus	8.1330	67.2843	1117-1116	1
MAREANO	NA	5	Leuconidae	Leucon pallidus	21.0303	70.7015	258-260	1
MAREANO	NA	5	Leuconidae	Leucon pallidus	4.0000	60.1330	295	1
MAREANO	NA	5	Leuconidae	Leucon pallidus	5.8230	62.4700	264	1
MAREANO	NA	5	Leuconidae	Leucon pallidus	4.4130	63.2850	1260	1
MAREANO	NA	5	Leuconidae	Leucon pallidus	5.3160	61.9010	490	1
MAREANO	NA	5	Leuconidae	Leucon pallidus	13.0110	67.4860	264	1
MAREANO	NA	5	Leuconidae	Leucon pallidus	4.2700	66.9830	1380	1
MAREANO	NA	5	Leuconidae	Leucon pallidus	7.4260	66.0210	454	1
MAREANO	NA	5	Leuconidae	Leucon pallidus	8.8310	65.7160	438	1
MAREANO	NA	5	Leuconidae	Leucon pallidus	7.0430	65.6160	398	1
MAREANO	NA	5	Leuconidae	Leucon pallidus	4.8150	65.6660	996	1
MAREANO	NA	5	Leuconidae	Leucon pallidus	4.3810	65.6960	1211	1
MAREANO	NA	5	Leuconidae	Leucon pallidus	7.0150	64.2580	343	1
MAREANO	NA	5	Leuconidae	Leucon pallidus	7.0300	64.2400	341	1
MAREANO	NA	5	Leuconidae	Leucon pallidus	7.8800	63.1400	326	1
MAREANO	NA	5	Leuconidae	Leucon pallidus	4.8730	60.8450	460	1
MAREANO	NA	5	Leuconidae	Leucon pallidus	4.0900	63.4230	1288	1
MAREANO	NA	5	Leuconidae	Leucon pallidus	0.3580	63.5980	2104	1
MAREANO	NA	5	Leuconidae	Leucon pallidus	0.3830	63.5930	2090	1
MAREANO	NA	5	Leuconidae	Leucon pallidus	2.0360	62.4710	502	1
MAREANO	NA	5	Leuconidae	Leucon pallidus	1.0430	62.8000	1009	1
MAREANO	NA	5	Leuconidae	Leucon pallidus	1.0450	62.8060	1003	1
MAREANO	NA	5	Leuconidae	Leucon pallidus	0.9810	62.5530	800	1
MAREANO	NA	5	Leuconidae	Leucon pallidus	1.7410	62.4860	602	1
MAREANO	NA	5	Leuconidae	Leucon pallidus	3.0830	61.4930	400	1
MAREANO	NA	5	Leuconidae	Leucon pallidus	3.8300	61.3230	368	1
MAREANO	NA	5	Leuconidae	Leucon pallidus	1.7210	62.4910	604	1
MAREANO	NA	5	Leuconidae	Leucon pallidus	0.9810	62.5600	804	1
MAREANO	NA	5	Leuconidae	Leucon pallidus	4.9250	60.8580	460	1
MAREANO	NA	5	Leuconidae	Leucon pallidus	2.7650	63.1780	1030	1
MAREANO	NA	5	Leuconidae	Leucon pallidus	3.2180	62.9850	804	1
MAREANO	NA	5	Leuconidae	Leucon pallidus	4.5660	62.1230	225	1
MAREANO	NA	5	Leuconidae	Leucon pallidus	2.9810	62.1650	400	1
MAREANO	NA	5	Leuconidae	Leucon pallidus	0.8080	63.0480	1286	1
MAREANO	NA	5	Leuconidae	Leucon pallidus	0.8080	63.0480	1286	1
MAREANO	NA	5	Leuconidae	Leucon pallidus	2.5150	66.6260	1626	1
MAREANO	NA	5	Leuconidae	Leucon pallidus	3.1210	63.2130	1003	1
MAREANO	NA	5	Leuconidae	Leucon pallidus	5.9780	59.9700	166	1
MAREANO	NA	5	Leuconidae	Leucon pallidus	4.4910	59.2550	255	1
MAREANO	NA	5	Leuconidae	Leucon pallidus	3.4900	60.2550	291	1
MAREANO	NA	5	Leuconidae	Leucon pallidus	4.4710	60.2530	306	1

Supplemental Table xx Data source and station information on specimens incorporated in the distribution maps.

Data source	Station	Ecoregion	Taxon 1	Taxon 2	decLong	decLat	minDepth	Specimen count
MAREANO	NA	5	Leuconidae	Leucon pallidus	2.5100	61.7350	388	1
MAREANO	NA	5	Leuconidae	Leucon pallidus	3.5150	61.2510	374	1
MAREANO	NA	5	Leuconidae	Leucon pallidus	3.9680	60.9950	343	1
MAREANO	NA	5	Leuconidae	Leucon pallidus	4.9850	58.9910	234	1
MAREANO	NA	5	Leuconidae	Leucon pallidus	5.5900	59.7600	368	1
MAREANO	NA	5	Leuconidae	Leucon pallidus	2.9900	61.2410	378	1
MAREANO	NA	5	Leuconidae	Leucon pallidus	1.2330	62.5930	781	1
MAREANO	NA	5	Leuconidae	Leucon pallidus	1.8950	63.1330	1087	1
MAREANO	NA	5	Leuconidae	Leucon pallidus	1.8200	62.5530	625	1
MAREANO	NA	5	Leuconidae	Leucon pallidus	2.0210	62.0020	374	1
MAREANO	NA	5	Leuconidae	Leucon pallidus	1.4430	62.5250	701	1
MAREANO	NA	5	Leuconidae	Leucon pallidus	1.1860	62.7060	897	1
MAREANO	NA	5	Leuconidae	Leucon pallidus	0.9280	62.9110	1112	1
MAREANO	NA	5	Leuconidae	Leucon pallidus	1.7630	62.6660	746	1
MAREANO	NA	5	Leuconidae	Leucon pallidus	4.0130	60.2360	297	1
MAREANO	NA	5	Leuconidae	Leucon serratus	9.4483	67.1319	473-477	1
MAREANO	NA	5	Leuconidae	Leucon serratus	20.8183	70.7703	246-247	1
MAREANO	NA	5	Leuconidae	Leucon spinulosus	4.2700	66.9830	1380	1
MAREANO	NA	5	Leuconidae	Leucon spinulosus	2.9650	64.2750	2055	1
MAREANO	NA	5	Leuconidae	Leucon spinulosus	9.2487	68.2468	2346-2338	1
MAREANO	NA	5	Leuconidae	Leucon tener	9.1280	67.1760	462	1
MAREANO	NA	5	Leuconidae	Leucon tener	4.4910	59.2550	255	1
MAREANO	NA	5	Leuconidae	Leucon tener	4.4710	60.2530	306	1
MAREANO	NA	5	Leuconidae	Leucon tener	3.5150	61.2510	374	1
MAREANO	NA	5	Lampropidae	Mesolamprops denticulatus	1.8523	61.4814	273	1
MAREANO	NA	5	Pseudocumatidae	Petalosarsia declivis	20.6858	70.6020	114-92	1
MAREANO	NA	5	Pseudocumatidae	Petalosarsia declivis	1.9500	62.4300	473	1
MAREANO	NA	5	Lampropidae	Platysympus tricarinatus	6.4660	62.9780	147	1
MAREANO	NA	5	Lampropidae	Platysympus tricarinatus	6.4630	62.4680	201	1
MAREANO	NA	5	Lampropidae	Platysympus tricarinatus	5.4160	61.0460	1250	1
MAREANO	NA	5	Lampropidae	Platysympus tricarinatus	9.1280	67.1760	462	1
MAREANO	NA	5	Lampropidae	Platysympus tricarinatus	7.4260	66.0210	454	1
MAREANO	NA	5	Lampropidae	Platysympus tricarinatus	3.8300	61.3230	368	1
MAREANO	NA	5	Lampropidae	Platysympus tricarinatus	4.9250	60.8580	460	1
MAREANO	NA	5	Lampropidae	Platysympus tricarinatus	2.9810	62.1650	400	1
MAREANO	NA	5	Lampropidae	Platysympus tricarinatus	4.4710	60.2530	306	1
MAREANO	NA	5	Lampropidae	Platysympus tricarinatus	2.0210	62.0020	374	1
MAREANO	NA	5	Lampropidae	Platysympus tricarinatus	1.9500	62.4300	473	1
MAREANO	NA	5	Lampropidae	Platysympus tricarinatus	10.1875	67.3982	224-223	1
MAREANO	NA	5	Lampropidae	Platysympus typicus	8.9230	67.0960	401-401	1
MAREANO	NA	5	Lampropidae	Platysympus typicus	20.8183	70.7703	246-247	1
MAREANO	NA	5	Lampropidae	Platysympus typicus	21.0303	70.7015	258-260	1
MAREANO	NA	5	Lampropidae	Platysympus typicus	4.0000	60.1330	295	1
MAREANO	NA	5	Lampropidae	Platysympus typicus	6.3380	63.0050	160	1
MAREANO	NA	5	Lampropidae	Platysympus typicus	6.0810	63.4150	272	1
MAREANO	NA	5	Lampropidae	Platysympus typicus	7.0430	65.6160	398	1
MAREANO	NA	5	Lampropidae	Platysympus typicus	7.0150	64.2580	343	1
MAREANO	NA	5	Lampropidae	Platysympus typicus	7.0300	64.2400	341	1
MAREANO	NA	5	Lampropidae	Platysympus typicus	7.8800	63.1400	326	1
MAREANO	NA	5	Lampropidae	Platysympus typicus	4.5660	62.1230	225	1
MAREANO	NA	5	Lampropidae	Platysympus typicus	4.4910	59.2550	255	1
MAREANO	NA	5	Lampropidae	Platysympus typicus	2.7000	61.1810	307	1
MAREANO	NA	5	Lampropidae	Platysympus typicus	3.5030	60.0020	270	1
MAREANO	NA	5	Lampropidae	Platysympus typicus	8.9230	67.0960	401-401	1
MAREANO	NA	5	Nannastacidae	Procampylaspis armata	2.0020	61.5000	311	1
MAREANO	NA	5	Nannastacidae	Procampylaspis armata	2.5100	61.7350	388	1
MAREANO	NA	5	Nannastacidae	Procampylaspis armata	2.7000	61.1810	307	1
MAREANO	NA	5	Nannastacidae	Procampylaspis armata	2.5760	61.1260	255	1
MAREANO	NA	5	Pseudocumatidae	Pseudocuma (Pseudocuma) simile	3.9680	60.9950	343	1
MAREANO	NA	5	Pseudocumatidae	Pseudocuma (Pseudocuma) simile	2.8110	60.5780	106	1
MAREANO	NA	5	Pseudocumatidae	Pseudocuma (Pseudocuma) simile	2.9980	60.5030	118	1
MAREANO	NA	5	Pseudocumatidae	Pseudocuma (Pseudocuma) simile	2.7430	60.5000	104	1
MAREANO	NA	7	Nannastacidae	Campylaspis rubicunda	27.0157	71.2755	278-277	1
MAREANO	NA	7	Nannastacidae	Campylaspis verrucosa	27.0157	71.2755	278-277	1
MAREANO	NA	7	Nannastacidae	Campylaspis verrucosa	25.2195	71.3377	297-297	1
MAREANO	NA	7	Diastylidae	Diastylis echinata	25.2195	71.3377	297-297	1
MAREANO	NA	7	Diastylidae	Diastylis edwardsii	14.4833	78.3050	57	1
MAREANO	NA	7	Diastylidae	Diastylis spinulosa	25.2195	71.3377	297-297	1
MAREANO	NA	7	Leuconidae	Eudorella hirsuta	27.0157	71.2755	278-277	1
MAREANO	NA	7	Lampropidae	Hemilamprops assimilis	25.2195	71.3377	297-297	1
MAREANO	NA	7	Lampropidae	Hemilamprops assimilis	27.0157	71.2755	278-277	1
MAREANO	NA	7	Lampropidae	Hemilamprops cristatus	25.2195	71.3377	297-297	1
MAREANO	NA	7	Lampropidae	Hemilamprops cristatus	27.0157	71.2755	278-277	1
MAREANO	NA	7	Lampropidae	Hemilamprops roseus	27.7562	71.4538	400-401	1
MAREANO	NA	7	Diastylidae	Leptostylis longimana (G.O. Sars, 1865)	27.7562	71.4538	400-401	1
MAREANO	NA	7	Diastylidae	Leptostylis villosa	27.7562	71.4538	400-401	1
MAREANO	NA	7	Diastylidae	Leptostylis villosa	18.9175	79.6116	337	1
MAREANO	NA	7	Leuconidae	Leucon (Leucon) nasica	27.0157	71.2755	278-277	1
MAREANO	NA	7	Leuconidae	Leucon (Leucon) nathorsti	27.7562	71.4538	400-401	1

Supplemental Table xx Data source and station information on specimens incorporated in the distribution maps.

Data source	Station	Ecoregion	Taxon 1	Taxon 2	decLong	decLat	minDepth	Specimen count
MAREANO	NA	7	Leuconidae	Leucon (Leucon) nathorsti	27.0157	71.2755	278-277	1
MAREANO	NA	7	Pseudocumatidae	Petalosarsia declivis	25.2195	71.3377	297-297	1
MAREANO	NA	7	Pseudocumatidae	Petalosarsia declivis	25.2195	71.3377	297-297	1
