# Supplementary material for: Adding pieces to the puzzle: insights into diversity and distribution patterns of Cumacea (Crustacea: Peracarida) from the deep North Atlantic to the Arctic Ocean
Source: PeerJ. 2021 Nov 11;9:e12379. doi: 10.7717/peerj.12379 (PMC8590803; doi:10.7717/peerj.12379)
Supplement: Supplemental Information 11 — Morphological identification of 947 investigated specimens, resulting in 77 putative species. Field ID was assigned to each specimen during DNA extraction. Out of 123 extracted specimens, 80 yielded sequence data of sufficient quality to be included in the molecular species delimitation (highlighted in bold). [file peerj-09-12379-s011.pdf]

Species ID	Project	Station	Sample ID	GenBank Accession	Family	Putative species	Specimen count	Sequence ID (Field ID)
**Bod01**	IceAGE	983	DZMB-HH-68412		Bodotriidae	*Bathycuma brevirostre*	1	**ICE1-Bod004**
**Bod01**	IceAGE	983	DZMB-HH-68412		Bodotriidae	*Bathycuma brevirostre*	4	
**Bod01**	IceAGE	1057	DZMB-HH-68430		Bodotriidae	*Bathycuma brevirostre*	1	
**Bod02**	IceAGE	1072	DZMB-HH-68359		Bodotriidae	*Bathycuma* sp. 1	1	
**Bod03**	IceAGE	1072	DZMB-HH-68361		Bodotriidae	Bodotriidae sp. 1	1	**ICE1-Bod003**
**Bod03**	IceAGE	1072	DZMB-HH-68384		Bodotriidae	Bodotriidae sp. 1	4	
**Bod04**	IceAGE	983	DZMB-HH-69392		Bodotriidae	Bodotriidae sp. 2	4	
**Bod05**	IceAGE	983	DZMB-HH-68432		Bodotriidae	*Cyclaspis longicaudata*	10	
**Bod05-A**	UoB	11.05.15-1	Bio material=4-6	MK613872.1	Bodotriidae	*Cyclaspis longicaudata*	1	**seq2**
**Bod05-A**	UoB	VI-22 2011	Bio material=7-8	MK613873.1	Bodotriidae	*Cyclaspis longicaudata*	1	**seq3**
**Bod05-B**	IceAGE	983	DZMB-HH-68410		Bodotriidae	*Cyclaspis longicaudata*	1	**ICE1-Bod001**
**Bod05-B**	IceAGE	983	DZMB-HH-68411		Bodotriidae	*Cyclaspis longicaudata*	1	**ICE1-Bod002**
**Bod06**	UoB	KV-09 2011	Bio material=146	MK613886.1	Bodotriidae	*Iphinoe serrata*	1	**seq4**
**Cer01**	IceAGE	1057	DZMB-HH-68388		Ceratocumatidae	*Cimmerius reticulatus*	1	**ICE1-Cer001**
**Cer01**	IceAGE	1057	DZMB-HH-68395		Ceratocumatidae	*Cimmerius reticulatus*	4	
**Cer01**	IceAGE	1072	DZMB-HH-68362		Ceratocumatidae	*Cimmerius reticulatus*	1	**ICE1-Cer002**
**Cer01**	IceAGE	1072	DZMB-HH-68349		Ceratocumatidae	*Cimmerius reticulatus*	1	**ICE1-Cer003**
**Cer01**	IceAGE	1072	DZMB-HH-68353		Ceratocumatidae	*Cimmerius reticulatus*	5	
**Cer01**	IceAGE	1072	DZMB-HH-68383		Ceratocumatidae	*Cimmerius reticulatus*	1	
**Dia01**	UoB	09.01.28-2	Bio material=160409-8	MK613898.1	Diastylidae	*Diastylis cornuta*	1	**seq25**
**Dia01**	UoB	VGPT1-22 2009	Bio material=9-13	MK613897.1	Diastylidae	*Diastylis cornuta*	1	**seq26**
**Dia02**	IceAGE	1136	DZMB-HH-68286		Diastylidae	*Diastylis echinata*	1	
**Dia03**	UoB	UNIS 2007-129	Bio material=031109-12	MK613904.1	Diastylidae	*Diastylis goodsiri*	1	**seq31**
**Dia04**	UoB	BS 14-19	Bio material=21-22	MK613901.1	Diastylidae	*Diastylis laevis*	1	**seq33**
**Dia05**	UoB	BS 14-19	Bio material=26-28	MK613911.1	Diastylidae	*Diastylis lucifera*	1	**seq35**
**Dia06**	IceAGE	1144	DZMB-HH-68295		Diastylidae	*Diastylis polaris*	1	**ICE1-Dia010**
**Dia06**	IceAGE	1144	DZMB-HH-68297		Diastylidae	*Diastylis polaris*	1	**ICE1-Dia016**
**Dia06**	IceAGE	1144	DZMB-HH-34137		Diastylidae	*Diastylis polaris*	9	
**Dia06**	IceAGE	1144	DZMB-HH-68303		Diastylidae	*Diastylis polaris*	3	
**Dia06**	IceAGE	1153	DZMB-HH-68294		Diastylidae	*Diastylis polaris*	1	ICE1-Dia020
**Dia06**	IceAGE	1153	DZMB-HH-34047		Diastylidae	*Diastylis polaris*	1	
**Dia06**	IceAGE	1155	DZMB-HH-38633		Diastylidae	*Diastylis polaris*	1	
**Dia06**	IceAGE	1184	DZMB-HH-68262		Diastylidae	*Diastylis polaris*	1	**ICE1-Dia003**
**Dia06**	IceAGE	1184	DZMB-HH-68263		Diastylidae	*Diastylis polaris*	1	**ICE1-Dia006**
**Dia06**	IceAGE	1184	DZMB-HH-68234		Diastylidae	*Diastylis polaris*	1	**ICE1-Dia009**
**Dia06**	IceAGE	1184	DZMB-HH-33632		Diastylidae	*Diastylis polaris*	27	
**Dia06**	IceAGE	1191	DZMB-HH-68259		Diastylidae	*Diastylis polaris*	1	**ICE1-Dia019**
**Dia06**	IceAGE	1191	DZMB-HH-68260		Diastylidae	*Diastylis polaris*	1	
**Dia06**	UoB	H2DEEP-RP-1	Bio material=29-31	MK613902.1	Diastylidae	*Diastylis polaris*	1	**seq39**
**Dia06**	MAREANO	R488-379, BT	Bio material=32-33	MK613903.1	Diastylidae	*Diastylis polaris*	1	**seq40**
**Dia07**	UoB	UNIS 2009-73	Bio material=031109-19	MK613905.1	Diastylidae	*Diastylis rathkei*	1	**seq36**
**Dia08**	IceAGE	1136	DZMB-HH-68285		Diastylidae	*Diastylis spinulosa*	1	
**Dia08**	UoB	UNIS 2007-140	Bio material=031109-16	MK613906.1	Diastylidae	*Diastylis* cf. *spinulosa*	1	**seq38**
**Dia09**	UoB	11.05.15-1	Bio material=35-37	MK613899.1	Diastylidae	*Diastylis tumida*	1	**seq42**
**Dia09**	MAREANO	R721-126, RP	Bio material=152-153	MK613900.1	Diastylidae	*Diastylis tumida*	1	**seq43**
**Dia10**	IceAGE	983	DZMB-HH-68413		Diastylidae	*Diastyloides atlanticus*	1	**ICE1-Dia011**
**Dia10**	IceAGE	983	DZMB-HH-68434		Diastylidae	*Diastyloides atlanticus*	1	**ICE1-Dia024**
**Dia10**	IceAGE	983	DZMB-HH-68440		Diastylidae	*Diastyloides atlanticus*	1	
**Dia10**	IceAGE	983	DZMB-HH-68428		Diastylidae	*Diastyloides atlanticus*	24	
**Dia11**	UoB	11.03.11-2	Bio material=D4-D6	MK613910.1	Diastylidae	*Diastyloides biplicatus*	1	**seq44**
**Dia12**	UoB	11.05.10-3	Bio material=38-40	MK613907.1	Diastylidae	*Diastyloides serratus*	1	**seq47**
**Dia12**	UoB	11.05.11-2C	Bio material=159, 174	MK613909.1	Diastylidae	*Diastyloides serratus*	1	**seq48**
**Dia12**	UoB	BS 82-147	Bio material=1004-1006	MK613908.1	Diastylidae	*Diastyloides serratus*	1	**seq49**
**Dia13**	IceAGE	983	DZMB-HH-68416		Diastylidae	*Diastyloides* sp. 1	1	ICE1-Dia022
**Dia14**	IceAGE	1072	DZMB-HH-68379		Diastylidae	*Leptostylis ampullacea*	1	
**Dia14**	IceAGE	1123	DZMB-HH-68456		Diastylidae	*Leptostylis ampullacea*	1	**ICE1-Dia018**
**Dia14**	IceAGE	1123	DZMB-HH-68462		Diastylidae	*Leptostylis ampullacea*	3	
**Dia14**	IceAGE	1123	DZMB-HH-68443		Diastylidae	*Leptostylis ampullacea*	1	**ICE1-Dia001**
**Dia14**	IceAGE	1123	DZMB-HH-68444		Diastylidae	*Leptostylis ampullacea*	1	ICE1-Dia004
**Dia14**	IceAGE	1123	DZMB-HH-68445		Diastylidae	*Leptostylis ampullacea*	1	**ICE1-Dia007**
**Dia14**	IceAGE	1123	DZMB-HH-39617		Diastylidae	*Leptostylis ampullacea*	9	
**Dia14**	IceAGE	1136	DZMB-HH-68266		Diastylidae	*Leptostylis ampullacea*	1	**ICE1-Dia002**
**Dia14**	IceAGE	1136	DZMB-HH-68267		Diastylidae	*Leptostylis ampullacea*	1	**ICE1-Dia005**
**Dia14**	IceAGE	1136	DZMB-HH-68268		Diastylidae	*Leptostylis ampullacea*	1	**ICE1-Dia008**
**Dia14**	IceAGE	1136	DZMB-HH-68291		Diastylidae	*Leptostylis ampullacea*	12	
**Dia14**	IceAGE	1144	DZMB-HH-68296		Diastylidae	*Leptostylis ampullacea*	1	**ICE1-Dia013**
**Dia14**	IceAGE	1144	DZMB-HH-68302		Diastylidae	*Leptostylis ampullacea*	9	
**Dia14**	IceAGE	1191	DZMB-HH-68258		Diastylidae	*Leptostylis ampullacea*	1	**ICE1-Dia014**
**Dia14**	IceAGE	1191	DZMB-HH-33005		Diastylidae	*Leptostylis ampullacea*	3	
**Dia15**	IceAGE	1136	DZMB-HH-68269		Diastylidae	*Leptostylis borealis*	1	**ICE1-Dia015**
**Dia15**	IceAGE	1136	DZMB-HH-68290		Diastylidae	*Leptostylis borealis*	2	
**Dia15**	IceAGE	1219	DZMB-HH-68403		Diastylidae	*Leptostylis borealis*	1	**ICE1-Dia017**
**Dia15**	IceAGE	1219	DZMB-HH-68407		Diastylidae	*Leptostylis borealis*	3	
**Dia16-A**	UoB	11.05.10-1	Bio material=41-43	MK613921.1	Diastylidae	*Leptostylis longimana*	1	**seq52**
**Dia16-A**	UoB	BS 82-147	Bio material=49-50	MK613922.1	Diastylidae	*Leptostylis longimana*	1	**seq53**
**Dia16-B**	PASCAL	22/3	DZMB-HH-63319		Diastylidae	*Leptostylis* cf. *longimana*	1	P-Dias005
**Dia16-B**	PASCAL	22/3	DZMB-HH-59193		Diastylidae	*Leptostylis* cf. *longimana*	1	P-Dias006
**Dia16-B**	PASCAL	24/5	DZMB-HH-63369		Diastylidae	*Leptostylis* cf. *longimana*	1	**P-Dias001**
**Dia16-B**	PASCAL	24/5	DZMB-HH-63370		Diastylidae	*Leptostylis* cf. *longimana*	1	**P-Dias002**
**Dia16-B**	PASCAL	24/5	DZMB-HH-59943		Diastylidae	*Leptostylis* cf. *longimana*	1	**P-Dias028**
**Dia16-B**	PASCAL	24/5	DZMB-HH-63371		Diastylidae	*Leptostylis* cf. *longimana*	1	**P-Dias032**
**Dia16-B**	PASCAL	24/5	DZMB-HH-59084		Diastylidae	*Leptostylis* cf. *longimana*	1	

Species ID	Project	Station	Sample ID	GenBank Accession	Family	Putative species	Specimen count	Sequence ID (Field ID)
**Dia16-B**	PASCAL	24/5	DZMB-HH-63358		Diastylidae	*Leptostylis* cf. *longimana*	1	
**Dia16-B**	PASCAL	24/5	DZMB-HH-63359		Diastylidae	*Leptostylis* cf. *longimana*	1	
**Dia16-B**	PASCAL	24/5	DZMB-HH-63360		Diastylidae	*Leptostylis* cf. *longimana*	1	
**Dia16-B**	PASCAL	24/5	DZMB-HH-63361		Diastylidae	*Leptostylis* cf. *longimana*	1	
**Dia16-B**	PASCAL	24/5	DZMB-HH-63362		Diastylidae	*Leptostylis* cf. *longimana*	1	
**Dia16-B**	PASCAL	24/5	DZMB-HH-63363		Diastylidae	*Leptostylis* cf. *longimana*	1	
**Dia16-B**	PASCAL	24/5	DZMB-HH-63364		Diastylidae	*Leptostylis* cf. *longimana*	1	
**Dia16-B**	PASCAL	24/5	DZMB-HH-63365		Diastylidae	*Leptostylis* cf. *longimana*	1	
**Dia16-B**	PASCAL	24/5	DZMB-HH-63366		Diastylidae	*Leptostylis* cf. *longimana*	1	
**Dia16-B**	PASCAL	24/5	DZMB-HH-63367		Diastylidae	*Leptostylis* cf. *longimana*	1	
**Dia16-B**	PASCAL	24/5	DZMB-HH-63368		Diastylidae	*Leptostylis* cf. *longimana*	1	
**Dia16-B**	PASCAL	24/5	DZMB-HH-63372		Diastylidae	*Leptostylis* cf. *longimana*	1	
**Dia16-B**	PASCAL	24/5	DZMB-HH-63373		Diastylidae	*Leptostylis* cf. *longimana*	1	
**Dia16-B**	PASCAL	24/5	DZMB-HH-63374		Diastylidae	*Leptostylis* cf. *longimana*	1	
**Dia16-B**	PASCAL	24/5	DZMB-HH-63375		Diastylidae	*Leptostylis* cf. *longimana*	1	
**Dia16-B**	PASCAL	24/5	DZMB-HH-63376		Diastylidae	*Leptostylis* cf. *longimana*	1	
**Dia16-B**	PASCAL	24/5	DZMB-HH-63377		Diastylidae	*Leptostylis* cf. *longimana*	1	
**Dia16-B**	PASCAL	24/5	DZMB-HH-63378		Diastylidae	*Leptostylis* cf. *longimana*	1	
**Dia16-B**	PASCAL	24/5	DZMB-HH-63379		Diastylidae	*Leptostylis* cf. *longimana*	1	
**Dia16-B**	PASCAL	24/5	DZMB-HH-63380		Diastylidae	*Leptostylis* cf. *longimana*	1	
**Dia16-B**	PASCAL	24/5	DZMB-HH-63381		Diastylidae	*Leptostylis* cf. *longimana*	1	
**Dia16-B**	PASCAL	24/5	DZMB-HH-63382		Diastylidae	*Leptostylis* cf. *longimana*	1	
**Dia16-B**	PASCAL	24/5	DZMB-HH-63383		Diastylidae	*Leptostylis* cf. *longimana*	1	
**Dia16-B**	PASCAL	24/5	DZMB-HH-63384		Diastylidae	*Leptostylis* cf. *longimana*	1	
**Dia16-B**	PASCAL	24/5	DZMB-HH-63385		Diastylidae	*Leptostylis* cf. *longimana*	1	
**Dia16-B**	PASCAL	24/5	DZMB-HH-63386		Diastylidae	*Leptostylis* cf. *longimana*	1	
**Dia16-B**	PASCAL	24/5	DZMB-HH-63387		Diastylidae	*Leptostylis* cf. *longimana*	1	
**Dia16-B**	PASCAL	24/5	DZMB-HH-63388		Diastylidae	*Leptostylis* cf. *longimana*	1	
**Dia16-B**	PASCAL	24/5	DZMB-HH-63389		Diastylidae	*Leptostylis* cf. *longimana*	1	
**Dia16-B**	PASCAL	24/5	DZMB-HH-63390		Diastylidae	*Leptostylis* cf. *longimana*	1	
**Dia16-B**	PASCAL	24/5	DZMB-HH-63391		Diastylidae	*Leptostylis* cf. *longimana*	1	
**Dia16-B**	PASCAL	24/5	DZMB-HH-63392		Diastylidae	*Leptostylis* cf. *longimana*	1	
**Dia16-B**	PASCAL	24/5	DZMB-HH-63393		Diastylidae	*Leptostylis* cf. *longimana*	1	
**Dia16-B**	PASCAL	24/5	DZMB-HH-63394		Diastylidae	*Leptostylis* cf. *longimana*	1	
**Dia16-B**	PASCAL	24/5	DZMB-HH-63395		Diastylidae	*Leptostylis* cf. *longimana*	1	
**Dia16-B**	PASCAL	24/5	DZMB-HH-63396		Diastylidae	*Leptostylis* cf. *longimana*	1	
**Dia16-B**	PASCAL	24/5	DZMB-HH-63397		Diastylidae	*Leptostylis* cf. *longimana*	1	
**Dia16-B**	PASCAL	24/5	DZMB-HH-63398		Diastylidae	*Leptostylis* cf. *longimana*	1	
**Dia16-B**	PASCAL	25/5	DZMB-HH-59218		Diastylidae	*Leptostylis* cf. *longimana*	1	**P-Dias007**
**Dia16-B**	PASCAL	25/5	DZMB-HH-63320		Diastylidae	*Leptostylis* cf. *longimana*	1	P-Dias008
**Dia16-B**	PASCAL	29/4	DZMB-HH-63324		Diastylidae	*Leptostylis* cf. *longimana*	1	P-Dias012
**Dia16-B**	PASCAL	29/4	DZMB-HH-63326		Diastylidae	*Leptostylis* cf. *longimana*	1	P-Dias013
**Dia16-B**	PASCAL	29/4	DZMB-HH-63327		Diastylidae	*Leptostylis* cf. *longimana*	1	P-Dias014
**Dia16-B**	PASCAL	29/4	DZMB-HH-59404		Diastylidae	*Leptostylis* cf. *longimana*	1	
**Dia16-B**	PASCAL	29/7	DZMB-HH-59380		Diastylidae	*Leptostylis* cf. *longimana*	1	P-Dias009
**Dia16-B**	PASCAL	29/7	DZMB-HH-63321		Diastylidae	*Leptostylis* cf. *longimana*	1	P-Dias010
**Dia16-B**	PASCAL	29/7	DZMB-HH-63322		Diastylidae	*Leptostylis* cf. *longimana*	1	P-Dias011
**Dia16-B**	PASCAL	29/7	DZMB-HH-59469		Diastylidae	*Leptostylis* cf. *longimana*	1	P-Dias015
**Dia16-B**	PASCAL	29/7	DZMB-HH-63328		Diastylidae	*Leptostylis* cf. *longimana*	1	P-Dias016
**Dia16-B**	PASCAL	29/7	DZMB-HH-59484		Diastylidae	*Leptostylis* cf. *longimana*	1	P-Dias017
**Dia16-B**	PASCAL	29/7	DZMB-HH-63323		Diastylidae	*Leptostylis* cf. *longimana*	1	
**Dia16-B**	PASCAL	29/7	DZMB-HH-63329		Diastylidae	*Leptostylis* cf. *longimana*	1	
**Dia16-B**	PASCAL	30/1	DZMB-HH-63337		Diastylidae	*Leptostylis* cf. *longimana*	1	**P-Dias003**
**Dia16-B**	PASCAL	30/1	DZMB-HH-63340		Diastylidae	*Leptostylis* cf. *longimana*	1	P-Dias018
**Dia16-B**	PASCAL	30/1	DZMB-HH-63341		Diastylidae	*Leptostylis* cf. *longimana*	1	P-Dias019
**Dia16-B**	PASCAL	30/1	DZMB-HH-63342		Diastylidae	*Leptostylis* cf. *longimana*	1	P-Dias020
**Dia16-B**	PASCAL	30/1	DZMB-HH-59533		Diastylidae	*Leptostylis* cf. *longimana*	1	**P-Dias027**
**Dia16-B**	PASCAL	30/1	DZMB-HH-63343		Diastylidae	*Leptostylis* cf. *longimana*	1	**P-Dias031**
**Dia16-B**	PASCAL	30/1	DZMB-HH-63338		Diastylidae	*Leptostylis* cf. *longimana*	1	
**Dia16-B**	PASCAL	30/1	DZMB-HH-63339		Diastylidae	*Leptostylis* cf. *longimana*	1	
**Dia16-B**	PASCAL	30/1	DZMB-HH-63344		Diastylidae	*Leptostylis* cf. *longimana*	1	
**Dia16-B**	PASCAL	30/1	DZMB-HH-63345		Diastylidae	*Leptostylis* cf. *longimana*	1	
**Dia16-B**	PASCAL	30/1	DZMB-HH-63346		Diastylidae	*Leptostylis* cf. *longimana*	1	
**Dia16-B**	PASCAL	30/1	DZMB-HH-63347		Diastylidae	*Leptostylis* cf. *longimana*	1	
**Dia16-B**	PASCAL	30/1	DZMB-HH-63348		Diastylidae	*Leptostylis* cf. *longimana*	1	
**Dia16-B**	PASCAL	30/1	DZMB-HH-63349		Diastylidae	*Leptostylis* cf. *longimana*	1	
**Dia16-B**	PASCAL	30/1	DZMB-HH-63350		Diastylidae	*Leptostylis* cf. *longimana*	1	
**Dia16-B**	PASCAL	30/1	DZMB-HH-59569		Diastylidae	*Leptostylis* cf. *longimana*	1	
**Dia16-B**	PASCAL	30/1	DZMB-HH-63351		Diastylidae	*Leptostylis* cf. *longimana*	1	
**Dia16-B**	PASCAL	30/1	DZMB-HH-63352		Diastylidae	*Leptostylis* cf. *longimana*	1	
**Dia16-B**	PASCAL	30/1	DZMB-HH-59692		Diastylidae	*Leptostylis* cf. *longimana*	1	
**Dia16-B**	PASCAL	30/1	DZMB-HH-63353		Diastylidae	*Leptostylis* cf. *longimana*	1	
**Dia16-B**	PASCAL	32/3	DZMB-HH-63330		Diastylidae	*Leptostylis* cf. *longimana*	1	**P-Dias004**
**Dia16-B**	PASCAL	32/3	DZMB-HH-59494		Diastylidae	*Leptostylis* cf. *longimana*	1	P-Dias021
**Dia16-B**	PASCAL	32/3	DZMB-HH-63332		Diastylidae	*Leptostylis* cf. *longimana*	1	P-Dias022
**Dia16-B**	PASCAL	32/3	DZMB-HH-63333		Diastylidae	*Leptostylis* cf. *longimana*	1	P-Dias023
**Dia16-B**	PASCAL	32/3	DZMB-HH-63331		Diastylidae	*Leptostylis* cf. *longimana*	1	**P-Dias029**
**Dia16-B**	PASCAL	32/3	DZMB-HH-63334		Diastylidae	*Leptostylis* cf. *longimana*	1	**P-Dias030**
**Dia16-B**	PASCAL	32/3	DZMB-HH-63335		Diastylidae	*Leptostylis* cf. *longimana*	1	
**Dia16-B**	PASCAL	32/3	DZMB-HH-63336		Diastylidae	*Leptostylis* cf. *longimana*	1	
**Dia16-B**	PASCAL	32/3	DZMB-HH-59546		Diastylidae	*Leptostylis* cf. *longimana*	1	
**Dia16-B**	PASCAL	32/3	DZMB-HH-59641		Diastylidae	*Leptostylis* cf. *longimana*	1	

Species ID	Project	Station	Sample ID	GenBank Accession	Family	Putative species	Specimen count	Sequence ID (Field ID)
**Dia16-B**	PASCAL	32/4	DZMB-HH-59713		Diastylidae	*Leptostylis* cf. *longimana*	1	P-Dias024
**Dia16-B**	PASCAL	32/4	DZMB-HH-59732		Diastylidae	*Leptostylis* cf. *longimana*	1	P-Dias025
**Dia16-B**	PASCAL	32/4	DZMB-HH-63355		Diastylidae	*Leptostylis* cf. *longimana*	1	P-Dias026
**Dia16-B**	PASCAL	32/4	DZMB-HH-63354		Diastylidae	*Leptostylis* cf. *longimana*	1	
**Dia16-B**	PASCAL	32/4	DZMB-HH-59766		Diastylidae	*Leptostylis* cf. *longimana*	1	
**Dia16-B**	PASCAL	32/4	DZMB-HH-63356		Diastylidae	*Leptostylis* cf. *longimana*	1	
**Dia17**	IceAGE	983	DZMB-HH-68414		Diastylidae	*Leptostylis* sp. 1	1	**ICE1-Dia012**
**Dia17**	IceAGE	983	DZMB-HH-68418		Diastylidae	*Leptostylis* sp. 1	1	**ICE1-Dia025**
**Dia17**	IceAGE	983	DZMB-HH-68427		Diastylidae	*Leptostylis* sp. 1	13	
**Dia18**	IceAGE	983	DZMB-HH-68415		Diastylidae	*Leptostylis* sp. 2	2	ICE1-Dia021
**Dia18**	IceAGE	983	DZMB-HH-68417		Diastylidae	*Leptostylis* sp. 2	2	ICE1-Dia023
**Dia19**	IceAGE	983	DZMB-HH-68426		Diastylidae	*Makrokylindrus (Makrokylindrus) spiniventris*	1	
**Lam01**	Alaska	90626	Bio material=200912-9	MK613925.1	Lampropidae	*Alamprops augustinensis*	1	**seq87**
**Lam02**	IceAGE	983	DZMB-HH-68421		Lampropidae	*Chalarostylis elegans*	1	**ICE1-Lam009**
**Lam02**	IceAGE	983	DZMB-HH-68424		Lampropidae	*Chalarostylis elegans*	1	**ICE1-Lam017**
**Lam03**	IceAGE	1057	DZMB-HH-68401		Lampropidae	*Chalarostylis* sp. 1	1	
**Lam04**	UoB	BIOICE3669	Bio material=187-188, ma6	MK613924.1	Lampropidae	*Hemilamprops assimilis*	1	**seq80**
**Lam05**	IceAGE	983	DZMB-HH-38478		Lampropidae	*Hemilamprops* cf. *cristatus*	18	
**Lam05**	IceAGE	983	DZMB-HH-68439		Lampropidae	*Hemilamprops* cf. *cristatus*	1	
**Lam05**	IceAGE	1072	DZMB-HH-37441		Lampropidae	*Hemilamprops* cf. *cristatus*	2	
**Lam05**	IceAGE	1123	DZMB-HH-68455		Lampropidae	*Hemilamprops* cf. *cristatus*	1	
**Lam05**	IceAGE	1123	DZMB-HH-68461		Lampropidae	*Hemilamprops* cf. *cristatus*	1	
**Lam05-A**	IceAGE	1123	DZMB-HH-68446		Lampropidae	*Hemilamprops* cf. *cristatus*	1	**ICE1-Lam018**
**Lam05-A**	UoB	BS 86-151	Bio material=63-64	MK613913.1	Lampropidae	*Hemilamprops cristatus*	1	**seq81**
**Lam05-A**	UoB	BS 86-151	Bio material=65	MK613914.1	Lampropidae	*Hemilamprops cristatus*	1	**seq82**
**Lam05-B**	IceAGE	983	DZMB-HH-68420		Lampropidae	*Hemilamprops* cf. *cristatus*	1	**ICE1-Lam002**
**Lam05-B**	IceAGE	983	DZMB-HH-68436		Lampropidae	*Hemilamprops* cf. *cristatus*	1	**ICE1-Lam008**
**Lam06**	IceAGE	961	DZMB-HH-42070		Lampropidae	*Hemilamprops cf. diversus*	1	
**Lam06**	IceAGE	983	DZMB-HH-68419		Lampropidae	*Hemilamprops cf. diversus*	1	**ICE1-Lam001**
**Lam06**	IceAGE	983	DZMB-HH-68435		Lampropidae	*Hemilamprops cf. diversus*	1	**ICE1-Lam006**
**Lam06**	IceAGE	983	DZMB-HH-68422		Lampropidae	*Hemilamprops cf. diversus*	1	**ICE1-Lam010**
**Lam06**	IceAGE	983	DZMB-HH-68423		Lampropidae	*Hemilamprops cf. diversus*	1	**ICE1-Lam011**
**Lam06**	IceAGE	983	DZMB-HH-68429		Lampropidae	*Hemilamprops cf. diversus*	254	
**Lam06**	IceAGE	983	DZMB-HH-68441		Lampropidae	*Hemilamprops cf. diversus*	3	
**Lam07**	IceAGE	1072	DZMB-HH-68363		Lampropidae	*Hemilamprops pterini*	1	**ICE1-Lam005**
**Lam07**	IceAGE	1072	DZMB-HH-68364		Lampropidae	*Hemilamprops pterini*	1	**ICE1-Lam013**
**Lam07**	IceAGE	1072	DZMB-HH-37397		Lampropidae	*Hemilamprops pterini*	6	
**Lam07**	IceAGE	1072	DZMB-HH-68374		Lampropidae	*Hemilamprops pterini*	3	
**Lam08**	UoB	BS 28-44	Bio material=66-67	MK613923.1	Lampropidae	*Hemilamprops roseus*	1	**seq83**
**Lam09**	IceAGE	1072	DZMB-HH-68375		Lampropidae	*Hemilamprops* sp. 1 (juv.)	1	
**Lam09**	IceAGE	1123	DZMB-HH-68452		Lampropidae	*Hemilamprops* sp. 1 (juv.)	1	
**Lam10**	IceAGE	1057	DZMB-HH-68392		Lampropidae	*Hemilamprops* sp. 2	1	
**Lam10**	IceAGE	1072	DZMB-HH-68365		Lampropidae	*Hemilamprops* sp. 2	1	**ICE1-Lam015**
**Lam10**	IceAGE	1072	DZMB-HH-68377		Lampropidae	*Hemilamprops* sp. 2	1	
**Lam11**	IceAGE	1123	DZMB-HH-68458		Lampropidae	*Hemilamprops uniplicatus*	1	
**Lam11**	IceAGE	1136	DZMB-HH-68270		Lampropidae	*Hemilamprops uniplicatus*	1	**ICE1-Lam003**
**Lam11**	IceAGE	1136	DZMB-HH-33744		Lampropidae	*Hemilamprops uniplicatus*	6	
**Lam11**	UoB	11.05.15-1	Bio material=68-70	MK613915.1	Lampropidae	*Hemilamprops uniplicatus*	1	**seq84**
**Lam11**	UoB	SFND-08R 2011	Bio material=71-72	MK613916.1	Lampropidae	*Hemilamprops uniplicatus*	1	**seq85**
**Lam11**	IceAGE	1123	DZMB-HH-68451		Lampropidae	*Hemilamprops* cf. *uniplicatus*	1	
**Lam12**	UoB	VGPT1-22 2009	Bio material=61-62	MK613917.1	Lampropidae	*Mesolamprops denticulatus*	1	**seq88**
**Lam13**	IceAGE	1057	DZMB-HH-68393		Lampropidae	*Platysympus typicus*	1	
**Lam13**	IceAGE	1072	DZMB-HH-68366		Lampropidae	*Platysympus typicus*	1	**ICE1-Lam016**
**Lam13**	IceAGE	1072	DZMB-HH-68351		Lampropidae	*Platysympus typicus*	2	
**Lam13**	IceAGE	1072	DZMB-HH-68381		Lampropidae	*Platysympus typicus*	1	
**Lam13**	IceAGE	1136	DZMB-HH-68271		Lampropidae	*Platysympus typicus*	1	**ICE1-Lam004**
**Lam13**	IceAGE	1136	DZMB-HH-68272		Lampropidae	*Platysympus typicus*	1	ICE1-Lam012
**Lam13**	IceAGE	1136	DZMB-HH-68292		Lampropidae	*Platysympus typicus*	4	
**Lam13**	UoB	UNIS 2009-71	Bio material=031109-15	MK613918.1	Lampropidae	*Platysympus typicus*	1	**seq89**
**Lam13**	MAREANO	R814-22, RP	Bio material=ma14	MK613919.1	Lampropidae	*Platysympus typicus*	1	**seq90**
**Lam14**	IceAGE	983	DZMB-HH-68431		Lampropidae	*Platytyphlops semiornatus*	3	
**Lam14**	IceAGE	1072	DZMB-HH-68357		Lampropidae	*Platytyphlops semiornatus*	3	
**Leu01**	UoB	BS 75-135	Bio material=79-82	MK613870.1	Leuconidae	*Eudorella emarginata*	1	**seq59**
**Leu02**	UoB	BS 34-56	Bio material=88-93	MK613887.1	Leuconidae	*Eudorella hirsuta*	1	**seq62**
**Leu02**	UoB	11.03.09-1	Bio material=94-96, 102	MK613888.1	Leuconidae	*Eudorella hirsuta*	1	**seq63**
**Leu03**	IceAGE	983	DZMB-HH-68425		Leuconidae	*Eudorella* sp. 1	1	ICE1-Leu015
**Leu03**	IceAGE	983	DZMB-HH-69393		Leuconidae	*Eudorella* sp. 1	2	
**Leu04-A**	UoB	BS 28-44	Bio material=97, 99	MK613881.1	Leuconidae	*Eudorella truncatula*	1	**seq64**
**Leu04-A**	UoB	BS 75-135	Bio material=200	MK613882.1	Leuconidae	*Eudorella truncatula*	1	**seq67**
**Leu04-B**	UoB	11.01.19.1	Bio material=100-101	MK613884.1	Leuconidae	*Eudorella truncatula*	1	**seq65**
**Leu04-B**	UoB	11.01.21-1	Bio material=1007-1008	MK613883.1	Leuconidae	*Eudorella truncatula*	1	**seq68**
**Leu04-C**	MAREANO	R754-132, RP	Bio material=ma5	MK613885.1	Leuconidae	*Eudorella truncatula*	1	**seq69**
**Leu05**	IceAGE	1123	DZMB-HH-68447		Leuconidae	*Leucon (Alytoleucon) pallidus*	1	ICE1-Leu014
**Leu05**	IceAGE	1123	DZMB-HH-68457		Leuconidae	*Leucon (Alytoleucon) pallidus*	1	**ICE1-Leu019**
**Leu05**	IceAGE	1123	DZMB-HH-68453		Leuconidae	*Leucon (Alytoleucon) pallidus*	3	
**Leu05**	IceAGE	1123	DZMB-HH-68459		Leuconidae	*Leucon (Alytoleucon) pallidus*	1	
**Leu05**	IceAGE	1136	DZMB-HH-68274		Leuconidae	*Leucon (Alytoleucon) pallidus*	1	**ICE1-Leu002**
**Leu05**	IceAGE	1136	DZMB-HH-68276		Leuconidae	*Leucon (Alytoleucon) pallidus*	1	**ICE1-Leu005**
**Leu05**	IceAGE	1136	DZMB-HH-68278		Leuconidae	*Leucon (Alytoleucon) pallidus*	1	**ICE1-Leu008**
**Leu05**	IceAGE	1136	DZMB-HH-68283		Leuconidae	*Leucon (Alytoleucon) pallidus*	11	
**Leu05**	IceAGE	1144	DZMB-HH-68298		Leuconidae	*Leucon (Alytoleucon) pallidus*	1	**ICE1-Leu003**
**Leu05**	IceAGE	1144	DZMB-HH-68299		Leuconidae	*Leucon (Alytoleucon) pallidus*	1	**ICE1-Leu006**

Species ID	Project	Station	Sample ID	GenBank Accession	Family	Putative species	Specimen count	Sequence ID (Field ID)
**Leu05**	IceAGE	1144	DZMB-HH-68300		Leuconidae	*Leucon (Alytoleucon) pallidus*	1	**ICE1-Leu009**
**Leu05**	IceAGE	1144	DZMB-HH-68301		Leuconidae	*Leucon (Alytoleucon) pallidus*	10	
**Leu05**	IceAGE	1219	DZMB-HH-68404		Leuconidae	*Leucon (Alytoleucon) pallidus*	1	**ICE1-Leu010**
**Leu05**	IceAGE	1219	DZMB-HH-38178		Leuconidae	*Leucon (Alytoleucon) pallidus*	5	
**Leu05**	UoB	BS 82-147	Bio material=1001-1003	MK613892.1	Leuconidae	*Leucon (Alytoleucon) pallidus*	1	**seq77**
**Leu05**	PASCAL	24/5	DZMB-HH-63416		Leuconidae	*Leucon (Alytoleucon) pallidus*	1	P-Leu001
**Leu05**	UoB	11.01.19.1	Bio material=117-119	MK613891.1	Leuconidae	*Leucon (Alytoleucon) pallidus*	1	**seq78**
**Leu06**	IceAGE	983	DZMB-HH-38573		Leuconidae	*Leucon (Crymoleucon) tener*	1	
**Leu07**	UoB	BS 22-32	Bio material=103-108	MK613889.1	Leuconidae	*Leucon (Leucon) acutirostris*	1	**seq70**
**Leu08**	UoB	09.01.28-2	Bio material=109-111	MK613895.1	Leuconidae	*Leucon (Leucon) nathorsti*	1	**seq72**
**Leu08**	UoB	BS 75-135	Bio material=112-114	MK613893.1	Leuconidae	*Leucon (Leucon) nathorsti*	1	**seq73**
**Leu09**	UoB	UNIS 2009-27	Bio material=031109-9	MK613894.1	Leuconidae	*Leucon (Leucon)* aff. *nathorsti*	1	**seq75**
**Leu10**	UoB	UNIS2009-4	Bio material=115-116	MK613890.1	Leuconidae	*Leucon (Leucon) nasicoides*	1	**seq74**
**Leu11**	IceAGE	1123	DZMB-HH-68448		Leuconidae	*Leucon (Leucon) profundus*	1	ICE1-Leu016
**Leu11**	IceAGE	1123	DZMB-HH-68454		Leuconidae	*Leucon (Leucon) profundus*	2	
**Leu11**	IceAGE	1123	DZMB-HH-68460		Leuconidae	*Leucon (Leucon) profundus*	1	
**Leu11**	IceAGE	1136	DZMB-HH-68273		Leuconidae	*Leucon (Leucon) profundus*	1	**ICE1-Leu001**
**Leu11**	IceAGE	1136	DZMB-HH-68275		Leuconidae	*Leucon (Leucon) profundus*	1	**ICE1-Leu004**
**Leu11**	IceAGE	1136	DZMB-HH-68277		Leuconidae	*Leucon (Leucon) profundus*	1	**ICE1-Leu007**
**Leu11**	IceAGE	1136	DZMB-HH-68284		Leuconidae	*Leucon (Leucon) profundus*	68	
**Leu11**	IceAGE	1219	DZMB-HH-68405		Leuconidae	*Leucon (Leucon) profundus*	1	ICE1-Leu012
**Leu11**	IceAGE	1219	DZMB-HH-68406		Leuconidae	*Leucon (Leucon) profundus*	4	
**Leu11**	IceAGE	1136	DZMB-HH-68279		Leuconidae	*Leucon (Leucon)* cf. *profundus*	1	ICE1-Leu017
**Leu11**	IceAGE	1136	DZMB-HH-68293		Leuconidae	*Leucon (Leucon)* cf. *profundus*	1	
**Leu11**	IceAGE	1219	DZMB-HH-68408		Leuconidae	*Leucon (Leucon)* cf. *profundus*	1	
**Leu12**	IceAGE	983	DZMB-HH-68438		Leuconidae	*Leucon (Leucon)* cf. *robustus*	1	
**Leu13**	IceAGE	983	DZMB-HH-68437		Leuconidae	*Leucon (Macrauloleucon) siphonatus*	1	
**Leu14**	IceAGE	1123	DZMB-HH-68449		Leuconidae	*Leucon (Macrauloleucon) spinulosus*	1	**ICE1-Leu018**
**Leu14**	IceAGE	1123	DZMB-HH-68450		Leuconidae	*Leucon (Macrauloleucon) spinulosus*	1	
**Leu14**	IceAGE	1184	DZMB-HH-68265		Leuconidae	*Leucon (Macrauloleucon) spinulosus*	1	
**Leu14**	IceAGE	1191	DZMB-HH-68261		Leuconidae	*Leucon (Macrauloleucon) spinulosus*	1	
**Leu14**	IceAGE	1057	DZMB-HH-68391		Leuconidae	*Leucon (Macrauloleucon)* cf. *spinulosus*	1	
**Leu15**	IceAGE	1072	DZMB-HH-68378		Leuconidae	*Leucon* sp. 1	1	
**Nan01**	IceAGE	1057	DZMB-HH-68399		Nannastacidae	*Campylaspides* sp. 1	1	
**Nan02**	IceAGE	1057	DZMB-HH-68396		Nannastacidae	*Campylaspis alba*	1	
**Nan03**	UoB	BS 86-151	Bio material=120-122	MK613876.1	Nannastacidae	*Campylaspis costata*	1	**seq6**
**Nan04**	UoB	BS 34-56	Bio material=1-3	MK613874.1	Nannastacidae	*Campylaspis globosa*	1	**seq9**
**Nan04**	IceAGE	1057	DZMB-HH-68390		Nannastacidae	*Campylaspis globosa*	1	**ICE1-Nann014**
**Nan04**	IceAGE	1057	DZMB-HH-68398		Nannastacidae	*Campylaspis globosa*	1	
**Nan05**	IceAGE	1057	DZMB-HH-68389		Nannastacidae	*Campylaspis horrida*	1	**ICE1-Nann013**
**Nan05**	IceAGE	1057	DZMB-HH-68397		Nannastacidae	*Campylaspis horrida*	1	
**Nan05**	MAREANO	R721-126, RP	Bio material=123	MK613877.1	Nannastacidae	*Campylaspis horrida*	1	**seq10**
**Nan06**	PASCAL	24/5	DZMB-HH-63414		Nannastacidae	*Campylaspis intermedia*	1	**P-Nann009**
**Nan06**	PASCAL	24/5	DZMB-HH-63415		Nannastacidae	*Campylaspis intermedia*	1	P-Nann010
**Nan06**	IceAGE	1072	DZMB-HH-68360		Nannastacidae	*Campylaspis cf. intermedia*	1	
**Nan07**	PASCAL	22/3	DZMB-HH-59125		Nannastacidae	*Campylaspis rubicunda*	1	P-Nann005
**Nan07**	PASCAL	24/5	DZMB-HH-63399		Nannastacidae	*Campylaspis rubicunda*	1	**P-Nann001**
**Nan07**	PASCAL	24/5	DZMB-HH-63400		Nannastacidae	*Campylaspis rubicunda*	1	**P-Nann002**
**Nan07**	PASCAL	24/5	DZMB-HH-63401		Nannastacidae	*Campylaspis rubicunda*	1	**P-Nann003**
**Nan07**	PASCAL	24/5	DZMB-HH-63402		Nannastacidae	*Campylaspis rubicunda*	1	**P-Nann004**
**Nan07**	PASCAL	24/5	DZMB-HH-63405		Nannastacidae	*Campylaspis rubicunda*	1	**P-Nann006**
**Nan07**	PASCAL	24/5	DZMB-HH-63406		Nannastacidae	*Campylaspis rubicunda*	1	P-Nann007
**Nan07**	PASCAL	24/5	DZMB-HH-63407		Nannastacidae	*Campylaspis rubicunda*	1	P-Nann008
**Nan07**	PASCAL	24/5	DZMB-HH-59833		Nannastacidae	*Campylaspis rubicunda*	1	**P-Nann011**
**Nan07**	PASCAL	24/5	DZMB-HH-63357		Nannastacidae	*Campylaspis rubicunda*	1	**P-Nann012**
**Nan07**	PASCAL	24/5	DZMB-HH-63403		Nannastacidae	*Campylaspis rubicunda*	1	
**Nan07**	PASCAL	24/5	DZMB-HH-63404		Nannastacidae	*Campylaspis rubicunda*	1	
**Nan07**	PASCAL	24/5	DZMB-HH-63408		Nannastacidae	*Campylaspis rubicunda*	1	
**Nan07**	PASCAL	24/5	DZMB-HH-63409		Nannastacidae	*Campylaspis rubicunda*	1	
**Nan07**	PASCAL	24/5	DZMB-HH-63410		Nannastacidae	*Campylaspis rubicunda*	1	
**Nan07**	PASCAL	24/5	DZMB-HH-63411		Nannastacidae	*Campylaspis rubicunda*	1	
**Nan07**	PASCAL	24/5	DZMB-HH-63412		Nannastacidae	*Campylaspis rubicunda*	1	
**Nan07**	PASCAL	24/5	DZMB-HH-63413		Nannastacidae	*Campylaspis rubicunda*	1	
**Nan08**	IceAGE	1136	DZMB-HH-68289		Nannastacidae	*Campylaspis* sp. 1	1	
**Nan09**	IceAGE	1057	DZMB-HH-68394		Nannastacidae	*Campylaspis* sp. 2	1	
**Nan09**	IceAGE	1072	DZMB-HH-68367		Nannastacidae	*Campylaspis* sp. 2	1	ICE1-Nann001
**Nan09**	IceAGE	1072	DZMB-HH-68368		Nannastacidae	*Campylaspis* sp. 2	1	ICE1-Nann003
**Nan09**	IceAGE	1072	DZMB-HH-68369		Nannastacidae	*Campylaspis* sp. 2	1	**ICE1-Nann005**
**Nan09**	IceAGE	1072	DZMB-HH-68371		Nannastacidae	*Campylaspis* sp. 2	1	ICE1-Nann010
**Nan09**	IceAGE	1072	DZMB-HH-68372		Nannastacidae	*Campylaspis* sp. 2	1	ICE1-Nann011
**Nan09**	IceAGE	1072	DZMB-HH-68350		Nannastacidae	*Campylaspis* sp. 2	1	ICE1-Nann015
**Nan09**	IceAGE	1072	DZMB-HH-68352		Nannastacidae	*Campylaspis* sp. 2	24	
**Nan09**	IceAGE	1072	DZMB-HH-68382		Nannastacidae	*Campylaspis* sp. 2	24	
**Nan10**	IceAGE	1136	DZMB-HH-68280		Nannastacidae	*Campylaspis sulcata*	1	**ICE1-Nann002**
**Nan10**	IceAGE	1136	DZMB-HH-68281		Nannastacidae	*Campylaspis sulcata*	1	**ICE1-Nann004**
**Nan10**	IceAGE	1136	DZMB-HH-68282		Nannastacidae	*Campylaspis sulcata*	1	**ICE1-Nann006**
**Nan10**	IceAGE	1136	DZMB-HH-68288		Nannastacidae	*Campylaspis sulcata*	19	
**Nan10**	UoB	11.05.15-1	Bio material=134-139	MK613875.1	Nannastacidae	*Campylaspis sulcata*	1	**seq14**
**Nan11**	UoB	11.05.15-1	Bio material=140-145	MK613878.1	Nannastacidae	*Campylaspis undata*	1	**seq21**
**Nan11**	IceAGE	1136	DZMB-HH-68287		Nannastacidae	*Campylaspis* cf. *undata*	1	
**Nan12**	IceAGE	1072	DZMB-HH-68373		Nannastacidae	*Cumella (Cumella)* cf. *decipiens*	1	ICE1-Nann012
**Nan12**	IceAGE	1072	DZMB-HH-68380		Nannastacidae	*Cumella (Cumella)* cf. *decipiens*	1	

Species ID	Project	Station	Sample ID	GenBank Accession	Family	Putative species	Specimen count	Sequence ID (Field ID)
**Nan13**	IceAGE	1010	DZMB-HH-40488		Nannastacidae	*Cumellopsis* cf. *puritani*	1	
**Nan14**	IceAGE	1072	DZMB-HH-68355		Nannastacidae	Nannastacidae sp. 1	2	
**Nan14**	IceAGE	1072	DZMB-HH-68387		Nannastacidae	Nannastacidae sp. 1	1	
**Nan15**	IceAGE	1072	DZMB-HH-68356		Nannastacidae	*Procampylaspis ommidion*	4	
**Nan15**	IceAGE	1072	DZMB-HH-68402		Nannastacidae	*Procampylaspis ommidion*	1	
**Nan16**	IceAGE	1072	DZMB-HH-69390		Nannastacidae	*Procampylaspis* sp. 1	9	
**Nan17**	IceAGE	1072	DZMB-HH-69388		Nannastacidae	*Styloptocuma erectum*	1	
**Nan17**	IceAGE	1072	DZMB-HH-68386		Nannastacidae	*Styloptocuma erectum*	1	
**Nan18**	IceAGE	1072	DZMB-HH-68370		Nannastacidae	*Styloptocuma gracillimum*	1	**ICE1-Nann008**
**Nan18**	IceAGE	1072	DZMB-HH-68354		Nannastacidae	*Styloptocuma gracillimum*	4	
**Nan18**	IceAGE	1072	DZMB-HH-68385		Nannastacidae	*Styloptocuma gracillimum*	3	
**Nan19**	IceAGE	1072	DZMB-HH-68358		Nannastacidae	*Styloptocuma* sp. 1	1	
**Nan20**	IceAGE	1072	DZMB-HH-69389		Nannastacidae	*Styloptocuma* sp. 2	2	
**Pse01**	UoB	UNIS 2009-36	Bio material=156-158	MK613871.1	Pseudocumatidae	*Petalosarsia declivis*	1	**seq5**
**Pse02**	IceAGE	1057	DZMB-HH-38031		Pseudocumatidae	*Pseudocuma* sp. 1	1	
