# Supplementary material for: Adding pieces to the puzzle: insights into diversity and distribution patterns of Cumacea (Crustacea: Peracarida) from the deep North Atlantic to the Arctic Ocean
Source: PeerJ. 2021 Nov 11;9:e12379. doi: 10.7717/peerj.12379 (PMC8590803; doi:10.7717/peerj.12379)
Supplement: Supplemental Information 12 — Uncorrected intra- and interspecific pairwise genetic distance range (p-distance) of putative species of the Leuconidae, delimited ABGD groups based on the applied threshold of P = 0.01–0.1 (12 groups) and the groups’ nearest neighbor. [file peerj-09-12379-s012.pdf]

ABGD Group (P = 0.01-0.1)	Putative species	*N*	Intra-specific min	Intra-specific max	Inter-specific min	Inter-specific max	Nearest neighbor (min *p*-distance)
Leu02	*Eudorella hirsuta*	2	0.00	0.00	0.24	0.35	Leu10
Leu04-A	*Eudorella truncatula* (seq 64/ 67)	2	0.00	0.00	0.21	0.38	Leu04-C
Leu04-B	*Eudorella truncatula* (seq 65/ 68)	2	0.01	0.01	0.17	0.37	Leu04-C
Leu04-C	*Eudorella truncatula* (seq 69)	1	0.00	0.00	0.17	0.36	Leu04-B
Leu11	*Leucon (Leucon) profundus*	3	0.00	0.00	0.22	0.36	Leu10
Leu05	*Leucon(Alytoleucon) pallidus*	7	0.00	0.00	0.21	0.34	Leu08
Leu14	*Leucon (Macrauloleucon) spinulosus*	1	0.00	0.00	0.32	0.38	Leu10, Leu05
	Leucon (Leucon) assimilis	2	0.00	0.00	0.22	0.35	Leu10
	Leucon (Crymoleucon) intermedius	2	0.01	0.01	0.29	0.38	Leu02
Leu08	*Leucon (Leucon) nathorsti/ L. (L.) aff. nathorsti*	3	0.00	0.01	0.21	0.35	Leu05
Leu10	*Leucon (Leucon) nasicoides*	1	0.00	0.00	0.22	0.33	Leu10, Le08
	Leucon sp.	1	0.00	0.00	0.28	0.38	Leu10

N = Number of sequences
