# Supplementary material for: Adding pieces to the puzzle: insights into diversity and distribution patterns of Cumacea (Crustacea: Peracarida) from the deep North Atlantic to the Arctic Ocean
Source: PeerJ. 2021 Nov 11;9:e12379. doi: 10.7717/peerj.12379 (PMC8590803; doi:10.7717/peerj.12379)
Supplement: Supplemental Information 13 [file peerj-09-12379-s013.pdf]

Leuconidae

		1	2	3	4	5	6	7	8	9	10	11	12	13	14	15	16	17	18	19	20	21	22	23	24	25	26	27
seq62 *Eudorella hirsuta*	**1**		0.00	0.31	0.32	0.31	0.31	0.29	0.28	0.27	0.28	0.28	0.28	0.28	0.27	0.27	0.35	0.27	0.26	0.26	0.30	0.29	0.28	0.28	0.24	0.29	0.27	0.31
seq63 *Eudorella hirsuta*	**2**	0.00		0.31	0.32	0.31	0.32	0.30	0.28	0.28	0.28	0.28	0.28	0.28	0.28	0.28	0.35	0.28	0.27	0.27	0.30	0.30	0.29	0.29	0.24	0.29	0.28	0.31
seq64 *Eudorella truncatula*	**3**	0.31	0.31		0.23	0.00	0.23	0.21	0.33	0.31	0.31	0.33	0.31	0.34	0.31	0.31	0.37	0.31	0.34	0.34	0.33	0.33	0.33	0.33	0.33	0.33	0.31	0.38
seq65 *Eudorella truncatula*	**4**	0.32	0.32	0.23		0.23	0.01	0.17	0.36	0.33	0.33	0.36	0.33	0.36	0.33	0.33	0.37	0.33	0.35	0.35	0.34	0.34	0.35	0.35	0.33	0.35	0.33	0.37
seq67 *Eudorella truncatula*	**5**	0.31	0.31	0.00	0.23		0.23	0.21	0.33	0.30	0.30	0.33	0.30	0.33	0.30	0.31	0.36	0.30	0.33	0.33	0.33	0.33	0.32	0.32	0.32	0.32	0.30	0.37
seq68 *Eudorella truncatula*	**6**	0.31	0.32	0.23	0.01	0.23		0.17	0.36	0.33	0.33	0.36	0.33	0.36	0.33	0.33	0.37	0.33	0.35	0.35	0.34	0.35	0.35	0.35	0.33	0.35	0.33	0.37
seq69 *Eudorella truncatula*	**7**	0.29	0.30	0.21	0.17	0.21	0.17		0.34	0.32	0.32	0.34	0.32	0.34	0.32	0.32	0.36	0.32	0.34	0.34	0.31	0.31	0.34	0.34	0.33	0.34	0.32	0.34
ICE1-Leu001 *Leucon profundus*	**8**	0.28	0.28	0.33	0.36	0.33	0.36	0.34		0.24	0.24	0.00	0.24	0.00	0.24	0.24	0.33	0.24	0.24	0.24	0.32	0.31	0.23	0.23	0.22	0.23	0.24	0.34
ICE1-Leu002 *Leucon pallidus*	**9**	0.27	0.28	0.31	0.33	0.30	0.33	0.32	0.24		0.00	0.24	0.00	0.25	0.00	0.00	0.34	0.00	0.23	0.23	0.31	0.31	0.22	0.21	0.23	0.22	0.00	0.34
ICE1-Leu003 *Leucon pallidus*	**10**	0.28	0.28	0.31	0.33	0.30	0.33	0.32	0.24	0.00		0.24	0.00	0.25	0.00	0.00	0.34	0.00	0.23	0.23	0.31	0.31	0.22	0.22	0.23	0.22	0.00	0.34
ICE1-Leu004 *Leucon profundus*	**11**	0.28	0.28	0.33	0.36	0.33	0.36	0.34	0.00	0.24	0.24		0.24	0.00	0.24	0.24	0.33	0.24	0.24	0.24	0.32	0.31	0.23	0.23	0.22	0.23	0.24	0.34
ICE1-Leu006 *Leucon pallidus*	**12**	0.28	0.28	0.31	0.33	0.30	0.33	0.32	0.24	0.00	0.00	0.24		0.25	0.00	0.00	0.34	0.00	0.23	0.23	0.31	0.31	0.22	0.21	0.23	0.22	0.00	0.34
ICE1-Leu007 *Leucon profundus*	**13**	0.28	0.28	0.34	0.36	0.33	0.36	0.34	0.00	0.25	0.25	0.00	0.25		0.25	0.25	0.33	0.25	0.25	0.25	0.33	0.32	0.24	0.24	0.23	0.24	0.24	0.33
ICE1-Leu009 *Leucon pallidus*	**14**	0.27	0.28	0.31	0.33	0.30	0.33	0.32	0.24	0.00	0.00	0.24	0.00	0.25		0.00	0.34	0.00	0.23	0.23	0.31	0.31	0.22	0.21	0.23	0.22	0.00	0.34
ICE1-Leu010 *Leucon pallidus*	**15**	0.27	0.28	0.31	0.33	0.31	0.33	0.32	0.24	0.00	0.00	0.24	0.00	0.25	0.00		0.33	0.00	0.23	0.23	0.31	0.31	0.22	0.21	0.23	0.22	0.00	0.34
ICE1-Leu018 *Leucon spinulosus*	**16**	0.35	0.35	0.37	0.37	0.36	0.37	0.36	0.33	0.34	0.34	0.33	0.34	0.33	0.34	0.33		0.34	0.32	0.32	0.38	0.37	0.33	0.34	0.33	0.33	0.34	0.34
ICE1-Leu019 *Leucon pallidus*	**17**	0.27	0.28	0.31	0.33	0.30	0.33	0.32	0.24	0.00	0.00	0.24	0.00	0.25	0.00	0.00	0.34		0.23	0.23	0.31	0.31	0.22	0.21	0.23	0.22	0.00	0.34
HQ450552 *Leucon assimilis*	**18**	0.26	0.27	0.34	0.35	0.33	0.35	0.34	0.24	0.23	0.23	0.24	0.23	0.25	0.23	0.23	0.32	0.23		0.00	0.31	0.30	0.23	0.23	0.22	0.23	0.23	0.31
HQ450553 *Leucon assimilis*	**19**	0.26	0.27	0.34	0.35	0.33	0.35	0.34	0.24	0.23	0.23	0.24	0.23	0.25	0.23	0.23	0.32	0.23	0.00		0.31	0.30	0.23	0.23	0.22	0.23	0.23	0.31
HQ450549 *Leucon intermedius*	**20**	0.30	0.30	0.33	0.34	0.33	0.34	0.31	0.32	0.31	0.31	0.32	0.31	0.33	0.31	0.31	0.38	0.31	0.31	0.31		0.01	0.32	0.32	0.31	0.32	0.31	0.32
HQ450550 *Leucon intermedius*	**21**	0.29	0.30	0.33	0.34	0.33	0.35	0.31	0.31	0.31	0.31	0.31	0.31	0.32	0.31	0.31	0.37	0.31	0.30	0.30	0.01		0.31	0.31	0.31	0.31	0.31	0.30
seq72 *Leucon nasica*	**22**	0.28	0.29	0.33	0.35	0.32	0.35	0.34	0.23	0.22	0.22	0.23	0.22	0.24	0.22	0.22	0.33	0.22	0.23	0.23	0.32	0.31		0.00	0.25	0.01	0.22	0.34
seq73 *Leucon nasica*	**23**	0.28	0.29	0.33	0.35	0.32	0.35	0.34	0.23	0.21	0.22	0.23	0.21	0.24	0.21	0.21	0.34	0.21	0.23	0.23	0.32	0.31	0.00		0.25	0.01	0.21	0.34
seq74 *Leucon nasicoides*	**24**	0.24	0.24	0.33	0.33	0.32	0.33	0.33	0.22	0.23	0.23	0.22	0.23	0.23	0.23	0.23	0.33	0.23	0.22	0.22	0.31	0.31	0.25	0.25		0.25	0.23	0.28
seq75 *Leucon nathorsti*	**25**	0.29	0.29	0.33	0.35	0.32	0.35	0.34	0.23	0.22	0.22	0.23	0.22	0.24	0.22	0.22	0.33	0.22	0.23	0.23	0.32	0.31	0.01	0.01	0.25		0.22	0.34
seq77 *Leucon pallidus*	**26**	0.27	0.28	0.31	0.33	0.30	0.33	0.32	0.24	0.00	0.00	0.24	0.00	0.24	0.00	0.00	0.34	0.00	0.23	0.23	0.31	0.31	0.22	0.21	0.23	0.22		0.33
HQ450554 *Leucon* sp.	**27**	0.31	0.31	0.38	0.37	0.37	0.37	0.34	0.34	0.34	0.34	0.34	0.34	0.33	0.34	0.34	0.34	0.34	0.31	0.31	0.32	0.30	0.34	0.34	0.28	0.34	0.33	
