# Supplementary material for: Adding pieces to the puzzle: insights into diversity and distribution patterns of Cumacea (Crustacea: Peracarida) from the deep North Atlantic to the Arctic Ocean
Source: PeerJ. 2021 Nov 11;9:e12379. doi: 10.7717/peerj.12379 (PMC8590803; doi:10.7717/peerj.12379)
Supplement: Supplemental Information 14 — Uncorrected intra- and interspecific pairwise genetic distance range (p-distance) of putative species of the cumacean families Bodotriidae and Nannastacidae, delimited ABGD groups based on the applied threshold of P = 0.01–0.08 (13 groups) and the groups’ nearest neighbor. [file peerj-09-12379-s014.pdf]

ABGD Group (P = 0.01-0.08)	Putative species	*N*	Intra-specific		Inter-specific		Nearest neighbor (min *p*-distance)
			min	max	min	max	
Nan03	*Campylaspis costata*	1	0.00	0.00	0.17	0.41	Nan05
Nan05	*Campylaspis horrida*	1	0.00	0.00	0.17	0.40	Nan03, Nan11
Nan11	*Campylaspis undata*	1	0.00	0.00	0.17	0.40	Nan05
Nan04	*Campylaspis globosa*	1	0.00	0.00	0.28	0.39	Nan05, Nan10
Nan10	*Campylaspis sulcata*	1	0.00	0.00	0.25	0.41	Nan07
Nan07	*Campylaspis rubicunda*	5	0.00	0.01	0.25	0.45	N06
	Atlantocuma sp.	1	0.00	0.00	0.31	0.41	B04, B05
Bod06	*Iphinoe serrata*	1	0.00	0.00	0.29	0.42	Bod05-A, -B
Bod05-A	*Cyclaspis longicaudata* (seq2)	1	0.00	0.00	0.08	0.43	Bod05-B
	Cyclaspis sp.	1	0.00	0.00	0.14	0.44	Bod05-B
Bod05-B	*Cyclaspis longicaudata* (ICE1-Bod001/ ICE1-Bod002)	2	0.00	0.00	0.08	0.45	Bod05-A
Bod03	Bodotriidae sp. 1	1	0.00	0.00	0.23	0.42	Bod04
Bod04	*Bathycuma brevirostre*	1	0.00	0.00	0.23	0.41	Bod03

N = Number of sequences
