# Supplementary material for: Adding pieces to the puzzle: insights into diversity and distribution patterns of Cumacea (Crustacea: Peracarida) from the deep North Atlantic to the Arctic Ocean
Source: PeerJ. 2021 Nov 11;9:e12379. doi: 10.7717/peerj.12379 (PMC8590803; doi:10.7717/peerj.12379)
Supplement: Supplemental Information 15 [file peerj-09-12379-s015.pdf]

Bodotriidae and Nannastacidae

		1	2	3	4	5	6	7	8	9	10	11	12	13	14	15	16	17	18	19
HQ450558 *Atlantocuma* sp.	**1**		0.41	0.38	0.41	0.40	0.38	0.36	0.32	0.31	0.31	0.31	0.34	0.33	0.37	0.38	0.38	0.39	0.38	0.38
seq6 *Campylaspis costata*	**2**	0.41		0.30	0.17	0.36	0.19	0.44	0.37	0.37	0.38	0.38	0.40	0.41	0.38	0.37	0.37	0.40	0.37	0.37
seq9 *Campylaspis globosa*	**3**	0.38	0.30		0.28	0.28	0.30	0.43	0.36	0.37	0.38	0.37	0.38	0.36	0.39	0.31	0.31	0.33	0.30	0.31
seq10 *Campylaspis horrida*	**4**	0.41	0.17	0.28		0.34	0.17	0.43	0.38	0.37	0.37	0.37	0.39	0.38	0.39	0.36	0.36	0.38	0.36	0.36
seq14 *Campylaspis sulcata*	**5**	0.40	0.36	0.28	0.34		0.34	0.42	0.40	0.41	0.39	0.39	0.41	0.35	0.41	0.25	0.25	0.26	0.25	0.25
seq21 *Campylaspis undata*	**6**	0.38	0.19	0.30	0.17	0.34		0.43	0.40	0.39	0.38	0.38	0.37	0.38	0.39	0.37	0.37	0.40	0.37	0.37
AJ388111 *Cumopsis fagei*	**7**	0.36	0.44	0.43	0.43	0.42	0.43		0.36	0.34	0.35	0.35	0.37	0.35	0.34	0.44	0.44	0.45	0.44	0.44
seq2 *Cyclaspis longicaudata*	**8**	0.32	0.37	0.36	0.38	0.40	0.40	0.36		0.17	0.08	0.08	0.29	0.29	0.28	0.42	0.42	0.44	0.42	0.42
HQ450557 *Cyclaspis* sp.	**9**	0.31	0.37	0.37	0.37	0.41	0.39	0.34	0.17		0.15	0.14	0.28	0.29	0.33	0.42	0.42	0.44	0.42	0.42
ICE1-Bod001 *Cyclaspis longicaudata*	**10**	0.31	0.38	0.38	0.37	0.39	0.38	0.35	0.08	0.15		0.00	0.28	0.28	0.28	0.41	0.41	0.45	0.41	0.41
ICE1-Bod002 *Cyclaspis longicaudata*	**11**	0.31	0.38	0.37	0.37	0.39	0.38	0.35	0.08	0.14	0.00		0.27	0.27	0.28	0.41	0.40	0.44	0.40	0.40
ICE1-Bod003 Bodotriidae sp. 1	**12**	0.34	0.40	0.38	0.39	0.41	0.37	0.37	0.29	0.28	0.28	0.27		0.24	0.32	0.41	0.41	0.42	0.41	0.41
ICE1-Bod004 *Bathycuma brevirostre*	**13**	0.33	0.41	0.36	0.38	0.35	0.38	0.35	0.29	0.29	0.28	0.27	0.24		0.34	0.37	0.37	0.40	0.37	0.37
seq4 *Iphinoe serrata*	**14**	0.37	0.38	0.39	0.39	0.41	0.39	0.34	0.28	0.33	0.28	0.28	0.32	0.34		0.41	0.41	0.42	0.41	0.41
P-Nann001 *Campylaspis rubicunda*	**15**	0.38	0.37	0.31	0.36	0.25	0.37	0.44	0.42	0.42	0.41	0.41	0.41	0.37	0.41		0.00	0.00	0.01	0.00
P-Nann002 *Campylaspis rubicunda*	**16**	0.38	0.37	0.31	0.36	0.25	0.37	0.44	0.42	0.42	0.41	0.40	0.41	0.37	0.41	0.00		0.00	0.01	0.00
P-Nann003 *Campylaspis rubicunda*	**17**	0.39	0.40	0.33	0.38	0.26	0.40	0.45	0.44	0.44	0.45	0.44	0.42	0.40	0.42	0.00	0.00		0.01	0.00
P-Nann004 *Campylaspis rubicunda*	**18**	0.38	0.37	0.30	0.36	0.25	0.37	0.44	0.42	0.42	0.41	0.40	0.41	0.37	0.41	0.01	0.01	0.01		0.01
P-Nann011 *Campylaspis rubicunda*	**19**	0.38	0.37	0.31	0.36	0.25	0.37	0.44	0.42	0.42	0.41	0.40	0.41	0.37	0.41	0.00	0.00	0.00	0.01	
