# Supplementary material for: Adding pieces to the puzzle: insights into diversity and distribution patterns of Cumacea (Crustacea: Peracarida) from the deep North Atlantic to the Arctic Ocean
Source: PeerJ. 2021 Nov 11;9:e12379. doi: 10.7717/peerj.12379 (PMC8590803; doi:10.7717/peerj.12379)
Supplement: Supplemental Information 16 — Uncorrected intra- and interspecific pairwise genetic distance range (p-distance) of putative species of the cumacean families Diastylidae and Pseudocumatidae, delimited ABGD groups based on the applied threshold of P = 0.01–0.1 (17 groups) and the groups’ nearest neighbor. [file peerj-09-12379-s016.pdf]

ABGD Group (P = 0.01-0.1)	Putative species	*N*	Intra-specific min	Intra-specific max	Inter-specific min	Inter-specific max	Nearest neighbor (min *p*-distance)
Dia01	*Diastylis cornuta*	2	0.01	0.01	0.15	0.33	Dia09
Dia03	*Diastylis goodsiri*	1	NA	NA	0.15	0.32	Dia07, Dia06
Dia04	*Diastylis laevis*	1	NA	NA	0.17	0.35	Dia01, D08
Dia05	*Diastylis lucifera*	1	NA	NA	0.23	0.31	Dia03, Dia06
Dia06	*Diastylis polaris*	8	0.00	0.00	0.15	0.33	Dia03, Dia07
Dia07	*Diastylis rathkei*	2	0.04	0.04	0.15	0.34	Dia03, Dia06
Dia08	*Diastylis spinulosa*	1	NA	NA	0.20	0.34	Dia07
Dia09	*Diastylis tumida*	2	0.00	0.00	0.15	0.32	Dia01
Dia11	*Diastyloides biplicatus*	1	NA	NA	0.18	0.37	Dia16-B
Dia12	*Diastyloides serratus*	3	0.00	0.00	0.18	0.37	Dia11
	Diastylopsis sp.	1	NA	NA	0.26	0.31	D17
Dia16-B	*Leptostylis* cf. *longimana/ Leptostylis ampullacea*	14	0.00	0.01	0.21	0.33	Dia17
Dia10	*Diastyloides atlanticus*	2	0.00	0.00	0.23	0.32	Dia08
Dia15	*Leptostylis borealis*	1	NA	NA	0.24	0.37	Dia16-B, Dia17
Dia16-A	*Leptostylis longimana* (seq52/seq53)	2	0.00	0.00	0.26	0.33	Dia16-B
Dia17	*Leptostylis* sp. 1	1	NA	NA	0.21	0.33	Dia16-B
Pse01	*Petalosarsia declivis*	1	NA	NA	0.29	0.34	Dia08, Dia09

N = Number of sequences
