# Supplementary material for: Adding pieces to the puzzle: insights into diversity and distribution patterns of Cumacea (Crustacea: Peracarida) from the deep North Atlantic to the Arctic Ocean
Source: PeerJ. 2021 Nov 11;9:e12379. doi: 10.7717/peerj.12379 (PMC8590803; doi:10.7717/peerj.12379)
Supplement: Supplemental Information 17 [file peerj-09-12379-s017.pdf]

Diastylidae and Pseudocumatidae

Sequence	#	1	2	3	4	5	6	7	8	9	10	11	12	13	14	15	16	17	18	19	20	21	22
seq25 *Diastylis cornuta*	**1**		0.01	0.21	0.18	0.24	0.20	0.20	0.23	0.22	0.22	0.16	0.16	0.28	0.30	0.30	0.30	0.29	0.29	0.29	0.22	0.29	0.22
seq26 *Diastylis cornuta*	**2**	0.01		0.21	0.17	0.24	0.21	0.21	0.22	0.22	0.22	0.15	0.15	0.29	0.30	0.30	0.30	0.28	0.29	0.29	0.22	0.29	0.22
seq31 *Diastylis goodsiri*	**3**	0.21	0.21		0.23	0.23	0.16	0.15	0.24	0.15	0.16	0.20	0.20	0.29	0.26	0.26	0.26	0.28	0.30	0.30	0.15	0.30	0.16
seq33 *Diastylis laevis*	**4**	0.18	0.17	0.23		0.27	0.24	0.23	0.25	0.26	0.26	0.17	0.17	0.29	0.27	0.26	0.26	0.28	0.31	0.31	0.26	0.31	0.26
seq35 *Diastylis lucifera*	**5**	0.24	0.24	0.23	0.27		0.25	0.24	0.25	0.24	0.24	0.25	0.25	0.29	0.30	0.30	0.30	0.28	0.29	0.28	0.24	0.29	0.24
HQ450555 *Diastylis rathkei*	**6**	0.20	0.21	0.16	0.24	0.25		0.04	0.21	0.16	0.16	0.22	0.22	0.27	0.26	0.26	0.26	0.29	0.30	0.30	0.16	0.30	0.16
seq36 *Diastylis rathkei*	**7**	0.20	0.21	0.15	0.23	0.24	0.04		0.20	0.15	0.15	0.22	0.22	0.29	0.26	0.26	0.26	0.30	0.31	0.30	0.15	0.31	0.15
seq38 *Diastylis spinulosa*	**8**	0.23	0.22	0.24	0.25	0.25	0.21	0.20		0.24	0.24	0.24	0.24	0.26	0.26	0.26	0.26	0.30	0.31	0.31	0.24	0.32	0.24
seq39 *Diastylis polaris*	**9**	0.22	0.22	0.15	0.26	0.24	0.16	0.15	0.24		0.00	0.23	0.23	0.26	0.28	0.28	0.28	0.30	0.30	0.29	0.00	0.30	0.00
seq40 *Diastylis polaris*	**10**	0.22	0.22	0.16	0.26	0.24	0.16	0.15	0.24	0.00		0.23	0.23	0.26	0.27	0.27	0.27	0.30	0.30	0.30	0.00	0.30	0.00
seq42 *Diastylis tumida*	**11**	0.16	0.15	0.20	0.17	0.25	0.22	0.22	0.24	0.23	0.23		0.00	0.28	0.28	0.28	0.28	0.28	0.31	0.31	0.23	0.31	0.23
seq43 *Diastylis tumida*	**12**	0.16	0.15	0.20	0.17	0.25	0.22	0.22	0.24	0.23	0.23	0.00		0.28	0.28	0.28	0.28	0.28	0.31	0.31	0.23	0.31	0.23
seq44 *Diastyloides biplicatus*	**13**	0.28	0.29	0.29	0.29	0.29	0.27	0.29	0.26	0.26	0.26	0.28	0.28		0.18	0.18	0.18	0.29	0.31	0.31	0.26	0.31	0.26
seq47 *Diastyloides serratus*	**14**	0.30	0.30	0.26	0.27	0.30	0.26	0.26	0.26	0.28	0.27	0.28	0.28	0.18		0.00	0.00	0.31	0.32	0.31	0.27	0.32	0.27
seq48 *Diastyloides serratus*	**15**	0.30	0.30	0.26	0.26	0.30	0.26	0.26	0.26	0.28	0.27	0.28	0.28	0.18	0.00		0.00	0.31	0.32	0.31	0.27	0.32	0.27
seq49 *Diastyloides serratus*	**16**	0.30	0.30	0.26	0.26	0.30	0.26	0.26	0.26	0.28	0.27	0.28	0.28	0.18	0.00	0.00		0.31	0.32	0.31	0.27	0.32	0.27
HQ450556 *Diastylopsis* sp.	**17**	0.29	0.28	0.28	0.28	0.28	0.29	0.30	0.30	0.30	0.30	0.28	0.28	0.29	0.31	0.31	0.31		0.28	0.28	0.30	0.28	0.30
ICE1-Dia001 *Leptostylis ampullacea*	**18**	0.29	0.29	0.30	0.31	0.29	0.30	0.31	0.31	0.30	0.30	0.31	0.31	0.31	0.32	0.32	0.32	0.28		0.00	0.29	0.01	0.29
ICE1-Dia002 *Leptostylis ampullacea*	**19**	0.29	0.29	0.30	0.31	0.28	0.30	0.30	0.31	0.29	0.30	0.31	0.31	0.31	0.31	0.31	0.31	0.28	0.00		0.29	0.01	0.29
ICE1-Dia003 *Diastylis polaris*	**20**	0.22	0.22	0.15	0.26	0.24	0.16	0.15	0.24	0.00	0.00	0.23	0.23	0.26	0.27	0.27	0.27	0.30	0.29	0.29		0.29	0.00
ICE1-Dia005 *Leptostylis ampullacea*	**21**	0.29	0.29	0.30	0.31	0.29	0.30	0.31	0.32	0.30	0.30	0.31	0.31	0.31	0.32	0.32	0.32	0.28	0.01	0.01	0.29		0.29
ICE1-Dia006 *Diastylis polaris*	**22**	0.22	0.22	0.16	0.26	0.24	0.16	0.15	0.24	0.00	0.00	0.23	0.23	0.26	0.27	0.27	0.27	0.30	0.29	0.29	0.00	0.29	
ICE1-Dia007 *Leptostylis ampullacea*	**23**	0.29	0.29	0.30	0.31	0.29	0.31	0.31	0.31	0.30	0.30	0.31	0.31	0.31	0.32	0.32	0.32	0.28	0.00	0.01	0.29	0.01	0.29
ICE1-Dia009 *Diastylis polaris*	**24**	0.22	0.22	0.16	0.26	0.24	0.16	0.15	0.24	0.00	0.00	0.23	0.23	0.26	0.28	0.27	0.27	0.30	0.30	0.29	0.00	0.30	0.00
ICE1-Dia010 *Diastylis polaris*	**25**	0.22	0.22	0.16	0.26	0.24	0.16	0.15	0.24	0.00	0.00	0.23	0.23	0.26	0.28	0.27	0.27	0.30	0.29	0.29	0.00	0.29	0.00
ICE1-Dia011 *Diastyloides atlanticus*	**26**	0.25	0.25	0.26	0.27	0.28	0.26	0.26	0.23	0.28	0.28	0.25	0.25	0.25	0.23	0.23	0.23	0.29	0.28	0.28	0.28	0.28	0.28
ICE1-Dia014 *Leptostylis ampullacea*	**27**	0.29	0.29	0.30	0.31	0.29	0.30	0.31	0.31	0.30	0.30	0.31	0.31	0.31	0.32	0.32	0.32	0.28	0.00	0.00	0.29	0.00	0.29
ICE1-Dia015 *Leptostylis borealis*	**28**	0.33	0.33	0.32	0.35	0.31	0.34	0.34	0.34	0.33	0.33	0.32	0.32	0.37	0.37	0.37	0.37	0.31	0.25	0.24	0.33	0.25	0.33
ICE1-Dia016 *Diastylis polaris*	**29**	0.22	0.22	0.16	0.26	0.23	0.16	0.15	0.25	0.00	0.00	0.23	0.23	0.26	0.28	0.28	0.28	0.28	0.30	0.30	0.00	0.30	0.00
ICE1-Dia018 *Leptostylis ampullacea*	**30**	0.29	0.29	0.30	0.31	0.29	0.30	0.30	0.31	0.29	0.30	0.31	0.31	0.31	0.31	0.31	0.31	0.28	0.00	0.01	0.29	0.01	0.29
ICE1-Dia019 *Diastylis polaris*	**31**	0.22	0.22	0.16	0.26	0.24	0.16	0.15	0.24	0.00	0.00	0.23	0.23	0.26	0.28	0.27	0.27	0.30	0.29	0.29	0.00	0.29	0.00
ICE1-Dia024 *Diastyloides atlanticus*	**32**	0.25	0.25	0.26	0.27	0.28	0.26	0.26	0.23	0.28	0.28	0.25	0.25	0.25	0.23	0.23	0.23	0.29	0.28	0.28	0.28	0.28	0.28
seq52 *Leptostylis longimana*	**33**	0.31	0.31	0.32	0.30	0.30	0.30	0.32	0.32	0.32	0.32	0.30	0.30	0.30	0.33	0.33	0.33	0.31	0.26	0.26	0.32	0.26	0.32
seq53 *Leptostylis longimana*	**34**	0.31	0.31	0.32	0.30	0.30	0.30	0.32	0.32	0.32	0.32	0.30	0.30	0.30	0.33	0.33	0.33	0.31	0.26	0.26	0.32	0.26	0.32
P-Dias002 *Leptostylis* cf. *longimana*	**35**	0.30	0.30	0.30	0.32	0.29	0.31	0.31	0.32	0.30	0.30	0.32	0.32	0.32	0.32	0.32	0.32	0.28	0.00	0.00	0.30	0.00	0.30
P-Dias003 *Leptostylis* cf. *longimana*	**36**	0.29	0.29	0.30	0.31	0.29	0.30	0.30	0.31	0.30	0.30	0.31	0.31	0.31	0.32	0.32	0.32	0.28	0.00	0.00	0.29	0.01	0.29
P-Dias004 *Leptostylis* cf. *longimana*	**37**	0.30	0.30	0.31	0.32	0.29	0.30	0.31	0.32	0.30	0.30	0.32	0.32	0.32	0.32	0.32	0.32	0.28	0.00	0.00	0.30	0.00	0.30
P-Dias027 *Leptostylis* cf. *longimana*	**38**	0.29	0.29	0.30	0.31	0.29	0.30	0.31	0.31	0.30	0.30	0.31	0.31	0.31	0.32	0.32	0.32	0.28	0.00	0.00	0.30	0.00	0.30
P-Dias028 *Leptostylis* cf. *longimana*	**39**	0.29	0.29	0.30	0.31	0.29	0.30	0.31	0.31	0.30	0.30	0.31	0.31	0.31	0.32	0.32	0.32	0.28	0.00	0.00	0.29	0.01	0.29
P-Dias030 *Leptostylis* cf. *longimana*	**40**	0.29	0.29	0.30	0.31	0.29	0.30	0.31	0.31	0.30	0.30	0.31	0.31	0.31	0.32	0.32	0.32	0.28	0.00	0.00	0.29	0.01	0.29
P-Dias031 *Leptostylis* cf. *longimana*	**41**	0.30	0.30	0.30	0.31	0.29	0.31	0.31	0.32	0.30	0.30	0.31	0.31	0.32	0.32	0.32	0.32	0.28	0.00	0.00	0.30	0.01	0.30
P-Dias032 *Leptostylis* cf. *longimana*	**42**	0.29	0.29	0.30	0.31	0.29	0.30	0.31	0.31	0.29	0.29	0.31	0.31	0.31	0.31	0.31	0.31	0.28	0.01	0.01	0.29	0.01	0.29
seq5 *Petalosarsia declivis*	**43**	0.31	0.31	0.32	0.32	0.31	0.30	0.30	0.29	0.31	0.31	0.29	0.29	0.31	0.31	0.31	0.31	0.32	0.33	0.33	0.31	0.33	0.31
ICE1-Dia025 *Leptostylis* sp. 1	**44**	0.29	0.29	0.32	0.29	0.28	0.30	0.30	0.30	0.30	0.30	0.30	0.30	0.31	0.33	0.32	0.32	0.26	0.22	0.21	0.29	0.22	0.29

Sequence	#	23	24	25	26	27	28	29	30	31	32	33	34	35	36	37	38	39	40	41	42	43	44
seq25 *Diastylis cornuta*	**1**	0.29	0.22	0.22	0.25	0.29	0.33	0.22	0.29	0.22	0.25	0.31	0.31	0.30	0.29	0.30	0.29	0.29	0.29	0.30	0.29	0.31	0.29
seq26 *Diastylis cornuta*	**2**	0.29	0.22	0.22	0.25	0.29	0.33	0.22	0.29	0.22	0.25	0.31	0.31	0.30	0.29	0.30	0.29	0.29	0.29	0.30	0.29	0.31	0.29
seq31 *Diastylis goodsiri*	**3**	0.30	0.16	0.16	0.26	0.30	0.32	0.16	0.30	0.16	0.26	0.32	0.32	0.30	0.30	0.31	0.30	0.30	0.30	0.30	0.30	0.32	0.32
seq33 *Diastylis laevis*	**4**	0.31	0.26	0.26	0.27	0.31	0.35	0.26	0.31	0.26	0.27	0.30	0.30	0.32	0.31	0.32	0.31	0.31	0.31	0.31	0.31	0.32	0.29
seq35 *Diastylis lucifera*	**5**	0.29	0.24	0.24	0.28	0.29	0.31	0.23	0.29	0.24	0.28	0.30	0.30	0.29	0.29	0.29	0.29	0.29	0.29	0.29	0.29	0.31	0.28
HQ450555 *Diastylis rathkei*	**6**	0.31	0.16	0.16	0.26	0.30	0.34	0.16	0.30	0.16	0.26	0.30	0.30	0.31	0.30	0.30	0.30	0.30	0.30	0.31	0.30	0.30	0.30
seq36 *Diastylis rathkei*	**7**	0.31	0.15	0.15	0.26	0.31	0.34	0.15	0.30	0.15	0.26	0.32	0.32	0.31	0.30	0.31	0.31	0.31	0.31	0.31	0.31	0.30	0.30
seq38 *Diastylis spinulosa*	**8**	0.31	0.24	0.24	0.23	0.31	0.34	0.25	0.31	0.24	0.23	0.32	0.32	0.32	0.31	0.32	0.31	0.31	0.31	0.32	0.31	0.29	0.30
seq39 *Diastylis polaris*	**9**	0.30	0.00	0.00	0.28	0.30	0.33	0.00	0.29	0.00	0.28	0.32	0.32	0.30	0.30	0.30	0.30	0.30	0.30	0.30	0.29	0.31	0.30
seq40 *Diastylis polaris*	**10**	0.30	0.00	0.00	0.28	0.30	0.33	0.00	0.30	0.00	0.28	0.32	0.32	0.30	0.30	0.30	0.30	0.30	0.30	0.30	0.29	0.31	0.30
seq42 *Diastylis tumida*	**11**	0.31	0.23	0.23	0.25	0.31	0.32	0.23	0.31	0.23	0.25	0.30	0.30	0.32	0.31	0.32	0.31	0.31	0.31	0.31	0.31	0.29	0.30
seq43 *Diastylis tumida*	**12**	0.31	0.23	0.23	0.25	0.31	0.32	0.23	0.31	0.23	0.25	0.30	0.30	0.32	0.31	0.32	0.31	0.31	0.31	0.31	0.31	0.29	0.30
seq44 *Diastyloides biplicatus*	**13**	0.31	0.26	0.26	0.25	0.31	0.37	0.26	0.31	0.26	0.25	0.30	0.30	0.32	0.31	0.32	0.31	0.31	0.31	0.32	0.31	0.31	0.31
seq47 *Diastyloides serratus*	**14**	0.32	0.28	0.28	0.23	0.32	0.37	0.28	0.31	0.28	0.23	0.33	0.33	0.32	0.32	0.32	0.32	0.32	0.32	0.32	0.31	0.31	0.33
seq48 *Diastyloides serratus*	**15**	0.32	0.27	0.27	0.23	0.32	0.37	0.28	0.31	0.27	0.23	0.33	0.33	0.32	0.32	0.32	0.32	0.32	0.32	0.32	0.31	0.31	0.32
seq49 *Diastyloides serratus*	**16**	0.32	0.27	0.27	0.23	0.32	0.37	0.28	0.31	0.27	0.23	0.33	0.33	0.32	0.32	0.32	0.32	0.32	0.32	0.32	0.31	0.31	0.32
HQ450556 *Diastylopsis* sp.	**17**	0.28	0.30	0.30	0.29	0.28	0.31	0.28	0.28	0.30	0.29	0.31	0.31	0.28	0.28	0.28	0.28	0.28	0.28	0.28	0.28	0.32	0.26
ICE1-Dia001 *Leptostylis ampullacea*	**18**	0.00	0.30	0.29	0.28	0.00	0.25	0.30	0.00	0.29	0.28	0.26	0.26	0.00	0.00	0.00	0.00	0.00	0.00	0.00	0.01	0.33	0.22
ICE1-Dia002 *Leptostylis ampullacea*	**19**	0.01	0.29	0.29	0.28	0.00	0.24	0.30	0.01	0.29	0.28	0.26	0.26	0.00	0.00	0.00	0.00	0.00	0.00	0.00	0.01	0.33	0.21
ICE1-Dia003 *Diastylis polaris*	**20**	0.29	0.00	0.00	0.28	0.29	0.33	0.00	0.29	0.00	0.28	0.32	0.32	0.30	0.29	0.30	0.30	0.29	0.29	0.30	0.29	0.31	0.29
ICE1-Dia005 *Leptostylis ampullacea*	**21**	0.01	0.30	0.29	0.28	0.00	0.25	0.30	0.01	0.29	0.28	0.26	0.26	0.00	0.01	0.00	0.00	0.01	0.01	0.01	0.01	0.33	0.22
ICE1-Dia006 *Diastylis polaris*	**22**	0.29	0.00	0.00	0.28	0.29	0.33	0.00	0.29	0.00	0.28	0.32	0.32	0.30	0.29	0.30	0.30	0.29	0.29	0.30	0.29	0.31	0.29
ICE1-Dia007 *Leptostylis ampullacea*	**23**		0.30	0.29	0.28	0.01	0.24	0.30	0.01	0.29	0.28	0.26	0.26	0.01	0.00	0.01	0.01	0.00	0.01	0.00	0.01	0.33	0.22
ICE1-Dia009 *Diastylis polaris*	**24**	0.30		0.00	0.28	0.30	0.33	0.00	0.29	0.00	0.28	0.32	0.32	0.30	0.30	0.30	0.30	0.30	0.30	0.30	0.29	0.31	0.29
ICE1-Dia010 *Diastylis polaris*	**25**	0.29	0.00		0.28	0.29	0.33	0.00	0.29	0.00	0.28	0.32	0.32	0.30	0.30	0.30	0.30	0.29	0.29	0.30	0.29	0.31	0.29
ICE1-Dia011 *Diastyloides atlanticus*	**26**	0.28	0.28	0.28		0.28	0.32	0.29	0.28	0.28	0.00	0.32	0.32	0.29	0.28	0.29	0.30	0.28	0.28	0.29	0.28	0.30	0.27
ICE1-Dia014 *Leptostylis ampullacea*	**27**	0.01	0.30	0.29	0.28		0.25	0.30	0.00	0.29	0.28	0.26	0.26	0.00	0.00	0.00	0.00	0.00	0.01	0.00	0.00	0.33	0.22
ICE1-Dia015 *Leptostylis borealis*	**28**	0.24	0.33	0.33	0.32	0.25		0.34	0.25	0.33	0.32	0.31	0.31	0.25	0.24	0.25	0.25	0.25	0.24	0.25	0.25	0.34	0.24
ICE1-Dia016 *Diastylis polaris*	**29**	0.30	0.00	0.00	0.29	0.30	0.34		0.30	0.00	0.29	0.32	0.32	0.30	0.30	0.30	0.30	0.30	0.30	0.30	0.29	0.30	0.30
ICE1-Dia018 *Leptostylis ampullacea*	**30**	0.01	0.29	0.29	0.28	0.00	0.25	0.30		0.29	0.28	0.26	0.26	0.00	0.00	0.00	0.00	0.00	0.01	0.00	0.01	0.33	0.22
ICE1-Dia019 *Diastylis polaris*	**31**	0.29	0.00	0.00	0.28	0.29	0.33	0.00	0.29		0.28	0.32	0.32	0.30	0.30	0.30	0.30	0.29	0.29	0.30	0.29	0.31	0.29
ICE1-Dia024 *Diastyloides atlanticus*	**32**	0.28	0.28	0.28	0.00	0.28	0.32	0.29	0.28	0.28		0.32	0.32	0.29	0.28	0.29	0.30	0.28	0.28	0.29	0.28	0.30	0.27
seq52 *Leptostylis longimana*	**33**	0.26	0.32	0.32	0.32	0.26	0.31	0.32	0.26	0.32	0.32		0.00	0.27	0.26	0.27	0.26	0.26	0.27	0.27	0.26	0.33	0.27
seq53 *Leptostylis longimana*	**34**	0.26	0.32	0.32	0.32	0.26	0.31	0.32	0.26	0.32	0.32	0.00		0.27	0.26	0.27	0.26	0.26	0.27	0.27	0.26	0.33	0.27
P-Dias002 *Leptostylis* cf. *longimana*	**35**	0.01	0.30	0.30	0.29	0.00	0.25	0.30	0.00	0.30	0.29	0.27	0.27		0.00	0.00	0.00	0.00	0.01	0.00	0.00	0.33	0.22
P-Dias003 *Leptostylis* cf. *longimana*	**36**	0.00	0.30	0.30	0.28	0.00	0.24	0.30	0.00	0.30	0.28	0.26	0.26	0.00		0.00	0.00	0.00	0.00	0.00	0.01	0.33	0.21
P-Dias004 *Leptostylis* cf. *longimana*	**37**	0.01	0.30	0.30	0.29	0.00	0.25	0.30	0.00	0.30	0.29	0.27	0.27	0.00	0.00		0.00	0.00	0.01	0.00	0.00	0.33	0.22
P-Dias027 *Leptostylis* cf. *longimana*	**38**	0.01	0.30	0.30	0.30	0.00	0.25	0.30	0.00	0.30	0.30	0.26	0.26	0.00	0.00	0.00		0.00	0.01	0.00	0.00	0.33	0.22
P-Dias028 *Leptostylis* cf. *longimana*	**39**	0.00	0.30	0.29	0.28	0.00	0.25	0.30	0.00	0.29	0.28	0.26	0.26	0.00	0.00	0.00	0.00		0.00	0.00	0.01	0.33	0.22
P-Dias030 *Leptostylis* cf. *longimana*	**40**	0.01	0.30	0.29	0.28	0.01	0.24	0.30	0.01	0.29	0.28	0.27	0.27	0.01	0.00	0.01	0.01	0.00		0.00	0.01	0.33	0.21
P-Dias031 *Leptostylis* cf. *longimana*	**41**	0.00	0.30	0.30	0.29	0.00	0.25	0.30	0.00	0.30	0.29	0.27	0.27	0.00	0.00	0.00	0.00	0.00	0.00		0.01	0.33	0.22
P-Dias032 *Leptostylis* cf. *longimana*	**42**	0.01	0.29	0.29	0.28	0.00	0.25	0.29	0.01	0.29	0.28	0.26	0.26	0.00	0.01	0.00	0.00	0.01	0.01	0.01		0.33	0.22
seq5 *Petalosarsia declivis*	**43**	0.33	0.31	0.31	0.30	0.33	0.34	0.30	0.33	0.31	0.30	0.33	0.33	0.33	0.33	0.33	0.33	0.33	0.33	0.33	0.33		0.32
ICE1-Dia025 *Leptostylis* sp. 1	**44**	0.22	0.29	0.29	0.27	0.22	0.24	0.30	0.22	0.29	0.27	0.27	0.27	0.22	0.21	0.22	0.22	0.22	0.21	0.22	0.22	0.32	
