# Supplementary material for: Adding pieces to the puzzle: insights into diversity and distribution patterns of Cumacea (Crustacea: Peracarida) from the deep North Atlantic to the Arctic Ocean
Source: PeerJ. 2021 Nov 11;9:e12379. doi: 10.7717/peerj.12379 (PMC8590803; doi:10.7717/peerj.12379)
Supplement: Supplemental Information 18 — Uncorrected intra- and interspecific pairwise genetic distance range (p-distance) of putative species of the cumacean families Ceratocumatidae and Lampropidae, delimited ABGD groups based on the applied threshold of P = 0.01–0.1 (13 groups) and the groups’ nearest neighbor. [file peerj-09-12379-s018.pdf]

ABGD Group (P = 0.01-0.1)	Putative species	*N*	Intra-specific min	Intra-specific max	Inter-specific min	Inter-specific max	Nearest neighbor (min *p*-distance)
Lam01	*Alamprops augustinensis*	1	NA	NA	0.26	0.32	Lam04, Lam09
Lam02	*Chalarostylis elegans*	2	0.00	0.00	0.18	0.31	Lam08
Lam04	*Hemilamprops assimilis*	1	NA	NA	0.13	0.34	Lam09
Lam05-A	*Hemilamprops cristatus* (seq81/ seq82/ ICE1-Lam018)	3	0.00	0.02	0.13	0.31	Lam08
Lam05-B	*Hemilamprops cf. cristatus* (ICE1-Lam002)	1	NA	NA	0.20	0.32	Lam13
Lam06	*Hemilamprops aff. diversus*	3	0.00	0.00	0.27	0.32	Lam02, Lam08
Lam07	*Hemilamprops pterini*	2	0.00	0.00	0.13	0.28	Lam05, Lam06
Lam08	*Hemilamprops roseus*	1	NA	NA	0.13	0.32	Lam04
Lam10	*Hemilamprops* sp. 2	1	NA	NA	0.24	0.30	Lam12
Lam11	*Hemilamprops uniplicatus*	2	0.00	0.00	0.17	0.30	Lam08, Lam05
Lam12	*Mesolamprops denticulatus*	1	NA	NA	0.20	0.31	Lam06
Lam13	*Platysympus typicus*	4	0.00	0.01	0.25	0.31	Lam05, Cer01
Cer01	*Cimmerius reticulatus*	1	NA	NA	0.25	0.33	Lam14

N = Number of sequences
