# Supplementary material for: Adding pieces to the puzzle: insights into diversity and distribution patterns of Cumacea (Crustacea: Peracarida) from the deep North Atlantic to the Arctic Ocean
Source: PeerJ. 2021 Nov 11;9:e12379. doi: 10.7717/peerj.12379 (PMC8590803; doi:10.7717/peerj.12379)
Supplement: Supplemental Information 19 [file peerj-09-12379-s019.pdf]

Ceratocumatidae and Lampropidae

		1	2	3	4	5	6	7	8	9	10	11	12	13	14	15	16	17	18	19	20	21	22	23
seq87 *Alamprops augustinensis*	**1**		0.26	0.27	0.27	0.26	0.28	0.28	0.29	0.27	0.30	0.28	0.27	0.29	0.29	0.28	0.27	0.32	0.28	0.27	0.28	0.28	0.30	0.30
seq80 *Hemilamprops assimilis*	**2**	0.26		0.31	0.31	0.13	0.30	0.30	0.31	0.28	0.30	0.27	0.29	0.31	0.31	0.28	0.28	0.31	0.29	0.30	0.30	0.26	0.30	0.30
seq81 *Hemilamprops cristatus*	**3**	0.27	0.31		0.00	0.31	0.18	0.18	0.31	0.24	0.26	0.13	0.21	0.31	0.31	0.13	0.27	0.27	0.21	0.02	0.28	0.23	0.26	0.26
seq82 *Hemilamprops cristatus*	**4**	0.27	0.31	0.00		0.31	0.18	0.18	0.31	0.24	0.26	0.13	0.21	0.31	0.31	0.13	0.27	0.27	0.21	0.02	0.28	0.23	0.26	0.26
seq83 *Hemilamprops rosea*	**5**	0.26	0.13	0.31	0.31		0.30	0.30	0.31	0.29	0.30	0.29	0.29	0.31	0.31	0.29	0.30	0.32	0.30	0.31	0.30	0.29	0.31	0.30
seq84 *Hemilamprops uniplicatus*	**6**	0.28	0.30	0.18	0.18	0.30		0.00	0.28	0.21	0.27	0.17	0.19	0.28	0.28	0.17	0.24	0.28	0.20	0.17	0.28	0.22	0.27	0.27
seq85 *Hemilamprops uniplicatus*	**7**	0.28	0.30	0.18	0.18	0.30	0.00		0.28	0.21	0.27	0.17	0.19	0.28	0.28	0.17	0.24	0.28	0.20	0.17	0.28	0.22	0.27	0.27
ICE1-Lam001 *Hemilamprops* cf. *diversus*	**8**	0.29	0.31	0.31	0.31	0.31	0.28	0.28		0.32	0.29	0.27	0.27	0.00	0.00	0.27	0.30	0.31	0.27	0.29	0.33	0.31	0.30	0.30
ICE1-Lam002 *Hemilamprops* cf. *cristatus*	**9**	0.27	0.28	0.24	0.24	0.29	0.21	0.21	0.32		0.28	0.24	0.23	0.32	0.32	0.24	0.26	0.28	0.24	0.23	0.27	0.20	0.28	0.28
ICE1-Lam004 *Platysympus typicus*	**10**	0.30	0.30	0.26	0.26	0.30	0.27	0.27	0.29	0.28		0.27	0.29	0.29	0.29	0.28	0.27	0.00	0.30	0.25	0.25	0.29	0.00	0.01
ICE1-Lam005 Hemilamprops pterini	**11**	0.28	0.27	0.13	0.13	0.29	0.17	0.17	0.27	0.24	0.27		0.18	0.27	0.27	0.00	0.25	0.28	0.19	0.13	0.26	0.21	0.28	0.28
ICE1-Lam009 *Chalarostylis elegans*	**12**	0.27	0.29	0.21	0.21	0.29	0.19	0.19	0.27	0.23	0.29	0.18		0.27	0.27	0.19	0.27	0.29	0.00	0.20	0.28	0.22	0.30	0.30
ICE1-Lam010 *Hemilamprops* cf. *diversus*	**13**	0.29	0.31	0.31	0.31	0.31	0.28	0.28	0.00	0.32	0.29	0.27	0.27		0.00	0.27	0.30	0.31	0.27	0.29	0.33	0.31	0.30	0.30
ICE1-Lam011 *Hemilamprops* cf. *diversus*	**14**	0.29	0.31	0.31	0.31	0.31	0.28	0.28	0.00	0.32	0.29	0.27	0.27	0.00		0.27	0.30	0.31	0.27	0.29	0.33	0.31	0.30	0.30
ICE1-Lam013 *Hemilamprops pterini*	**15**	0.28	0.28	0.13	0.13	0.29	0.17	0.17	0.27	0.24	0.28	0.00	0.19	0.27	0.27		0.25	0.28	0.19	0.13	0.26	0.21	0.28	0.28
ICE1-Lam015 *Hemilamprops* sp. 2	**16**	0.27	0.28	0.27	0.27	0.30	0.24	0.24	0.30	0.26	0.27	0.25	0.27	0.30	0.30	0.25		0.27	0.27	0.26	0.29	0.28	0.29	0.29
ICE1-Lam016 *Platysympus typicus*	**17**	0.32	0.31	0.27	0.27	0.32	0.28	0.28	0.31	0.28	0.00	0.28	0.29	0.31	0.31	0.28	0.27		0.29	0.25	0.26	0.29	0.01	0.01
ICE1-Lam017 *Chalarostylis elegans*	**18**	0.28	0.29	0.21	0.21	0.30	0.20	0.20	0.27	0.24	0.30	0.19	0.00	0.27	0.27	0.19	0.27	0.29		0.20	0.29	0.23	0.31	0.31
ICE1-Lam018 *Hemilamprops* cf. *cristatus*	**19**	0.27	0.30	0.02	0.02	0.31	0.17	0.17	0.29	0.23	0.25	0.13	0.20	0.29	0.29	0.13	0.26	0.25	0.20		0.26	0.23	0.26	0.26
ICE1-Cer003 *Cimmerius reticulatus*	**20**	0.28	0.30	0.28	0.28	0.30	0.28	0.28	0.33	0.27	0.25	0.26	0.28	0.33	0.33	0.26	0.29	0.26	0.29	0.26		0.31	0.25	0.25
seq88 *Mesolamprops denticulatus*	**21**	0.28	0.26	0.23	0.23	0.29	0.22	0.22	0.31	0.20	0.29	0.21	0.22	0.31	0.31	0.21	0.28	0.29	0.23	0.23	0.31		0.29	0.29
seq89 *Platysympus typicus*	**22**	0.30	0.30	0.26	0.26	0.31	0.27	0.27	0.30	0.28	0.00	0.28	0.30	0.30	0.30	0.28	0.29	0.01	0.31	0.26	0.25	0.29		0.01
seq90 *Platysympus typicus*	**23**	0.30	0.30	0.26	0.26	0.30	0.27	0.27	0.30	0.28	0.01	0.28	0.30	0.30	0.30	0.28	0.29	0.01	0.31	0.26	0.25	0.29	0.01	
